# Neuromuscular electrical stimulation during maximal voluntary contraction: a Delphi survey with expert consensus

**DOI:** 10.1007/s00421-023-05232-1

**Published:** 2023-05-29

**Authors:** J. O. Osborne, J. Tallent, O. Girard, P. W. Marshall, D. Kidgell, R. Buhmann

**Affiliations:** 1 School of Sport Sciences, UiT The Arctic University of Norway, Medisin- Og Helsebygget, UiT, 9037 Tromsø, Norway; 2 School of Sport, Rehabilitation and Exercise Sciences, University of Essex, Colchester, UK; 3 Monash Exercise Neuroplasticity Research Unit, Department of Physiotherapy, School of Primary and Allied Health Care, Faculty of Medicine, Nursing and Health Science, Monash University, Melbourne, VA Australia; 4 School of Human Sciences (Exercise and Sport Sciences), The University of Western Australia, Perth, WA Australia; 5 School of Health Sciences, Western Sydney University, Penrith, NSW Australia; 6 Department of Exercise Science, University of Auckland, Auckland, New Zealand; 7 School of Health and Behavioural Sciences, University of the Sunshine Coast, Maroochydore, QLD Australia

**Keywords:** Nerve, Neurophysiology, Interpolated twitch, Voluntary activation

## Abstract

**Purpose:**

The use of electrical stimulation to assess voluntary activation of muscle/s is a popular method employed in numerous exercise science and health research settings. This Delphi study aimed to collate expert opinion and provide recommendations for best practice when using electrical stimulation during maximal voluntary contractions.

**Methods:**

A two-round Delphi study was undertaken with 30 experts who completed a 62-item questionnaire (Round 1) comprising of open- and closed-ended questions. Consensus was assumed if ≥ 70% of experts selected the same response; such questions were removed from the subsequent Round 2 questionnaire. Responses were also removed if they failed to meet a 15% threshold. Open-ended questions were analysed and converted into closed-ended questions for Round 2. It was assumed there was no clear consensus if a question failed to achieve a ≥ 70% response in Round 2.

**Results:**

A total of 16 out of 62 (25.8%) items reached consensus. Experts agreed that electrical stimulation provides a valid assessment of voluntary activation in specific circumstances, such as during maximal contraction, and this stimulation can be applied at either the muscle or the nerve. Experts recommended using doublet stimuli, self-adhesive electrodes, a familiarisation session, real-time visual or verbal feedback during the contraction, a minimum current increase of + 20% to ensure supramaximal stimulation, and manually triggering stimuli.

**Conclusion:**

The results of this Delphi consensus study can help researchers make informed decisions when considering technical parameters when designing studies involving electrical stimulation for the assessment of voluntary activation.

**Supplementary Information:**

The online version contains supplementary material available at 10.1007/s00421-023-05232-1.

## Introduction

The assessment of muscle activation during voluntary contraction is common in sport science, exercise research, and health settings (Behm et al. 1996, 2001; Millet and Lepers 2004; O’Brien et al. 2021). The extent of full muscle activation can be determined by applying an electrical stimulus to a peripheral nerve or muscle during a maximum voluntary contraction of a muscle group (i.e. knee extensors, plantar flexors, elbow flexors) (Shield and Zhou 2004). If motor units are not fully recruited or are discharging sub-maximally, the exogenous electrical stimulus will cause a measurable rise in muscle force produced. A comparison is then made between maximal voluntary force (i.e. preceding the electrical stimulation) and the evoked force, to determine the level of voluntary activation (Merton 1954). The level of voluntary activation can be quantified using either the interpolated twitch method $\left(\text{\% voluntary activation}=\left(1-\frac{\text{superimposed twitch}}{\text{resting potentiated twitch}}\right)\times 100\right)$ or the central activation ratio $(\text{CAR}= \frac{\mathrm{maximal voluntary contraction force}}{(\text{maximal voluntary contraction force }+\text{ maximal stimulated force})})$. With the interpolated twitch method, the resting potentiated twitch is typically triggered three to five seconds following a maximal voluntary contraction. Whilst the resting twitch can be elicited prior to the contraction, this results in a smaller twitch amplitude and an overestimation of the percentage of voluntary activation (Shield and Zhou 2004). Quantifying the completeness of voluntary muscle activation is important, as the level of voluntary activation is related to changes in muscle function. For example, changes in voluntary activation underpin dysfunction observed in older participants (Clark and Taylor 2011), persistent strength loss following injury (Hart et al. 2010), and adaptations to training (Hortobágyi and Maffiuletti 2011).

Despite widespread measurement of voluntary activation using the electrical stimulation method, there is no consensus regarding the optimal implementation or interpretation of this procedure (de Haan et al. 2009; Taylor 2009). For example, there remains disagreement regarding the construct validity of this method for quantification of voluntary muscle activation: some researchers argue that this method can only truly provide a qualitative assessment (Horstman 2009), whilst others believe it provides a sensitive measure of voluntary activation (Taylor 2009). In addition, several methodological considerations could affect the validity and calculation of voluntary activation. Modifications in the number of stimuli (i.e. single, double or train), pulse width, pulse frequency or electrode placement may result in different estimates of voluntary activation (Shield and Zhou 2004). Furthermore, there is no clear agreement on the definition and appropriate interpretation of results collected to reflect muscle activation. For example, the outcome measure calculated from the comparison of evoked and voluntary forces, is referred to as either percentage voluntary activation (Kean et al. 2010), central activation ratio (Pietrosimone and Ingersoll 2009) or voluntary activation level (Beltman et al. 2004). There is also diverging views in the interpretation of the measure, with suggestions it may (Clark et al. 2007) or may not (Horstman 2009) provide evidence of limited central drive to the motoneuron pool.

The Delphi method is a structured scientific process that utilises repeated rounds of expert discussion and opinion to develop consensus regarding specific topics or methodology, often where the existing published literature remains limited, contradictory or ambiguous (Beiderbeck et al. 2021). The Delphi technique is commonly used in the health and medical literature, and is increasingly popular in exercise science research to determine best practice within sub disciplines such as training (Manca et al. 2021), measurement (Robertson et al. 2017; Moreira et al. 2017), injury/rehabilitation (Fredriksen et al. 2020; McCall et al. 2020) or ergogenic aids (Shannon et al. 2022).

Currently, there are no published recommendations that provide a complete and tailored methodological guidance (e.g. provide specific suggestions on how to adjust technical parameters based on different muscles or settings) to researchers who are interested in assessing voluntary activation of muscle. It should be noted that several papers have provided useful commentary on different aspects of the method (de Haan et al. 2009; Horstman 2009; Taylor 2009; Maffiuletti 2010) however, none have published detailed guidance for optimizing technical parameters (e.g. electrode placement and size, pulse characteristics) during investigations. Expert consensus, derived from a Delphi study, would enhance consistency in the practical application of methods (e.g. more direct comparison of study results) and increase the methodological quality employed in studies. The aim of this Delphi study was to collate expert opinion to provide recommendations for best practice (e.g. optimal stimulation parameters and interpretation of results) when using electrical stimulation during maximal voluntary contractions.

## Methods

### Expert panel

For this Delphi study, ‘expert’ participants were required to have demonstrable experience using voluntary activation methodology, quantified by a minimum number of topic-relevant publications in the past 20 years: ≥ 3 studies as a first author, and/or ≥ 10 studies as a co-author. To determine potential experts that fit this inclusion criterion, the PubMed database was searched using terms related to voluntary activation methodology, using a combination of terms including “voluntary activation”, “twitch interpolation”, “electrical stimulation”, “central activation”, “muscle stimulation” and/or “nerve stimulation”. All articles must have been published in English and during the 20-year span from 1 January 2001 to 31 December 2021. The search was completed on 13 January 2021 and results were saved, cleaned and exported into the statistical software, R (R Core Team 2023). Custom code was written to extract authors who published either: (a) ≥ 3 studies as a first author, and/or (b) ≥ 10 studies as a co-author, which aligns with recommendations that Delphi experts should have considerable relevant expertise in the area of enquiry (Jünger et al. 2017). A total of 89 potential researchers considered as ‘experts’ in this area were identified. Potential participants were recruited through personal industry contacts and by cold contacting via publicly available e-mail addresses. Of the 89 identified experts, 5 were uncontactable, so invitations were sent to 84 experts, with 30 agreeing to participate in Round 1. This study was approved by the Norwegian Centre for Research Data (Reference Number: 911837) and all participants were fully informed of the study requirements before they provided written consent at the start of Round 1.

### Development of questionnaire

The initial Delphi questionnaire was developed by the study authors and comprised 33 main questions, split into subgroups, for a total of 62 unique questions. The questionnaire addressed four topic areas: (i) definitions and validity (questions 1–3); (ii) stimulation configuration and parameters (questions 4–10); (iii) reliability and familiarisation (questions 11–19); and (iv) analysis, interpretation and other methodological considerations (questions 20–33). The reader is referred to Online Resource 1 to view a copy of Round 1 questionnaire. Questions within each topic area were devised by examining relevant extant commentaries and narrative reviews of voluntary activation methodology (Shield and Zhou 2004; de Haan et al. 2009; Horstman 2009; Taylor 2009), prominent experimental studies using this methodology (Allen et al. 1995; Behm et al. 1996; Urbach et al. 2001), as well as iterative input from the study authors. Given the lack of published technical recommendations for voluntary activation methodology, iterative changes and modifications by the study authors were key aspects for the development and refinement of this Delphi questionnaire. The questionnaire was also extensively drafted, and pilot-tested by the study authors prior to the finalisation and dissemination of the questionnaire for Round 1. We attempted to create questions that were closed-ended and/or could be answered using a quantitative metric (e.g. scales or ratings). However, this was not always possible, due to the modifications made to technical parameters in specific experimental settings. Therefore, several questions required an answer using an open-ended response. Open-ended responses were also included in Round 1 of the questionnaire as a supplement to questions answered with scales or ratings, with the intention to capture any additional opinions or beliefs that may not have been provided with the initial selection of answers available. This also informed the development of a revised, quantifiable scale/rating for the subsequent Round 2 of the Delphi study (see Online Resource 2 for the Round 2 questionnaire).

### The Delphi process

As described by Jünger et al. (2017), a Delphi study involves (i) identification of relevant experts for participation; (ii) development of a questionnaire to collect information on the method; (iii) resending the questionnaire to identified experts several times, with the questionnaire adjusted based on previous responses between rounds; and (iv) the collation of data from the final round of the questionnaire.

An online survey website, Nettskjema, was used to host the questionnaire. Nettskjema was developed by the University of Oslo and is commonly used for online survey data collection in Norway (https://nettskjema.no/). All 84 identified experts were contacted via e-mail with a link to the Delphi questionnaire, as well as study information, such as the aim to develop a methodological consensus for the scientific community. Round 1 of the questionnaire was open for 8 weeks (March–May 2022), and participants were emailed a reminder request part-way through this period. Round 2 was open for 16 weeks (June–September 2022) with a mid-point reminder e-mail sent to participants who had not yet completed the questionnaire.

### Round 1

For Round 1, 25 of 33 main questions (78%) required experts to select from a range of pre-determined responses (e.g. multiple choice questions or rating on a scale). Methodological validity questions used a five-step Likert scale, from 1 *‘completely limits the validity of the method’* to 5 *‘has no influence on validity’*. An open text box was provided for 14 of the multiple choice questions, which allowed subjects the opportunity to provide a detailed open-ended answer relating to the specific statement if the set range of answers was not adequate. Responses to each opened-ended questions were analysed for common themes, which were then used to develop a set of possible responses for Round 2. For all closed-ended questions, a consensus threshold of agreement was defined as ≥ 70% of respondents selecting the same response, as has been used by previous Delphi surveys in exercise science (Kleynen et al. 2014; van der Horst et al. 2017; McCall et al. 2020). Questions that had reached the threshold for consensus were removed from Round 2. Response options that failed to reach a minimum threshold of ≤ 15% were also removed for Round 2, leaving a more relevant range of predetermined answers. This minimum cut-off threshold of 15% was established following consultation amongst the authors with previous experience conducting Delphi studies (Wells et al. 2014a, b; Manca et al. 2021; Brunoni et al. 2022). No compulsory responses were included in Round 1, to ensure that experts were not forced to make a selection they did not fully agree with.

### Round 2

Following completion of Round 1, questions with multiple responses (e.g. different electrode dimensions) were revised and categorised into simplified groups (e.g. small, medium and large electrode sizes). Similarly, five-step Likert scale questions were reduced to a three-step scale (i.e. none/minor effect on validity; moderate/major effect on validity; completely limits validity). Open-ended questions (*n* = 5) were converted into closed-ended ones, with the range of responses taken from the common themes observed in the Round 1 of responses for each open-ended question. Multiple-selection questions, where more than one response could be selected, were altered to permit only a single response (e.g. preferred intratester reliability measure) for Round 2. The possibility of providing an open-ended answer was also removed from the pre-determined responses for Round 2, to limit extraneous dilution of the responses. However, it was recognised that the use of compulsory responses may have forced a participant to choose a suboptimal answer, and as such, no questions were set as compulsory to complete. Due to this choice, participants’ who disagreed with all available response options were able to skip the relevant question and continue with the questionnaire.

The Delphi Round 2 used the modified and revised questionnaire from the initial round, with all 21 main questions of closed-ended type. Like Round 1, a majority consensus was considered when ≥ 70% of experts provided the same response. For questions that did not reach this consensus threshold, it was assumed that there was no clear consensus amongst the experts for that specific question.

### Data analysis

For each round, questionnaire responses were downloaded from Nettskjema to Microsoft Excel (Microsoft Corporation, Redmond, USA) and deidentified. Data were then imported into R software (R Core Team 2023) for analysis. The proportion for each category of quantitative closed-ended question was calculated to determine the achievement of consensus (≥ 70% of respondents), or the possible removal of a response (≤ 15% of respondents), as previously described. Please refer to Online Resource 3 and 4 for all closed-ended results from Round 1 and 2, respectively. Qualitative open-ended questions were cleaned and analysed via text-mining packages: *tidytext* (Silge and Robinson 2016), *SnowballC* (Bouchet-Valat 2020) and *stringr* (Wickham 2022). This analysis of qualitative responses—frequency of word occurrence and strength of relationship to other words—was then used to develop closed-ended multiple response questions for Round 2. All open-ended responses from Round 1 can be viewed in Online Resource 5, and the text-mining analysis in Online Resource 6.

## Results

### Expert demographics and participation

From the 84 experts that were initially contacted, 30 agreed (36%) to participate and completed Round 1 of the questionnaire (male: *n* = 26; female: *n* = 4). Most respondents currently resided in France (27%; *n* = 8), followed by USA (13%; *n* = 4), Switzerland and Canada (10%; *n* = 3 each). See Table 1 for full demographic details. All respondents held a PhD (*n* = 29) or MD (*n* = 1), and the majority were currently employed as either Associate Professor (37%; *n* = 11) or Professor (30%; *n* = 9) at an academic and/or research institution. Collectively, the experts had considerable experience using twitch interpolation to assess voluntary activation (mean ± SD = 17.4 ± 7.4 years [range 6–30 years]) and had, on average, published 13 ± 9 papers relevant to this topic over the past 20 years (2001–2021; from the PubMed search described in *Expert panel* section). Of the 30 experts who completed Round 1, 26 also completed Round 2 (response rate of 87%).

**Table 1:** Expert panel description from Delphi Round 1 (*n* = 30)

Sex	*n* (%)	Location	*n* (%)	Job Title	*n* (%)	Degree	*n*	Field/s of research	*n*
Female	4 (13%)	Australia	2 (7%)	Professor	9 (30%)	PhD	29	Sport/Exercise Science	21
Male	26 (87%)	Belgium	1 (3%)	Associate Professor	11 (37%)	Medical degree	1	Neurophysiology	14
		Brazil	1 (3%)	Assistant Professor	3 (10%)			Physiology	10
		Canada	3 (10%)	Senior Research Fellow	2 (7%)			Biomechanics	5
		Denmark	1 (3%)	Postdoctoral Research Fellow	1 (3%)			Physiotherapy	3
		Finland	1 (3%)	Senior Lecture	1 (3%)			Medicine	3
		France	8 (27%)	Lecturer	1 (3%)			Other	2
		Germany	2 (7%)	Scientist	1 (3%)				
		Italy	1 (3%)	Professor Emeritus	1 (3%)				
		The Netherlands	1 (3%)						
		Switzerland	3 (10%)						
		The United Kingdom	2 (7%)						
		The United States of America	4 (13%)						

### Definitions and validity

Experts agreed the method provides a valid assessment of activation (or inactivation) but only in specific instances, such as in a clinical population (Table 2). Experts also agreed that both muscle and nerve stimulation could be used to provide a valid assessment of voluntary activation. Although there was no consensus on other questions relating to definitions or validity, the preferred term used to describe the outcome measure was found to be either ‘voluntary activation’ (*n* = 12, 48%) or ‘voluntary activation level’ (*n* = 13, 52%) in Round 2.

**Table 2:** Recommendations for investigators based on achievement of expert panel consensus

	No. of respondents in agreement	% of respondents in agreement	Round consensus achieved
**Definitions and validity**			
Muscle and nerve stimulation provide a valid assessment of voluntary activation	22	73	Round 1
**Stimulation configuration and parameters**			
Self-adhesive stimulation pads preferred to a stimulating pen of metal plate electrodes	24	80	Round 1
Twin (doublet stimulation) required for valid assessment of voluntary activation	19	73	Round 2
**Reliability and familiarisation**			
A single familiarisation session is sufficient to achieve valid and reliable measures during experimental sessions	23	76	Round 1
Verbal encouragement and feedback from force/torque traces are equally acceptable forms of feedback	20	77	Round 2
Real-time feedback improves the validity and reliability of voluntary activation estimates	21	81	Round 2
Increase the current eliciting a maximal twitch response by 20% during voluntary activation assessments	18	70	Round 2
**Analysis, interpretation, and other methodological considerations**			
A single contraction is sufficient to estimate voluntary activation	26	87	Round 1
Twitch interpolation (comparing the difference between maximal voluntary and maximal stimulated force to resting potentiated twitch force) provides a more valid estimate of voluntary activation than the central activation ratio (comparing maximal voluntary force against maximal voluntary force and maximal stimulated force)	22	73	Round 1
Stimulation will always activate antagonists, although this only has a minor effect on validity	21	70	Round 1
Manual triggering of the stimulus is the most consistent and accurate method of stimulus administration	20	77	Round 2

Some participants did not complete both rounds of the questionnaire (Round 1: *n* = 30; Round 2: *n* = 26). Percentages will correspond to a different absolute response rate between rounds

### Stimulation configuration and parameters

Experts agreed on the use of self-adhesive electrodes for stimulation, as opposed to a stimulating pen or metal plate electrodes. Experts agreed twin (doublet) stimuli were required for a valid assessment of voluntary activation. Experts did not reach consensus on other technical parameters of stimulation across the two survey rounds (pulse width, anode/cathode size and strategies for reducing pain/discomfort).

### Reliability and familiarisation

Experts agreed that participants could provide a valid and reliable assessment of voluntary activation after a single familiarisation session. Experts agreed that verbal encouragement (e.g. *“when you contract, we want you to contract as hard and as fast as possible and continue contracting through the stimulus”*) and feedback from force/time traces were acceptable forms of feedback for participants when assessing voluntary activation. Experts also agreed that providing real-time feedback (i.e. as participants are contracting) improves the validity and reliability of voluntary activation assessments. Finally, experts agreed that the current should be increased by 20% (from the current that elicits the maximal twitch response) to make the stimulus supramaximal during investigations. In Round 2, we noted a high proportion of missing data (i.e. no response) for questions related to size/type electrodes when stimulating the common peroneal nerve/dorsiflexors (*n* = 8 [31%] and *n* = 7 [27%] for the cathode and anode, respectively).

### Analysis, interpretation and other methodological considerations

Experts agreed that a single contraction is suitable for measuring voluntary activation and that the twitch interpolation method (i.e. $\text{\% voluntary activation}=\left(1-\frac{\text{superimposed twitch}}{\text{resting potentiated twitch}}\right)\times 100$) provides a more valid assessment (a more accurate measurement of voluntary muscle recruitment) than the central activation ratio, (i.e. $\text{CAR}= \frac{\text{maximal voluntary contraction force }}{(\text{maximal voluntary contraction force }+\text{ maximal stimulated force})})$. Experts also agreed that stimulation will always result in partial activation of antagonist muscles, yet this only has a minor effect on validity of the method. Experts agreed that the measure can provide a meaningful assessment of voluntary activation in resistance trained athletes (*n* = 25, 83%), aerobically trained athletes (*n* = 25, 83%), healthy younger (< 60 years, *n* = 26, 87%) and healthy older (> 60 years, *n* = 25, 83%) individuals. Experts also agreed that the measurement provides a meaningful assessment of voluntary activation following training interventions (e.g. resistance training *n* = 27, 90%) and exhaustive exercise (*n* = 27, 90%). Experts agreed that manual triggering of the stimulus once the force trace reaches a visible plateau is the most consistent and accurate method of stimulus administration and that a lack of transfer from lab-based deficits to real-world movements has a moderate-major impact on the validity of the measure (*n* = 22, 85%).

## Discussion

This two-round Delphi consensus study surveyed a group of experts and collated their responses regarding best practice methodology when utilising neuromuscular electrical stimulation for the assessment of voluntary activation. The findings from this study are discussed within the four identified themes: (i) definitions and validity; (ii) stimulation configuration and parameters; (iii) reliability and familiarisation; and (iv) analysis, interpretation and other methodological considerations.

Consensus was reached on a total of 16 out of 62 (25.8%) unique items after two survey rounds. Most (90%) experts agreed that the electrical stimulation method provided a valid assessment of voluntary activation, provided participants are contracting at their perceived voluntary maximum. They also concluded that nerve and muscle stimulations provide a valid assessment of voluntary activation (73%). Previously, there has been some debate surrounding the validity of the method for assessing voluntary activation (de Haan et al. 2009; Horstman 2009; Taylor 2009). However, the consensus in the present study indicates that researchers feel confident that increases in force following electrical stimulation represent a suboptimal activation capacity (provided it is a true maximum contraction).

There was no consensus reached for the appropriate term used to describe the outcome measure, with experts split between either ‘voluntary activation’ (46%) or ‘voluntary activation level’ (50%). As such, it is recommended that researchers should regard both terms as suitable and interchangeable to describe muscle activation, when used in academic or clinical settings. Similarly, there was disagreement for the description of the outcome measure, with some experts believing that the outcome should describe the level of force produced compared with maximal force (58%). In contrast, other experts considered that the outcome could only describe the level of inactivation (i.e. it can determine sub-maximal activation, but is inaccurate; 38%). Such dichotomy has been previously debated within the literature (Horstman 2009), and further investigation is required to determine whether a precise level of voluntary activation can be measured using the method.

Regarding technical parameters for stimulation, experts agreed that self-adhesive electrodes (80%) and doublet electrical stimuli (73%) were most suitable for valid and reliable assessments of voluntary activation. As a preference for doublet stimuli has also been previously noted in the literature, we recommend that researchers implement doublet stimuli for voluntary activation assessments (Shield and Zhou 2004; Bampouras et al. 2006). Recent evidence has encouraged the use of wide electrical pulses (e.g. 0.4–1 ms) (Collins 2007; Maffiuletti et al. 2018) to maximize evoked force. However, there was no consensus amongst experts regarding the optimal pulse width. This may reflect the inconsistency and variability within the literature, as many previous studies have used different pulse widths (e.g. 0.1–1 ms) (Rozand et al. 2017). As the pulse width used in voluntary activation assessments could be influenced by the volume conductor properties of the nerve/muscle being stimulated, a greater distance between the stimulating electrode and tissue necessitates a wider/stronger pulse (Petrofsky 2008). Additionally, this disagreement in pulse width may also reflect the strategies used by investigators (i.e. manipulation of pulse width, current intensity and/or electrode size) to minimize participants’ pain and discomfort from electrical stimulation (Jeon and Griffin 2018). Therefore, investigators may decrease pulse width and increase current intensity (or vice versa) to reduce stimulation-associated discomfort. This is an important consideration as anticipation of pain can result in a sub-maximal voluntary contraction (and by extension an underestimation of muscle activation) (Button and Behm 2008). We did not note any common themes in the Round 1 open-ended questions, when asking experts about strategies they utilise to reduce the pain/discomfort associated with stimulation. Some strategies, such as pulse width manipulation and appropriate familiarisation, were apparent, although the frequency of these responses did not meet the threshold for consensus. Further work should therefore determine specific strategies, tailored for different populations, for reducing the pain/discomfort associated with stimulation, whilst maintaining a valid measure of voluntary activation.

It is also worth noting the high proportion of missing responses for questions relating to anode and cathode dimensions for stimulation of the common peroneal nerve/dorsiflexors (~ 30%). Interestingly, almost all experts provided a response to anode/cathode dimensions when asked about stimulation of the femoral nerve/quadriceps. This response rate discrepancy between common peroneal nerve/dorsiflexors and femoral nerve/quadriceps may be due to the research experience (or lack thereof) between the different muscle groups. Voluntary activation of the knee extensors is well investigated, and as a result, optimal stimulation parameters for this muscle group are well defined. In comparison, the dorsiflexors are less frequently studied and stimulation parameters are likely to be less understood.

Familiarisation with a measurement or technique is essential to obtain reliable results, with practice session(s) often completed to ensure reproducibility. It is therefore important to verify that participants are thoroughly familiarised with maximal voluntary contraction procedures, including the ability to sustain a maximal effort (force trace reaching a plateau) whilst being stimulated. Experts agreed (76%) that a single familiarisation session is necessary to produce reliable measurements in subsequent experimental testing sessions. Similarly, the use of real-time visual or verbal feedback during the actual contraction was strongly recommended to ensure validity. Experts did not reach consensus on the level of intratester reliability or reliability within a familiarisation session. Several experts noted it was difficult to determine a threshold level of reliability due to sources of variability when using the method (e.g. muscle group investigated and type of contraction used). However, given the number of investigations across different research areas (e.g. sport medicine, physiology, exercise science and rehabilitation) that utilise this method, it remains crucial to determine an acceptable level of reliability. Whilst a universal reliability threshold may be unrealistic, future work should aim to determine reliability values for voluntary activation in specific muscle groups (e.g. plantar flexors, quadriceps etc.), during different contraction types (e.g. rapid and prolonged isometric contractions) and in different populations (e.g. healthy and ageing populations). The reliability of the twitch interpolation method has also been questioned when participants are assessed in a fatigued state (Dotan et al. 2021). A better understanding of the factors underpinning increased variability of voluntary activation during fatigue, and how they can be addressed, is required.

To ensure maximal recruitment of all available motor units, electrical stimulation intensity is often increased above an assumed maximal level (i.e. derived from a twitch ramp procedure). However, this additional increase in current must also be considered against the corresponding possible increase in participant pain/antagonist stimulation. Experts agreed that an additional current increase of at least + 20% above the current intensity that leads to a plateau in peak twitch and/or M-wave amplitudes would ensure supramaximal activation and validity, whilst minimising discomfort. For example, a participant who reached a final twitch ramp current of 100 mA, should be stimulated supramaximally at 120 mA (i.e., + 20%) during subsequent maximal contraction repetitions.

Several methodological considerations can affect the estimate of voluntary activation. Experts agreed (73%) that the interpolated twitch method provides a more valid estimate of voluntary activation (i.e. a truer reflection of voluntary muscle recruitment) compared with the central activation ratio, a view which is consistent with the literature (Shield and Zhou 2004). There was also consensus that antagonist activation will inevitably result from stimulation, although this may only have a minor effect on the validity of the measure. It is suggested that, at maximum contraction, voluntary activation may be limited by antagonist stimulation, resulting in a reduction of voluntary activation estimates by 5–10% of maximal contraction intensities (Taylor 2009). Therefore, we recommend researchers record EMG activity during assessments to determine the extent of antagonist activation, this will help investigators decide if individual trials should be rejected due to antagonist activation. Experts agreed (87%) that a single contraction is suitable for the calculation of voluntary activation (as opposed to averaging several contractions). Conversely, previous studies have often used the average across several contractions, as other variables are also measured (e.g. torque, surface electromyographic activity) (Behm et al. 1996; Place et al. 2010; Kirk and Rice 2017). Additionally, when asked about reliability of the measure, several respondents reported coefficients of variation up to 10% in their investigations. It is interesting that experts agree a single contraction is acceptable for voluntary activation estimates, considering the level of variability reported by respondents. Additional research should consider if the number of contractions analysed may influence the estimate of voluntary activation, with a consideration of contextual factors (i.e. limited time available in fatigue assessment protocols) when doing so. The validity of the twitch interpolation method has been questioned in the context of fatigue (Dotan et al. 2021), and further work is required to understand factors affecting estimates of voluntary activation in this setting.

There was also consensus (77%) that manually triggering the electrical stimulus is the most accurate and consistent stimulation procedure, when compared with automatically triggered stimuli. Considering the potential for human error when manually triggering a stimulus (e.g. not applying the stimulus when maximal voluntary force has been reached), research should investigate whether automatic or manual stimulation affects the estimate of voluntary activation. This preference for manual triggering of the stimuli may reflect the variability in outcomes from the experts’ experiences with automated methods, and/or a lack of skill and experience with establishing automated stimulation procedures. Experts also agreed that the electrical stimulation method provides a meaningful assessment of voluntary activation in individuals who are resistance- or aerobically trained; both healthy older (> 60 years) and younger (< 60 years) adults; and after resistance training interventions or exhaustive exercise (e.g. repeated sprints). There are other instances in the literature where this method has been used to assess voluntary activation in patients (e.g. following stroke (Harris et al. 2001), in cerebral palsy (Stackhouse et al. 2005) and following knee ligament injury (Urbach et al. 2001). The apparent lack of expert consensus for clinical settings may reflect concerns regarding the safety, feasibility or reliability of using this method to assess voluntary activation in these populations. For example, some participants may not feel comfortable with investigators locating specific anatomical landmarks (e.g. the femoral triangle). Therefore, it is important to determine whether there are any perceived or actual barriers that limit the use of electrical stimulating for assessing voluntary activation in certain participant subgroups.

The lack of expert agreement for many questions has highlighted several areas where further investigation is required. For example, there was no consensus on the pulse width for electrical stimulation. It is likely that optimal pulse width differs based upon several factors (i.e. muscle investigated, individual variation in anatomy, pain/discomfort experienced by the participant) (Maffiuletti 2010; Botter et al. 2011) and future research should determine how pulse width should be manipulated during investigations based on these factors. Further work is also needed to understand how electrode size and placement influence estimates of voluntary activation. Changing electrode size and placement can alter the effectiveness, both positively and negatively, of stimulation, as well as pain perception (Alon et al. 1994; Lyons et al. 2004). For example, studies have reported different optimum electrode sizes for the *gastrocnemius* muscle when considering the strength of the elicited contraction and pain experienced by participants (Alon et al. 1994; Lyons et al. 2004). However, these studies used different stimulation parameters, so additional research is essential to understand the complex relationship between individual variables (e.g. electrode size, placement, pulse width, stimulation frequency) and pain perception/strength of the elicited contraction. Future research should also investigate the applicability, safety and/or feasibility of assessing voluntary activation in different clinical populations, to permit the development of clear and relevant recommendations for investigators. Finally, we noted considerable variation in the expert responses for the variables (e.g. coefficient of variation, intraclass correlation coefficient, standard error of the mean) and threshold value (e.g. 3–10% coefficient of variation) of acceptable reliability. If investigators are unsure of how to modify certain parameters based on their study, we encourage them to view our supplementary material containing individual responses (e.g. Online Resource 5). Although there was disagreement on many items, some information within these questions may help inform future investigations.

## Conclusion

The purpose of this Delphi study was to gather expert opinions on voluntary activation measurement and synthesise the results into recommendations for researchers to follow when using electrical stimulation during maximal voluntary contractions. Based on the consensus of responses from the expert panel, the following recommendations were formulated: (i) the term ‘voluntary activation (level)’ is exclusively used to describe the method; (ii) investigators use self-adhesive electrodes and doublet stimuli; (iii) a single practice session is sufficient for appropriate familiarization with the method; (iv) manually triggered stimuli result in the most accurate and valid assessments of voluntary activation (as opposed to automatically triggered stimuli); (v) verbal or real-time visual feedback can be considered equally effective forms of feedback for participants; (vi) selected stimulus intensity (obtained from a ramp protocol) should be further increased by 20% to ensure that stimulus intensity is ‘supramaximal’; and, (vii) analysis of a single contraction is acceptable (provided it is a true maximal effort) for estimating voluntary activation.

## Supplementary Information

Below is the link to the electronic supplementary material.

Supplementary file1 (PDF 1461 KB)

Supplementary file2 (PDF 884 KB)

Supplementary file3 (PDF 216 KB)

Supplementary file4 (PDF 227 KB)

Supplementary file5 (PDF 393 KB)

Supplementary file6 (PDF 311 KB)

## Data Availability

All questionnaire data collected during this study are available in the electronic supplementary material.

## Code availability

The expert panel search results and R code are available at: https://github.com/johnoosborne/delphi_VA.
