# Supplementary material for: Neuromuscular electrical stimulation during maximal voluntary contraction: a Delphi survey with expert consensus
Source: Eur J Appl Physiol. 2023 May 29;123(10):2203–12. doi: 10.1007/s00421-023-05232-1 (PMC10492693; doi:10.1007/s00421-023-05232-1)
Supplement: Supplementary file 1 — Supplementary file1 (PDF 1461 KB) [file 421_2023_5232_MOESM1_ESM.pdf]

Page elements

- Intermediate title
- Text
- Picture
- Page break

Questions

- With text response
- With radio buttons
- With checkboxes
- With drop-down list
- With linear scale

Matrix questions

- With radio buttons
- With checkboxes

Minimize All Maximize All Cancel Save Save and display

Text Minimize Copy Delete

"Twitch interpolation for the assessment of voluntary activation: a Delphi study and methodological consensus"

Purpose of the project

The ability to maximally contract a muscle is dependent on conscious and deliberate voluntary activation (VA) and recruitment of motor neurons via the central nervous system (CNS). However, the specific methodologies used to determine VA using the interpolated twitch technique (ITT) vary across different studies and research groups.

This Delphi study will aim to provide an expert consensus on the appropriate definition, technique and methodology that should be utilised when completing ITT assessments of muscle VA.

Who is responsible for the research project?

John Owen Osborne from UiT The Arctic University of Norway is the project leader responsible for this project. Co-investigators on this project include: Robert Buhmann (University of the Sunshine Coast), Olivier Girard (University of Western Australia), Paul Marshall (University of Auckland), Dawson Kidgell (Monash University) and Jamie Tallent (University of Essex).

Why are you being asked to participate?

You are being asked to participate as you have been identified as an expert in neuromuscular testing. Specifically, you have demonstrated research experience in this field, as evidenced by multiple publications in peer-review journal within the past two decades (≥3 papers as lead author or ≥10 in another position).

What does participation involve for you?

Participation in this Delphi study will involve completing the following online questionnaire. You will be asked to answer 33 questions about the current methodology you believe is 'best-practice' for assessing voluntary activation, as well as your belief around the validity of different techniques for undertaking twitch interpolation. It is anticipated that the 33 questions of this questionnaire will take ~30 minutes to complete.

The project investigators will then collate and anonymise all answers and resend the aggregate questionnaire responses back to all participating experts. You will be then asked to provide feedback on the updated methodology, and this iterative and anonymous process will continue until there is a majority consensus reached among the expert group.

Participation is voluntary

If you chose to participate, you can withdraw your consent at any time without giving a reason. All information about you will then be made anonymous. There will be no negative consequences for you if you chose not to participate or later decide to withdraw.

Text Minimize Copy Delete

Your personal privacy – how we will store and use your personal data

We will only use your personal data for the purpose(s) specified in this information letter. We will process your personal data confidentially and in accordance with data protection legislation (the General Data Protection Regulation and Personal Data Act).

Only the primary investigator (John Osborne) will have access to your personal data (name, age, sex, job position, institution) and all identifying information will be removed or deidentified via recoding, before the questionnaire responses are sent to coinvestigators for aggregation and processing. The key code and personal data will be stored separately in a password-protected file on UiT's research servers. All data processing of questionnaire responses will be completed using the deidentified answers, and the aggregate responses for each round will be anonymous to other experts. No participant in this study will be recognizable or personally identifiable in any publication from this research.

What will happen to your personal data at the end of the research project?
The project is scheduled to end 31 December 2022. Deidentified data will be irreversibly anonymised on this date when the personal code key is deleted.
Your rights
So long as you can be identified in the collected data, you have the right to:

- access the personal data that is being processed about you
- request that your personal data is deleted
- request that incorrect personal data about you is corrected/rectified
- receive a copy of your personal data (data portability), and
- send a complaint to the Data Protection Officer or The Norwegian Data Protection Authority regarding the processing of your personal data

What gives us the right to process your personal data?
We will process your personal data based on your consent.
Based on an agreement with UiT The Arctic University of Norway, NSD – The Norwegian Centre for Research Data AS has assessed that the processing of personal data in this project is in accordance with data protection legislation.
Where can I find out more?
If you have questions about the project, or want to exercise your rights, contact:

- UiT The Arctic University of Norway via Dr. John Owen Osborne (john.owen.osborne@uit.no)
- UiT Data Protection Officer: Joakim Bakkevold, mailadr: personvernombud@uit.no tlf. 77646322, 97691578.
- NSD – The Norwegian Centre for Research Data AS, by email: (personverntjenester@nsd.no) or by telephone: +47 55 58 21 17.

Check boxes - multiple answers per question | Settings | Minimize | Copy | Delete

Consent to participate

By selecting the box below, you are indicating that you:

- Have read and understood the research project information.
- Have had any questions answered to your satisfaction.
- Understand that you are free to withdraw at any time.
- Consent to undertaking the study as per the procedures outlined in the participant information sheet.
- Understand that non-identifiable data from this study may be used in similar, relate[illegible] projects in the future.

If you do not consent to participate in the project, you can not complete this questionnaire.

☑ Mandatory question

Answer options Edit as text — **Preselected**

I consent to participate in this project. ☐

Delete

Add answer option

- Max number of answer options

Page break | Copy | Delete

Full Name | Settings | Minimize | Delete

For logged in users, the name is filled in automatically

Your name (first name and surname)?

This information will only be used by the primary investigator to identify the participating respondents against the initial recruitment email list of experts.

☑ Mandatory question

- Drop-down list - only one answer per question   Settings   Minimize   Copy   Delete

What is your gender?

Description

☑ Mandatory question

Answer options   Edit as text   **Preselected**

Male. ○

Delete

Female. ○

Delete

Other. ○

Delete

Prefer not to respond. ○

Delete

Add answer option

- E-mail address   Settings   Minimize   Delete

For logged-in users, the e-mail address is filled in automatically

What is your preferred e-mail address?

Please note: this information is required so that the aggregated anonymous results of this first round of the Delphi questionnaire can be sent back out to all participants. Only the primary investigator (John Osborne) will have access to your email contact information, and will only use it to send you the next round of the Delphi study questionnaire.

☑ Mandatory question

- Questions - free text answers   Settings   Minimize   Copy   Delete

Please state the country that you primarily reside in:

Description

☐ Mandatory question

Size of response field   Small

- Drop-down list - only one answer per question   Settings   Minimize   Copy   Delete

What is your current job position or title:

Description

☐ Mandatory question

Answer options Edit as text **Preselected**

Lecturer ○
Delete

Senior Lecturer ○
Delete

Assistant Professor ○
Delete

Associate Professor ○
Delete

Professor ○
Delete

Adjunct Professor ○
Delete

Visiting Professor ○
Delete

Postdocotol Research Fellow ○
Delete

Senior Research Fellow ○
Delete

Medical Doctor ○
Delete

Physiotherapist ○
Delete

Other ○
Delete

Add answer option

- Questions - free text answers Settings Minimize Copy Delete

Viewing depends on answers to previous questions.

Please state your current job position or title:

Description

☐ Mandatory question

Size of response field Small

- Radio buttons - only one answer per question Settings Minimize Copy Delete

What are the highest education level that your have completed:

Description

☐ Mandatory question

Answer options Edit as text **Preselected**

Bachelor

Delete

Masters

Delete

Medical degree

Delete

PhD or DSc

Delete

Other

Delete

Add answer option

- Questions - free text answers Settings Minimize Copy Delete

Viewing depends on answers to previous questions.

Please detail any other educational levels that you have completed:

Description

☐ Mandatory question

Size of response field Small

- Number Settings Minimize Copy Delete

How many years have you used twitch interpolation for assessment of voluntary activatic

Please provide the answer in whole years.

☐ Mandatory question

Number of decimal places Kun heltall

Minimum value 0

Maximum value 100

- Check boxes - multiple answers per question Settings Minimize Copy Delete

Please select the type of institution/s you currently work for:

Description

☐ Mandatory question

Answer options Edit as text **Preselected**

Academic and/or research ☐

Delete

Medical hospital ☐

Delete

Private company or industry

Delete

Military

Delete

Other

Delete

Add answer option

- Max number of answer options

Questions - free text answers Settings Minimize Copy Delete

Viewing depends on answers to previous questions.

Please detail any other type of institution/s you currently work for:

Description

☐ Mandatory question

Size of response field Small

Check boxes - multiple answers per question Settings Minimize Copy Delete

Please select the the field of research that you predominantly work in:

Description

☐ Mandatory question

Answer options Edit as text **Preselected**

Sport/exercise science

Delete

Biomechanics

Delete

Physiology

Delete

Physiotherapy

Delete

Neurophysiology

Delete

Biology

Delete

Medicine

Delete

Other

Delete

Add answer option

- Max number of answer options

Questions - free text answers Settings Minimize Copy Delete

Viewing depends on answers to previous questions.

Please detail any other fields of research that you predominantly work in:

Description

☐ Mandatory question

Size of response field Small

- Page break Copy Delete

- Text Settings Minimize Copy Delete

Note:
The purpose of this questionnaire is to develop an expert consensus on the most appropriate methods for electrical stimulation of peripheral nerve/muscle. Please answer all questions in the context of stimulation during a maximum voluntary contraction (and not in the context of H-reflex or conditioned responses (paired pulse) assessments).

- Radio buttons - only one answer per question Settings Minimize Copy Delete

1. Term used to describe the outcome measure.

There are several names given to the outcome variable obtained from the **electrical stimulation** of **muscle/peripheral nerve** during and after maximal voluntary contraction. Indicate the name of the variable you believe is most appropriate:

☐ Obligatorisk spørsmål

**Svaralternativer** Rediger som tekst		**Forvalgt**	
Voluntary activation.	_	○	Sl
Percentage voluntary activation.	_	○	Sl
Voluntary activation level.	_	○	Sl
Central activation level.	_	○	Sl
Peripheral voluntary activation.	_	○	Sl
Peripheral motor voluntary activation.	_	○	Sl
Other (please enter term in the text box below).	_	○	Sl

Legg til svaralternativ

- Spørsmål – fritekstsvar Innstillinger Minimer Kopier Slett

Visning er avhengig av svar på tidligere spørsmål.

1.1. What other term best describes the outcome measure:

Description

Obligatorisk spørsmål

Størrelse på svarfelt Stort

Radioknapper – kun ett svar per spørsmål Innstillinger Minimer Kopier Slett

2. Outcome measure definition

There are several definitions of the outcome variable obtained from electrical stimulation of muscle/nerve during maximum voluntary contraction. Select the most appropriate definition of this variable:

Obligatorisk spørsmål

Svaralternativer Rediger som tekst	Forvalgt	
The amount of force voluntarily produced during contraction as a prop	○	Sl
The level of central/descending drive to the motoneuron pool during vo	○	Sl
The level of inactivation during a maximum voluntary contraction.	○	Sl
The qualitative level of muscle activation during maximum voluntary co	○	Sl
Other (please enter term in the text box below).	○	Sl

Legg til svaralternativ

Spørsmål – fritekstsvar Innstillinger Minimer Kopier Slett

Visning er avhengig av svar på tidligere spørsmål.

2.1. What other definition best describes the outcome variable:

Description

Obligatorisk spørsmål

Størrelse på svarfelt Stort

Radioknapper – kun ett svar per spørsmål Innstillinger Minimer Kopier Slett

3. Validity of twitch interpolation for assessment of VA

Do you consider the twitch interpolation method provides a valid estimate of voluntary activation? Select your belief with one of the options below:

Obligatorisk spørsmål

Svaralternativer Rediger som tekst	Forvalgt	
No, the method does not provide a valid estimate of voluntary activatio	○	Sl
The method provides a valid estimation of voluntary activation, or sub	○	Sl
Yes, the method always provides a valid estimate of voluntary activati	○	Sl

Legg til svaralternativ

Radioknapper – kun ett svar per spørsmål Innstillinger Minimer Kopier Slett

4. Stimulation location – muscle vs. nerve

Assessment of voluntary activation of a muscle group uses electrical stimulation of either the peripheral nerve or muscle belly. Select an answer that best describes your belief of voluntary activation assessment validity using muscle or nerve stimulation.

Note: as stimulation of a peripheral nerve innervating the muscle of interest may not always be possible, or may be difficult (e.g., sciatic nerve), please answer for instances where nerve or muscle stimulation *are* plausible.

☐ Obligatorisk spørsmål

Svaralternativer Rediger som tekst **Forvalgt**

- Neither muscle nor nerve stimulation provides a valid assessment of v ○ Sl
- Only nerve stimulation provides a valid assessment of voluntary activa ○ Sl
- Only muscle belly stimulation provides a valid assessment of voluntar ○ Sl
- Both muscle and nerve stimulation can provide a valid assessment of ○ Sl

Legg til svaralternativ

▪ Radioknapper – kun ett svar per spørsmål Innstillinger Minimer Kopier Slett

Visning er avhengig av svar på tidligere spørsmål.

4.1. Both muscle vs. nerve can provide a valid assessment of voluntary activation.

You have selected that both muscle and nerve stimulation can provide a valid measured of voluntary activation. However, do you believe one location is more valid than the other?

☐ Obligatorisk spørsmål

Svaralternativer Rediger som tekst **Forvalgt**

- No, muscle and nerve stimulation are equally valid. ○ Sl
- Muscle stimulation has a higher validity than nerve stimulation. ○ Sl
- Nerve stimulation has a higher validity than muscle stimulation. ○ Sl

Legg til svaralternativ

▪ Spørsmål – fritekstsvar Innstillinger Minimer Kopier Slett

4.1.1. Stimulation location comments

If you have any additional comments regarding Question 4 (stimulation location), please write them in the text box below:

☐ Obligatorisk spørsmål

Størrelse på svarfelt Lite

▪ Tekst Innstillinger Minimer Kopier Slett

5. Anode and cathode placement
For the following questions, please describe your ideal placement (i.e., describe the location using an anatomical landmark, or, in relation to an anatomical landmark) of the anode and cathode for stimulation of the femoral nerve, tibial nerve and common peroneal nerve.

▪ Spørsmål – fritekstsvar Innstillinger Minimer Kopier Slett

5.1. Anode and cathode placement - Femoral

Please detail your location of the anode AND cathode when stimulation the **femoral nerve**:

☐ Obligatorisk spørsmål

Størrelse på svarfelt Stort

- Spørsmål – fritekstsvar Innstillinger Minimer Kopier Slett

5.2. Anode and cathode placement - Tibial

Please detail your location of the anode AND cathode when stimulation the **tibial nerve**:

☐ Obligatorisk spørsmål

Størrelse på svarfelt Stort

- Spørsmål – fritekstsvar Innstillinger Minimer Kopier Slett

5.3. Anode and cathode placement - Common peroneal

Please detail your location of the anode AND cathode when stimulation the **common peroneal nerve**:

☐ Obligatorisk spørsmål

Størrelse på svarfelt Stort

- Sideskift Kopier Slett

- Nedtrekksliste – kun ett svar per spørsmål Innstillinger Minimer Kopier Slett

6. Pulse width

Stimulation pulse widths typically range from 0.1 to 1ms. A wider pulse width increases the number of motoneurons recruited. However, increasing pulse widths may also increase the pain/discomfort experienced by participants. Indicate the *minimum* pulse width you deem appropriate to assess activation capacity.

☐ Obligatorisk spørsmål

Svaralternativer Rediger som tekst	Forvalgt	
0.1 ms.	○	Sl
0.2 ms.	○	Sl
0.3 ms.	○	Sl
0.4 ms.	○	Sl
0.5 ms.	○	Sl

0.6 ms. Sl

0.7 ms. Sl

0.8 ms. Sl

0.9 ms. Sl

1.0 ms. Sl

Legg til svaralternativ

- Radioknapper – kun ett svar per spørsmål Innstillinger Minimer Kopier Slett

7. Number of stimuli

When applying an electrical stimulus during a voluntary contraction, investigators may apply single or multiple stimuli. Indicate the minimum number of stimuli you believe is optimal for a valid assessment of voluntary activation of muscle.

☐ Obligatorisk spørsmål

Svaralternativer Rediger som tekst **Forvalgt**

1 stimuli. Sl

2 stimuli (doublets). Sl

More than 2 stimuli. Sl

Legg til svaralternativ

- Spørsmål – fritekstsvar Innstillinger Minimer Kopier Slett

Visning er avhengig av svar på tidligere spørsmål.

7.1 More than 2 stimuli

Please describe the number and frequency of stimuli you use, and the reason you use more than two stimuli:

☐ Obligatorisk spørsmål

Størrelse på svarfelt Stort

- Radioknapper – kun ett svar per spørsmål Innstillinger Minimer Kopier Slett

8. Stimulation source

Stimulating electrodes or a stimulating pen may be used to elicit an electrical muscle/nerve stimulus. Which of these methods do you believe is most effective (i.e., results in the most valid and reliable measures) for stimulating muscle/nerve?

☐ Obligatorisk spørsmål

Svaralternativer Rediger som tekst **Forvalgt**

Stimulating electrodes provide the best electrical stimulus for muscle/r Sl

A stimulating pen provides the best electrical stimulus for muscle/nerv Sl

Both stimulating methods are equally effective in eliciting an electrical Sl

Neither stimulating method is effective in electrically stimulating muscl Sl

Legg til svaralternativ

- Radioknapper – kun ett svar per spørsmål Innstillinger

Minimer Kopier Slett

9. Electrode size and type

Please select the type of stimulating electrode you believe is most effective (i.e., is able to elicit a large superimposed twitch, if activation is suboptimal) when applying a peripheral nerve/muscle stimulus:

☐ Obligatorisk spørsmål

Svaralternativer Rediger som tekst		Forvalgt	
Metal plate electrode.	_	○	Sl
Self-adhesive electrode.	_	○	Sl
Stimulating pen.	_	○	Sl

Legg til svaralternativ

- Matrise – ett svar per spørsmål Innstillinger Minimer Kopier Slett

9.1. Electrode size and type - cathode

Electrode size can affect the pain/discomfort experienced by participants following stimulation. While larger electrodes can reduce the pain/discomfort, using electrodes that are too big can also result in stimulation of unwanted muscles.
Please select the most appropriate **cathode** size for stimulation during maximal voluntary contractions for each target nerve/muscle group:

Rader	Obligatorisk	
Femoral nerve/quadriceps.	☐	Slett
Tibial nerve/plantar flexors.	☐	Slett
Common peroneal nerve/dorsiflexors.	☐	Slett

Legg til rad

Kolonner Rediger som tekst	Forvalgt	
Stimulating pen.	○	Sl
3.2 cm (round).	○	Sl
5 cm (round).	○	Sl
3 x 5 cm (width x length).	○	Sl
5 x 5 cm (width x length).	○	Sl
5 x 9 cm (width x length).	○	Sl
5 x 13 cm (width x length).	○	Sl
7.5 x 10 cm (width x length).	○	Sl
7.5 x 13 cm (width x length).	○	Sl
Other.	○	Sl

Legg til kolonne

- Matrise – ett svar per spørsmål Innstillinger Minimer Kopier Slett

9.2. Electrode size and type - anode

Electrode size can affect the pain/discomfort experienced by participants following stimulation. While larger electrodes can reduce the pain/discomfort, using electrodes that are too big can also result in stimulation of unwanted muscles.
Please select the most appropriate **anode** size for stimulation during maximal voluntary contractions for each target nerve/muscle group:

Rader	Obligatorisk	
Femoral nerve/quadriceps.	☐	Slett
Tibial nerve/plantar flexors.	☐	Slett
Common peroneal nerve/dorsiflexors.	☐	Slett

Legg til rad

Kolonner Rediger som tekst	Forvalgt	
Stimulating pen.	○	Sl
3.2 cm (round).	○	Sl
5 cm (round).	○	Sl
3 x 5 cm (width x length).	○	Sl
5 x 5 cm (width x length).	○	Sl
5 x 9 cm (width x length).	○	Sl
5 x 13 cm (width x length).	○	Sl
7.5 x 10 cm (width x length).	○	Sl
7.5 x 13 cm (width x length).	○	Sl
Other.	○	Sl

Legg til kolonne

- Spørsmål – fritekstsvar Innstillinger Minimer Kopier Slett

10. Pain and discomfort during stimulation

Anticipation of pain or discomfort (due to electrical stimulation) may result in a relative inability (compared to a situation without electrical stimulations) to maximally contract the muscle group.
Describe any methodological considerations that should be undertaken to reduce pain/discomfort associated with electrical stimulations (e.g., cueing, stimulating electrode size, pulse width etc.):

☐ Obligatorisk spørsmål

Størrelse på svarfelt Stort

- Sideskift Kopier Slett

- Avkrysningsbokser – flere svar per spørsmål Innstillinger Minimer Kopier Slett

11. Intratester reliability

Please select the measure of intratester reliability you believe can be used to obtain accurate estimates of voluntary activation:

☐ Obligatorisk spørsmål

Svaralternativer Rediger som tekst | **Forvalgt**

- Coefficient of variation (CV). ☐ Sl
- Intraclass correlation coefficients (ICC). ☐ Sl
- Pearson r. ☐ Sl
- Standard error of measurement (SEM). ☐ Sl
- Other. ☐ Sl

Legg til svaralternativ

- Maks antall svaralternativer

Spørsmål – fritekstsvar Innstillinger Minimer Kopier Slett

11.1 Intratester reliability - threshold value

For each of the selected intratester reliability measurements in Question 11, please provide the threshold value you believe is appropriate (e.g., <5%) to obtain accurate estimates of voluntary activation.

Please also provide additional comments here as necessary:

☐ Obligatorisk spørsmål

Størrelse på svarfelt Stort

Spørsmål – fritekstsvar Innstillinger Minimer Kopier Slett

12. Determination of maximal stimulation intensity

A ‘twitch ramp procedure’ is often utilised to determine the optimal stimulus intensity during voluntary contractions. The maximal stimulus intensity is then further increased (e.g., an additional +20%) to ensure all motoneurons are recruited by the stimulus.
Describe your ‘twitch ramp procedure’: what is the stimulus intensity increase for each step of the ramp? What rule do you use to judge that you have reached maximal stimulus intensity? What additional percentage increase would you apply to ensure a ‘supramaximal stimulation’?
For example: ‘the current is increased by 20mA at each step, maximal stimulus intensity is determined when there is no further increase in twitch force amplitude for 5 successive increases in current, the supramaximal stimulus intensity is determined by adding 20% to the maximal stimulus intensity’.

☐ Obligatorisk spørsmål

Størrelse på svarfelt Stort

Spørsmål – fritekstsvar Innstillinger Minimer Kopier Slett

12.1 Determination of maximal stimulation intensity - current increase

State the current increase (e.g., +20mA per step) that you use:

☐ Obligatorisk spørsmål

Størrelse på svarfelt Lite

Spørsmål – fritekstsvar Innstillinger Minimer Kopier Slett

12.1 Determination of maximal stimulation intensity - stopping rule

Indicate whether your stopping rule involves the M-wave peak-to-peak amplitude, twitch amplitude or both (e.g., no increase in twitch force and/or M-wave amplitude for 5 successive increases in current):

☐ Obligatorisk spørsmål

Størrelse på svarfelt Stort

- Spørsmål – fritekstsvar Innstillinger Minimer Kopier Slett

12.3 Determination of maximal stimulation intensity - supramaximal

State the supramaximal stimulation intensity (e.g., +20% on stimulation intensity eliciting a maximum M-wave or peak twitch):

☐ Obligatorisk spørsmål

Størrelse på svarfelt Lite

- Spørsmål – fritekstsvar Innstillinger Minimer Kopier Slett

13. Benchmark of VA values

A goal of many studies assessing voluntary activation is to determine if the activation capacity of a muscle group is improved following a training program or reduced with fatigue or when sustaining an acute injury or a chronic pathology. Studies will consequently make comparisons with a control group/condition where activation is considered ‘maximal’.

What level of percentage activation capacity would you consider to be 'maximal' (eg, ≥ 90%)?
Additionally, please describe if the level you consider to be 'maximal' differs between muscle groups:

☐ Obligatorisk spørsmål

Størrelse på svarfelt Stort

- Spørsmål – fritekstsvar Innstillinger Minimer Kopier Slett

14. Description of a familiarisation session

To obtain an accurate estimate of voluntary activation, participants must be accustomed to the neuromuscular assessment procedure.
Describe what you would include in familiarization sessions (eg, do you include submaximal and maximal voluntary contractions with and without stimulation (s), and if so, how many contractions? Do you expose participants to stimulation? Etc.)

☐ Obligatorisk spørsmål

Størrelse på svarfelt Stort

- Sideskift Kopier Slett

Radioknapper – kun ett svar per spørsmål | Innstillinger | Minimer | Kopier | Slett

15. Familiarisation

How many familiarization sessions do you believe are required before participants can produce reliable measures (as per your specific definition of reliable) or voluntary activation?
Note: Some studies may have specific testing requirements (eg, testing certain muscles or populations may require more, or less, testing sessions). Answer in relation to healthy populations and major / commonly tested muscle groups.

☐ Obligatorisk spørsmål

Svaralternativer (Rediger som tekst)	Forvalgt	
1 session.	○	Sl
2 sessions.	○	Sl
More than 2 sessions.	○	Sl

Legg til svaralternativ

Spørsmål – fritekstsvar | Innstillinger | Minimer | Kopier | Slett

15.1 Familiarisation

If there is any additional information you would like to provide regarding familiarization procedures, please write it in the box below (eg, if you think reliability differs between muscle groups, or any other notable points).
If you indicated that one familiarization session is appropriate, please indicate if you believe familiarization and experimental testing can be conducted on the same day (eg, participant constraints mean they may not be able to attend the lab on another occasion), or whether familiarization and formal testing must occur on different days:

☐ Obligatorisk spørsmål

Størrelse på svarfelt: Stort

Avkrysningsbokser – flere svar per spørsmål | Innstillinger | Minimer | Kopier | Slett

16. Level of acceptable reliability for familiarisation

What do you define as a required level of within-session reliability for voluntary activation assessment during a familiarization session? Select any measures of reliability that you use:

☐ Obligatorisk spørsmål

Svaralternativer (Rediger som tekst)	Forvalgt	
Coefficient of variation (CV).	☐	Sl
Intraclass correlation coefficients (ICC).	☐	Sl
Pearson r.	☐	Sl
Standard error of measurement (SEM).	☐	Sl
Other.	☐	Sl

Legg til svaralternativ

- Maks antall svaralternativer

Spørsmål – fritekstsvar | Innstillinger | Minimer | Kopier | Slett

16.1 Intratester reliability - threshold value

For each of the selected within-session reliability measures you selected in Question 16, please provide the threshold value (eg, CV <5%, ICC> 0.80 etc) that you use:

Please also provide additional comments here as necessary:

☐ Obligatorisk spørsmål

Størrelse på svarfelt Stort

Radioknapper – kun ett svar per spørsmål Innstillinger Minimer Kopier Slett

17. Number of contractions completed

How many contractions should be collected during an experimental session to obtain a *valid* and *reliable* estimate of voluntary activation? Please answer for situations without time constraints during the testing session (eg baseline testing or post-intervention testing); not situations where the effects of an exercise session may dissipate quickly (eg, investigations of fatigue).
Note: multiple contractions are normally recorded but some may be unsuitable for inclusion in analysis (for instance, they may be sub-maximal). Therefore, indicate the total number of contractions that should be collected during experimental sessions, to account for any contractions that may be discarded during analysis.

☐ Obligatorisk spørsmål

**Svaralternativer** Rediger som tekst	**Forvalgt**	
1 contraction.	○	Sl
2 contractions.	○	Sl
3 contractions.	○	Sl
4 contractions	○	Sl
5 contractions.	○	Sl
More than 5 contractions.	○	Sl
Other - specific criterion (please describe in text box below).	○	Sl

Legg til svaralternativ

Spørsmål – fritekstsvar Innstillinger Minimer Kopier Slett

Visning er avhengig av svar på tidligere spørsmål.

17.1 Number of contractions completed - other

If you use a specific criterion to determine the number of contractions (eg, CV <3% between successive contractions) within an experimental session, please describe your criterion here:

☐ Obligatorisk spørsmål

Størrelse på svarfelt Stort

Spørsmål – fritekstsvar Innstillinger Minimer Kopier Slett

18. Instructions provided to participants

The instructions provided to participants can affect the maximum force generated during a voluntary contraction. For example, participants may be instructed to contract *as* ***hard*** *as possible* or *as* ***fast*** *as possible* but may have difficulty achieving *both* simultaneously).
Describe the instructions, including reference to any external feedback, that you provide to participants when assessing voluntary activation (eg, "I would like you to contract as hard as possible, make this trace on the screen go as high as you can"):

☐ Obligatorisk spørsmål

Størrelse på svarfelt Stort

- Radioknapper – kun ett svar per spørsmål   Innstillinger   Minimer   Kopier   Slett

19. Provision of feedback

Rate the importance of providing visual (eg, force or surface electromyography traces) and / or verbal (actual peak values) feedback to participants during maximal voluntary contractions where electrical stimulation is applied, to achieve a valid and reliable measurement:

☐ Obligatorisk spørsmål

Svaralternativer Rediger som tekst	Forvalgt	
Unnecessary.	○	Sl
Somewhat unnecessary.	○	Sl
Neutral.	○	Sl
Somewhat essential.	○	Sl
Essential.	○	Sl

Legg til svaralternativ

- Radioknapper – kun ett svar per spørsmål   Innstillinger   Minimer   Kopier   Slett

19.1 Provision of feedback - timing

If you do provide feedback to participants, is the feedback provided during the contraction (real time feedback) or after the contraction (during rest periods)?

☐ Obligatorisk spørsmål

Svaralternativer Rediger som tekst	Forvalgt	
Real time feedback.	○	Sl
Feedback during rest periods.	○	Sl
Other (please describe in text box below).	○	Sl

Legg til svaralternativ

- Spørsmål – fritekstsvar   Innstillinger   Minimer   Kopier   Slett

Visning er avhengig av svar på tidligere spørsmål.

19.1.1 Provision of feedback - timing (other)

Please detail when feedback is provided to participants:

☐ Obligatorisk spørsmål

Størrelse på svarfelt Stort

- Radioknapper – kun ett svar per spørsmål   Innstillinger   Minimer   Kopier   Slett

20. Number of contractions used for analysis

When calculating voluntary activation, do you believe it is more appropriate to use several contractions and calculate an average, or a single contraction where the highest value was recorded?

☐ Obligatorisk spørsmål

**Svaralternativer** Rediger som tekst	Forvalgt	
Average of several contractions.	○	Sl
Single contraction (peak value).	○	Sl
Other (please describe in text box below).	○	Sl

Legg til svaralternativ

- Spørsmål – fritekstsvar   Innstillinger   Minimer   Kopier   Slett

Visning er avhengig av svar på tidligere spørsmål.

20.1. Number of contractions used for analysis - other

Please describe:

☐ Obligatorisk spørsmål

Størrelse på svarfelt Stort

- Radioknapper – kun ett svar per spørsmål   Innstillinger   Minimer   Kopier   Slett

Visning er avhengig av svar på tidligere spørsmål.

20.2 Number of contractions used for analysis - average

If you use several contractions to calculate an average value for voluntary activation, please indicate how many contractions should be included in the average value:

☐ Obligatorisk spørsmål

**Svaralternativer** Rediger som tekst	Forvalgt	
2 contractions.	○	Sl
3 contractions.	○	Sl
4 contractions.	○	Sl
5 contractions.	○	Sl
More than 5 contractions.	○	Sl

Legg til svaralternativ

- Radioknapper – kun ett svar per spørsmål   Innstillinger   Minimer   Kopier   Slett

20.3 Number of contractions used for analysis - fatigue

In studies where studying the effect of fatigue is the aim, the effects may only be observable immediately following fatiguing exercise. In studies assessing

the effect of fatigue, do you believe it is more appropriate to use several contractions to calculate an average value for voluntary activation, or a single contraction post exercise?

Obligatorisk spørsmål

Svaralternativer Rediger som tekst	Forvalgt	
Average of several contractions.	○	Sl
Single contraction.	○	Sl

Legg til svaralternativ

- Sideskift — Kopier Slett

- Radioknapper – kun ett svar per spørsmål — Innstillinger — Minimer Kopier Slett

21. VA calculation

Please select the formula below that you use to calculate voluntary activation (VA).
Note: D = difference between maximum voluntary force and the force immediately before the electrical stimulus was triggered; superimposed twitch = the difference between maximum voluntary force and force elicited by the electrical stimulus.

Obligatorisk spørsmål

Svaralternativer Rediger som tekst	Forvalgt	
100 – D × (Maximum evoked force ÷ maximum voluntary force) ÷ resti	○	Sl
(1 – superimposed twitch ÷ resting twitch amplitude) × 100	○	Sl
Other (please describe in text box below).	○	Sl

Legg til svaralternativ

- Spørsmål – fritekstsvar — Innstillinger — Minimer Kopier Slett

Visning er avhengig av svar på tidligere spørsmål.

21.1. VA calculation - other

If you use a different formula to the options in Question 21, please describe it here:

Obligatorisk spørsmål

Størrelse på svarfelt Lite

- Radioknapper – kun ett svar per spørsmål — Innstillinger — Minimer Kopier Slett

22. Dynamic vs. isometric contractions

Electrical stimulation is typically applied during isometric contractions. Do you believe the electrical stimulation technique is suitable for use during dynamic contractions (ie, can it provide a valid and reliable estimate of voluntary activation during dynamic contractions on an isokinetic dynamometer)? Please rate your belief using options below:

Obligatorisk spørsmål

Svaralternativer Rediger som tekst — Forvalgt

Cannot be used during dynamic contractions. _ SI

May be suitable in particular instances, during dynamic contractions. _ SI

Can be used during dynamic contractions. _ SI

Legg til svaralternativ

- Spørsmål – fritekstsvar Innstillinger Minimer Kopier Slett

23. Application of stimulus

Describe the optimal method to trigger an electrical stimulus during a maximal voluntary contraction (eg, the stimulus is triggered automatically when voluntary force reaches a specific percentage of maximum for a minimum given period of time).
If using an automatic trigger, describe how you decide to manually trigger the stimulus if the conditions for the automatic trigger are not met (eg, "if the condition of max voluntary force (± 3%) is not maintained for> 300ms, I will manually trigger the stimulus if the participant has reached an obvious plateau. If they have not reached an obvious plateau, contraction is repeated ").

☐ Obligatorisk spørsmål

Størrelse på svarfelt Stort

- Radioknapper – kun ett svar per spørsmål Innstillinger Minimer Kopier Slett

24. Twitch interpolation vs. central activation ratio (CAR)

The twitch interpolation technique involves the application of a potentiated resting twitch following a maximal voluntary contraction. This is used for comparison with the increment in force following stimulation during voluntary contraction.
Alternatively, the central activation ratio (CAR) compares maximal voluntary force with the maximum force elicited by electrical stimulation during a maximal voluntary contraction.
Please indicate which method you believe provides a more valid estimate of voluntary activation:

☐ Obligatorisk spørsmål

Svaralternativer Rediger som tekst **Forvalgt**

CAR provides a more valid estimate. _ SI

Both methods provide a valid estimate. _ SI

Twitch interpolation provides a more valid estimate. _ SI

Legg til svaralternativ

- Radioknapper – kun ett svar per spørsmål Innstillinger Minimer Kopier Slett

25. Rest between contractions - no time constraint

Indicate the duration of rest required between maximal voluntary contractions, when the aim of the protocol is to assess responses to training, or capacity in specific populations / in previously injured participants (no apparent time constrain t).

☐ Obligatorisk spørsmål

Svaralternativer Rediger som tekst **Forvalgt**

No rest. _ SI

0-30 seconds rest. _ SI

30-60 seconds rest. _ ○ Sl

60-120 seconds rest. _ ○ Sl

More than 120 seconds rest. _ ○ Sl

Legg til svaralternativ

- Radioknapper – kun ett svar per spørsmål   Innstillinger   Minimer   Kopier   Slett

25.1 Rest between contractions - fatigue

Please indicate the duration of rest required between maximal voluntary contractions when the aim of the study is to assess the effect of fatigue when there is a time constraint (ie, a partial recovery of neuromuscular function may occur with longer rest periods):

☐ Obligatorisk spørsmål

Svaralternativer Rediger som tekst **Forvalgt**

No rest. _ ○ Sl

0-30 seconds rest. _ ○ Sl

30-60 seconds rest. _ ○ Sl

60-120 seconds rest. _ ○ Sl

More than 120 seconds rest. _ ○ Sl

Legg til svaralternativ

- Radioknapper – kun ett svar per spørsmål   Innstillinger   Minimer   Kopier   Slett

26. Evoked voluntary force relationship

Some research studies estimate the 'true maximum force' by plotting force against the superimposed twitch size (recorded during submaximal contraction intensities) and extrapolating this relationship.

Alternatively, other studies estimate voluntary activation by comparing the superimposed twitch size to that of a potentiated resting twitch.
Indicate which of these methods you believe provides a more valid estimate of voluntary activation:

☐ Obligatorisk spørsmål

Svaralternativer Rediger som tekst **Forvalgt**

Extrapolating the evoked/voluntary force relationship provides a more _ ○ Sl

Neither method offers superior validity in terms of estimating voluntary _ ○ Sl

Comparing superimposed and resting potentiated twitches as a ratio p _ ○ Sl

Legg til svaralternativ

- Radioknapper – kun ett svar per spørsmål   Innstillinger   Minimer   Kopier   Slett

27. Stimulation of antagonists

A concern when using the twitch interpolation technique is the potential stimulation of antagonist muscles during maximal contraction. Indicate if you believe it is possible to selectively stimulate agonist muscles:

☐ Obligatorisk spørsmål

Svaralternativer Rediger som tekst **Forvalgt**

Antagonist stimulation is unavoidable. _ ○ Sl

Stimulation will always result in partial stimulation of antagonist muscl _ ○ Sl

It is possible to selectively stimulate the agonist muscle. _ ○ Sl

Legg til svaralternativ

- Spørsmål – fritekstsvar Innstillinger Minimer Kopier Slett

27.1. Stimulation of antagonists - checking

Please indicate how you check for antagonist stimulation during voluntary contractions:

☐ Obligatorisk spørsmål

Størrelse på svarfelt Stort

- Spørsmål – fritekstsvar Innstillinger Minimer Kopier Slett

27.2. Stimulation of antagonists - recommendations

Please provide any practical recommendations for improving selective agonist stimulation during maximal voluntary contractions:

☐ Obligatorisk spørsmål

Størrelse på svarfelt Stort

- Sideskift Kopier Slett

- Radioknapper – kun ett svar per spørsmål Innstillinger Minimer Kopier Slett

28. Inferences from twitch interpolation

Which of the statements below best summarizes the inferences that can be made when a deficit (ie, fatigue) in percentage voluntary activation is observed?

☐ Obligatorisk spørsmål

Svaralternativer Rediger som tekst **Forvalgt**

The completeness of voluntary activation cannot be determined by ele _ ○ Sl

Lower levels of percentage voluntary activation suggest a participant i _ ○ Sl

Lower levels of percentage voluntary activation suggest a participant i _ ○ Sl

Lower levels of percentage voluntary activation suggestion a participa _ ○ Sl

Other (please describe in text box below). _ ○ Sl

Legg til svaralternativ

- Spørsmål – fritekstsvar Innstillinger Minimer Kopier Slett

28.1 Inferences from twitch interpolation - other

Please detail any other inferences that can be made when a deficit (ie, fatigue) in percentage voluntary activation is observed:

☐ Obligatorisk spørsmål

Størrelse på svarfelt Stort

▪ Matrise – ett svar per spørsmål Innstillinger Minimer Kopier Slett

29.1. Methodological limitations - stimulation parameters

Rate the effect that each stimulation parameter may have on validity. *Note: please select your rating assuming a twin / doublet stimulation method.*

Rader	Obligatorisk	
It is difficult to confine stimulation to the agonist muscle (a portion of ant	☐	Slett
Maintenance of constant and optimal contact between the stimulating pe	☐	Slett

Legg til rad

Kolonner Rediger som tekst	Forvalgt	
1. Completely limits the validity of the method.	○	Sl
2. Majorly limits the validity.	○	Sl
3. Moderate effect on validity.	○	Sl
4. A minor effect on validity.	○	Sl
5. Has no influence on validity.	○	Sl

Legg til kolonne

▪ Matrise – ett svar per spørsmål Innstillinger Minimer Kopier Slett

29.2. Methodological limitations - participant factors

Rate the effect that each participant related factor may have on validity. *Note: please select your rating assuming a twin / doublet stimulation method.*

Rader	Obligatorisk	
During investigations, it is difficult to ensure participants are contracting	☐	Slett
The measure may be dependent on the muscle group being assessed (	☐	Slett

Legg til rad

Kolonner Rediger som tekst	Forvalgt	
1. Completely limits the validity of the method.	○	Sl
2. Majorly limits the validity.	○	Sl
3. Moderate effect on validity.	○	Sl
4. A minor effect on validity.	○	Sl
5. Has no influence on validity.	○	Sl

Legg til kolonne

▪ Matrise – ett svar per spørsmål Innstillinger Minimer Kopier Slett

29.3. Methodological limitations - internal validity

Rate the effect that each proposed factor may have on the internal validity of the method. *Note: please select your rating assuming a twin / doublet stimulation method.*

Rader	Obligatorisk	
The size of the superimposed twitch is limited by antidromic collisions at	☐	Slett
The method is insensitive to small changes in activation at high levels o	☐	Slett

Legg til rad

Kolonner Rediger som tekst	Forvalgt	
1. Completely limits the validity of the method.	○	Sl
2. Majorly limits the validity.	○	Sl
3. Moderate effect on validity.	○	Sl
4. A minor effect on validity.	○	Sl
5. Has no influence on validity.	○	Sl

Legg til kolonne

▪ Matrise – ett svar per spørsmål Innstillinger Minimer Kopier Slett

29.4. Methodological limitations - ecological validity

Rate the effect that each proposed factor may have on the ecological validity of the method. *Note: please select your rating assuming a twin / doublet stimulation method.*

Rader	Obligatorisk	
Deficits observed in lab-based settings (e.g., often isometric, single joint	☐	Slett

Legg til rad

Kolonner Rediger som tekst	Forvalgt	
1. Completely limits the validity of the method.	○	Sl
2. Majorly limits the validity.	○	Sl
3. Moderate effect on validity.	○	Sl
4. A minor effect on validity.	○	Sl
5. Has no influence on validity.	○	Sl

Legg til kolonne

▪ Spørsmål – fritekstsvar Innstillinger Minimer Kopier Slett

29.5. Methodological limitations - other

If there are other factors you believe limit the technique, but are not listed in Questions 29.1-29.4, please highlight them here, and indicate the strength of an effect they may have on validity.

☐ Obligatorisk spørsmål

Størrelse på svarfelt Stort

Avkrysningsbokser – flere svar per spørsmål | Innstillinger | Minimer | Kopier | Slett

30. Populations where the measure provides a meaningful assessment of voluntary act

For which athlete/patient (sport/clinical) subpopulations can twitch interpolation provide a useful measure of voluntary activation? Please indicate by ticking the appropriate box:

☐ Obligatorisk spørsmål

Svaralternativer Rediger som tekst	Forvalgt	
Highly resistance trained athletes.	☐	Sl
Highly aerobically trained athletes.	☐	Sl
Healthy younger (under 60 years old) participants.	☐	Sl
Healthy older (60 years or older) participants.	☐	Sl
Stroke patients.	☐	Sl
Cerebral Palsy.	☐	Sl
Other (please describe in text box below).	☐	Sl

Legg til svaralternativ

- Maks antall svaralternativer

Spørsmål – fritekstsvar | Innstillinger | Minimer | Kopier | Slett

Visning er avhengig av svar på tidligere spørsmål.

30.1. Populations - other

Which other subpopulations can twitch interpolation provide a useful measure of voluntary activation? Please describe here:

☐ Obligatorisk spørsmål

Størrelse på svarfelt: Stort

Avkrysningsbokser – flere svar per spørsmål | Innstillinger | Minimer | Kopier | Slett

31. Settings where twitch interpolation provides a useful assessment of voluntary activati

Indicate in which settings twitch interpolation can provide a useful measure of voluntary activation (you may select more than one):

☐ Obligatorisk spørsmål

Svaralternativer Rediger som tekst	Forvalgt	
Post-injury (e.g., muscle strain, tendinopathy).	☐	Sl
Following surgery (e.g., hip replacement, ACL reconstruction).	☐	Sl
Following a training intervention (e.g., resistance training).	☐	Sl
To determine the effects of fatigue induced by exhaustive exercise (e.(	☐	Sl
To determine the effects of ergogenic aids (e.g., caffeine).	☐	Sl
Other (please describe in text box below).	☐	Sl

Legg til svaralternativ

- Maks antall svaralternativer

- Spørsmål – fritekstsvar Innstillinger Minimer Kopier Slett

31.1. Settings - further comments

If there are other settings where twitch interpolation can provide a useful measure of voluntary activation, please describe here. Please also provide any additional comments that you may have regarding the options you selected in Question 31:

☐ Obligatorisk spørsmål

Størrelse på svarfelt Stort

- Spørsmål – fritekstsvar Innstillinger Minimer Kopier Slett

32. When should the application of electrical nerve/muscle stimulations for voluntary ac

Describe any conditions/settings where you believe assessing voluntary activation is contraindicated or does not provide a valid assessment of voluntary activation:

☐ Obligatorisk spørsmål

Størrelse på svarfelt Stort

- Tekst Innstillinger Minimer Kopier Slett

33. Analysis of voluntary force
The following four questions will ask you to describe the methods you use for analysis of voluntary force traces.

- Spørsmål – fritekstsvar Innstillinger Minimer Kopier Slett

33.1. Analysis of voluntary force - software

Please detail the software used (both for recording and analysis) during your analysis of voluntary force traces.

☐ Obligatorisk spørsmål

Størrelse på svarfelt Stort

- Spørsmål – fritekstsvar Innstillinger Minimer Kopier Slett

33.2. Analysis of voluntary force - method

Please describe if you use manual or automatic methods to determine peak voluntary force.

☐ Mandatory question

Size of response field Large

- Questions - free text answers Settings Minimize Copy Delete

33.3. Analysis of voluntary force - peak

Describe if you consider peak voluntary force as a single discrete point, or an average over a time period (if so, please specify the time period when averaging).

☐ Mandatory question

Size of response field Large

- Questions - free text answers Settings Minimize Copy Delete

33.4. Analysis of voluntary force - differences

Briefly mention any advantages / disadvantages of manual and automatic methods for determining peak force (eg, automatic methods save time, but may result in an erroneous identification of peak force):

☐ Mandatory question

Size of response field Large
