# Supplementary material for: Neuromuscular electrical stimulation during maximal voluntary contraction: a Delphi survey with expert consensus
Source: Eur J Appl Physiol. 2023 May 29;123(10):2203–12. doi: 10.1007/s00421-023-05232-1 (PMC10492693; doi:10.1007/s00421-023-05232-1)
Supplement: Supplementary file 2 — Supplementary file2 (PDF 884 KB) [file 421_2023_5232_MOESM2_ESM.pdf]

Sideelementer

Mellomtittel

Tekst

Bilde

Sideskift

Spørsmål

Med tekstsvar

Med radioknapper

Med avkrysnings-bokser

Med nedtrekksliste

Med lineær skala

Matrisespørsmål

Med radioknapper

Med avkrysnings-bokser

Minimer alle Maksimer alle Avbryt Lagre Lagre og vis

Tekst Minimer Kopier Slett

"Twitch interpolation for the assessment of voluntary activation: a Delphi study and methodological consensus"

Purpose of the project

The ability to maximally contract a muscle is dependent on conscious and deliberate voluntary activation (VA) and recruitment of motor neurons via the central nervous system (CNS). However, the specific methodologies used to determine VA using the interpolated twitch technique (ITT) vary across different studies and research groups.

This Delphi study will aim to provide an expert consensus on the appropriate definition, technique and methodology that should be utilised when completing ITT assessments of muscle VA.

Who is responsible for the research project?

John Owen Osborne from UiT The Arctic University of Norway is the project leader responsible for this project. Co-investigators on this project include: Robert Buhmann (University of the Sunshine Coast), Olivier Girard (University of Western Australia), Paul Marshall (University of Auckland), Dawson Kidgell (Monash University) and Jamie Tallent (University of Essex).

Why are you being asked to participate?

You are being asked to participate as you have been identified as an expert in neuromuscular testing. Specifically, you have demonstrated research experience in this field, as evidenced by multiple publications in peer-review journal within the past two decades (≥3 papers as lead author or ≥10 in another position).

What does participation involve for you?

Participation in this Delphi study will involve completing the following online questionnaire. You will be asked to answer 33 questions about the current methodology you believe is 'best-practice' for assessing voluntary activation, as well as your belief around the validity of different techniques for undertaking twitch interpolation. It is anticipated that the 33 questions of this questionnaire will take ~30 minutes to complete.

The project investigators will then collate and anonymise all answers and resend the aggregate questionnaire responses back to all participating experts. You will be then asked to provide feedback on the updated methodology, and this iterative and anonymous process will continue until there is a majority consensus reached among the expert group.

Participation is voluntary

If you chose to participate, you can withdraw your consent at any time without giving a reason. All information about you will then be made anonymous. There will be no negative consequences for you if you chose not to participate or later decide to withdraw.

Tekst Minimer Kopier Slett

Your personal privacy – how we will store and use your personal data

We will only use your personal data for the purpose(s) specified in this information letter. We will process your personal data confidentially and in accordance with data protection legislation (the General Data Protection Regulation and Personal Data Act).

Only the primary investigator (John Osborne) will have access to your personal data (name, age, sex, job position, institution) and all identifying information will be removed or deidentified via recoding, before the questionnaire responses are sent to coinvestigators for aggregation and processing. The key code and personal data will be stored separately in a password-protected file on UiT's research servers. All data processing of questionnaire responses will be completed using the deidentified answers, and the aggregate responses for each round will be anonymous to other experts. No participant in this study will be recognizable or personally identifiable in any publication from this research.

What will happen to your personal data at the end of the research project?
The project is scheduled to end 31 December 2022. Deidentified data will be irreversibly anonymised on this date when the personal code key is deleted.
Your rights
So long as you can be identified in the collected data, you have the right to:

- access the personal data that is being processed about you
- request that your personal data is deleted
- request that incorrect personal data about you is corrected/rectified
- receive a copy of your personal data (data portability), and
- send a complaint to the Data Protection Officer or The Norwegian Data Protection Authority regarding the processing of your personal data

What gives us the right to process your personal data?
We will process your personal data based on your consent.
Based on an agreement with UiT The Arctic University of Norway, NSD – The Norwegian Centre for Research Data AS has assessed that the processing of personal data in this project is in accordance with data protection legislation.
Where can I find out more?
If you have questions about the project, or want to exercise your rights, contact:

- UiT The Arctic University of Norway via Dr. John Owen Osborne (john.owen.osborne@uit.no)
- UiT Data Protection Officer: Joakim Bakkevold, mailadr: personvernombud@uit.no tlf. 77646322, 97691578.
- NSD – The Norwegian Centre for Research Data AS, by email: (personverntjenester@nsd.no) or by telephone: +47 55 58 21 17.

- Avkrysningsbokser – flere svar per spørsmål | Innstillinger | Minimer | Kopier | Slett

Consent to participate

By selecting the box below, you are indicating that you:

- Have read and understood the research project information.
- Have had any questions answered to your satisfaction.
- Understand that you are free to withdraw at any time.
- Consent to undertaking the study as per the procedures outlined in the participant information sheet.
- Understand that non-identifiable data from this study may be used in similar, relate[illegible] projects in the future.

If you do not consent to participate in the project, you can not complete this questionnaire.

☑ Obligatorisk spørsmål

Svaralternativer | Rediger som tekst | Forvalgt

I consent to participate in this project. ☐ Sl

Legg til svaralternativ

- Maks antall svaralternativer

- Sideskift | Kopier | Slett

- Fullt navn | Innstillinger | Minimer | Slett

For innloggede brukere fylles navnet ut automatisk

Your name (first name and surname)?

This information will only be used by the primary investigator to identify the participating respondents against the initial recruitment email list of experts.

☑ Obligatorisk spørsmål

- Sideskift   Kopier   Slett

- Tekst   Innstillinger   Minimer   Kopier   Slett

Note:
The purpose of this second round of the questionnaire is to further develop an expert consensus on the most appropriate methods for electrical stimulation of peripheral nerve / muscle. Please answer all questions in the context of stimulation during a maximum voluntary contraction (and not in the context of H-reflex or conditioned responses (paired pulse) assessments).

- Radioknapper – kun ett svar per spørsmål   Innstillinger   Minimer   Kopier   Slett

1. Term used to describe the outcome measure.

There are several names given to the outcome variable obtained from the **electrical stimulation** of **muscle / peripheral nerve** during and after maximal voluntary contraction. Indicate the name of the variable you believe is most appropriate:

☐ Obligatorisk spørsmål

Svaralternativer Rediger som tekst		Forvalgt	
Voluntary activation.	_	○	Sl
Voluntary activation level.	_	○	Sl

Legg til svaralternativ

- Radioknapper – kun ett svar per spørsmål   Innstillinger   Minimer   Kopier   Slett

2. Outcome measure definition

There are several definitions of the outcome variable obtained from electrical stimulation of muscle / nerve during maximum voluntary contraction. Select the most appropriate definition of this variable:

☐ Obligatorisk spørsmål

Svaralternativer Rediger som tekst		Forvalgt	
The amount of force voluntarily produced during contraction as a prop	_	○	Sl
The level of inactivation during a maximum voluntary contraction.	_	○	Sl

Legg til svaralternativ

- Radioknapper – kun ett svar per spørsmål   Innstillinger   Minimer   Kopier   Slett

4.1. Stimulation location validity

Assessment of voluntary activation of a muscle group uses electrical stimulation of either the peripheral nerve or muscle belly. Select an answer that best describes your belief of voluntary activation assessment validity using muscle and / or nerve stimulation.

Note: as stimulation of a peripheral nerve innervating the muscle of interest may not always be possible, or may be difficult (eg, sciatic nerve), please answer for instances where nerve or muscle stimulation *are* plausible.

Obligatorisk spørsmål

Svaralternativer Rediger som tekst **Forvalgt**

- Both muscle and nerve stimulation can provide a valid assessment of ◯ Sl
- Nerve stimulation has a higher validity than muscle stimulation. ◯ Sl

Legg til svaralternativ

Sideskift Kopier Slett

Nedtrekksliste – kun ett svar per spørsmål Innstillinger Minimer Kopier Slett

6. Pulse width

Stimulation pulse widths typically range from 0.1 to 1ms. A wider pulse width increases the number of motoneurons recruited. However, increasing pulse widths may also increase the pain / discomfort experienced by participants. Indicate the *minimum* pulse width you deem appropriate to assess activation capacity.

Obligatorisk spørsmål

Svaralternativer Rediger som tekst **Forvalgt**

- 0.1-0.2 ms. ◯ Sl
- 0.4-0.5 ms. ◯ Sl
- 1.0 ms. ◯ Sl

Legg til svaralternativ

Radioknapper – kun ett svar per spørsmål Innstillinger Minimer Kopier Slett

7. Number of stimuli

When applying an electrical stimulus during a voluntary contraction, investigators may apply single or multiple stimuli. Indicate the minimum number of stimuli you believe is optimal for a valid assessment of voluntary activation of muscle.

Obligatorisk spørsmål

Svaralternativer Rediger som tekst **Forvalgt**

- 1 stimuli. ◯ Sl
- 2 stimuli (doublets). ◯ Sl

Legg til svaralternativ

Radioknapper – kun ett svar per spørsmål Innstillinger Minimer Kopier Slett

8. Stimulation source

Stimulating electrodes or a stimulating pen may be used to elicit an electrical muscle / nerve stimulus. Which of these methods do you believe is most effective (ie, results in the most valid and reliable measures) for stimulating muscle / nerve?

Obligatorisk spørsmål

Svaralternativer Rediger som tekst **Forvalgt**

Stimulating electrodes provide the best electrical stimulus for muscle/r _ Sl

Both stimulating methods are equally effective in eliciting an electrical _ Sl

Legg til svaralternativ

Matrise – ett svar per spørsmål | Innstillinger | Minimer | Kopier | Slett

9.1. Electrode size and type - cathode

Electrode size can affect the pain / discomfort experienced by participants following stimulation. While larger electrodes can reduce the pain / discomfort, using electrodes that are too big can also result in stimulation of unwanted muscles.
Please select the most appropriate **cathode** size for stimulation during maximal voluntary contractions for each target nerve / muscle group:

Rader	Obligatorisk	
Femoral nerve/quadriceps.	☐	Slett
Tibial nerve/plantar flexors.	☐	Slett
Common peroneal nerve/dorsiflexors.	☐	Slett

Legg til rad

Kolonner Rediger som tekst	Forvalgt	
Stimulating pen.	○	Sl
Small (3.2 or 5 cm (round); 3 x 5 cm (width x length)).	○	Sl
Medium (5 x 5 cm; 5 x 9 cm (width x length)).	○	Sl
Large (5 x 13 cm; 7.5 x 10 cm; 7.5 x 13 cm (width x length)).	○	Sl

Legg til kolonne

Matrise – ett svar per spørsmål | Innstillinger | Minimer | Kopier | Slett

9.2. Electrode size and type - anode

Electrode size can affect the pain / discomfort experienced by participants following stimulation. While larger electrodes can reduce the pain / discomfort, using electrodes that are too big can also result in stimulation of unwanted muscles.
Please select the most appropriate **anode** size for stimulation during maximal voluntary contractions for each target nerve / muscle group:

Rader	Obligatorisk	
Femoral nerve/quadriceps.	☐	Slett
Tibial nerve/plantar flexors.	☐	Slett
Common peroneal nerve/dorsiflexors.	☐	Slett

Legg til rad

Kolonner Rediger som tekst	Forvalgt	
Stimulating pen.	○	Sl
Small (3.2 or 5 cm (round); 3 x 5 cm (width x length)).	○	Sl
Medium (5 x 5 cm; 5 x 9 cm (width x length)).	○	Sl
Large (5 x 13 cm; 7.5 x 10 cm; 7.5 x 13 cm (width x length)).	○	Sl

Legg til kolonne

- Matrise – ett svar per spørsmål Innstillinger Minimer Kopier Slett

10.1 (rev2). Rate strategies below from most, to least, useful for reducing the pain experi

Beskrivelse

Rader	Obligatorisk	
Decreasing the pulse width.	☐	Slett
Reducing pulse duration (width) and simulating with a higher current	☐	Slett
Increasing pulse duration (width) and stimulating with a lower current	☐	Slett
Familiarising participants with stimulation in separate familiarisation ses	☐	Slett
Encouraging maximal contractions	☐	Slett

Legg til rad

Kolonner Rediger som tekst	Forvalgt	
1. Most effective	○	Sl
2. Somewhat effective	○	Sl
3. Moderately effective	○	Sl
4. Not very effective	○	Sl
5. Least effective	○	Sl

Legg til kolonne

- Avkrysningsbokser – flere svar per spørsmål Innstillinger Minimer Kopier Slett

10.2 (rev2). Please select any of the options below that you would recommend to reduce

You can select more than one option.

☐ Obligatorisk spørsmål

Svaralternativer Rediger som tekst	Forvalgt	
Decreasing the pulse width.	☐	Sl
Reducing pulse duration (width) and simulating with a higher current	☐	Sl
Increasing pulse duration (width) and stimulating with a lower current	☐	Sl
Familiarising participants with stimulation in separate familiarisation se	☐	Sl
Encouraging maximal contractions	☐	Sl

Legg til svaralternativ

- Maks antall svaralternativer

- Sideskift Kopier Slett

- Tekst Innstillinger Minimer Kopier Slett

11.0 Intra-tester reliability
The following questions (11.1 - 11.3.1) are interested in your beliefs regarding the intratester reliability to obtain accurate estimates of voluntary activation:

- Nedtrekksliste – kun ett svar per spørsmål | Innstillinger | Minimer | Kopier | Slett

11.1 Intratester reliability - 1

Please select your **most preferred** measure of intratester reliability:

☑ Obligatorisk spørsmål

**Svaralternativer** Rediger som tekst	**Forvalgt**	
Coefficient of variation (CV).	○	Sl
Intraclass correlation coefficients (ICC).	○	Sl
Standard error of measurement (SEM).	○	Sl

Legg til svaralternativ

- Spørsmål – fritekstsvar | Innstillinger | Minimer | Kopier | Slett

Visning er avhengig av svar på tidligere spørsmål.

11.1.1 Intratester reliability - threshold value 1

For the intratester reliability selected above for Question 11.1, please provide the threshold value you believe is appropriate (eg, <5%) to obtain accurate estimates of voluntary activation.

☐ Obligatorisk spørsmål

Størrelse på svarfelt Lite

- Nedtrekksliste – kun ett svar per spørsmål | Innstillinger | Minimer | Kopier | Slett

11.2 Intratester reliability - 2

Please select your **second most preferred** measure of intratester reliability:

☑ Obligatorisk spørsmål

**Svaralternativer** Rediger som tekst	**Forvalgt**	
Coefficient of variation (CV).	○	Sl
Intraclass correlation coefficients (ICC).	○	Sl
Standard error of measurement (SEM).	○	Sl

Legg til svaralternativ

- Spørsmål – fritekstsvar | Innstillinger | Minimer | Kopier | Slett

11.2.1 Intratester reliability - threshold value 2

For the **second most preferred** intratester reliability selected for Question 11.2, please provide the threshold value you believe is appropriate (eg, <5%) to obtain accurate estimates of voluntary activation.

☐ Obligatorisk spørsmål

Størrelse på svarfelt Lite

- Nedtrekksliste – kun ett svar per spørsmål   Innstillinger   Minimer   Kopier   Slett

11.3 Intratester reliability - 3

Please select your **least** preferred measure of intratester reliability:

☑ Obligatorisk spørsmål

Svaralternativer Rediger som tekst **Forvalgt**

Svaralternativer	Forvalgt	
Coefficient of variation (CV).	○	Sl
Intraclass correlation coefficients (ICC).	○	Sl
Standard error of measurement (SEM).	○	Sl

Legg til svaralternativ

- Spørsmål – fritekstsvar   Innstillinger   Minimer   Kopier   Slett

11.3.1 Intratester reliability - threshold value 3

For the **least preferred** intratester reliability selected for Question 11.3, please provide the threshold value you believe is appropriate (eg, <5%) to obtain accurate estimates of voluntary activation.

☐ Obligatorisk spørsmål

Størrelse på svarfelt Lite

- Sideskift   Kopier   Slett

- Radioknapper – kun ett svar per spørsmål   Innstillinger   Minimer   Kopier   Slett

12.1 (rev2). Which current increase do you believe is most appropriate for the ramp proc

Beskrivelse

☐ Obligatorisk spørsmål

Svaralternativer Rediger som tekst **Forvalgt**

Svaralternativer	Forvalgt	
10mA or less	○	Sl
20mA	○	Sl
30mA	○	Sl
50mA or greater	○	Sl

Legg til svaralternativ

- Radioknapper – kun ett svar per spørsmål [Innstillinger] [Minimer] [Kopier] [Slett]

12.2 (rev2). How much do you increase the maximal stimulus identified during the ramp |

Assuming there are no special participant considerations (eg obesity) and when the experiment does not involve investigating the effects of fatigue).

☐ Obligatorisk spørsmål

**Svaralternativer** Rediger som tekst		**Forvalgt**	
10%	_	○	Sl
20%	_	○	Sl
30%	_	○	Sl

Legg til svaralternativ

- Radioknapper – kun ett svar per spørsmål [Innstillinger] [Minimer] [Kopier] [Slett]

13. (rev2). Indicate the level of percentage voluntary activation capacity you believe is 'm

Please answer in relation to lower limb muscles and in patients without special considerations.

☐ Obligatorisk spørsmål

**Svaralternativer** Rediger som tekst		**Forvalgt**	
≥85%	_	○	Sl
≥90%	_	○	Sl
≥95%	_	○	Sl
100%	_	○	Sl

Legg til svaralternativ

- Sideskift [Kopier] [Slett]

- Tekst [Innstillinger] [Minimer] [Kopier] [Slett]

16.0 W ithin-session reliability
The following questions (16.1 - 16.3.1) are interested in your beliefs regarding the within-session reliability necessary to obtain accurate estimates of voluntary activation:

- Nedtrekksliste – kun ett svar per spørsmål [Innstillinger] [Minimer] [Kopier] [Slett]

16.1 Level of acceptable reliability for familiarisation - 1

Please select your most preferred measure of within-session reliability for voluntary activation assessment during a familiarization session:

☑ Obligatorisk spørsmål

Svaralternativer Rediger som tekst **Forvalgt**

Coefficient of variation (CV). Sl

Intraclass correlation coefficients (ICC). Sl

Standard error of measurement (SEM). Sl

Legg til svaralternativ

- Spørsmål – fritekstsvar Innstillinger Minimer Kopier Slett

16.1.1 Level of acceptable reliability for familiarisation - threshold value 1

For the most preferred within-session reliability selected for Question 16.1, please provide the threshold value (eg, CV <5%, ICC> 0.80 etc) that you use:

☐ Obligatorisk spørsmål

Størrelse på svarfelt Lite

- Nedtrekksliste – kun ett svar per spørsmål Innstillinger Minimer Kopier Slett

16.2 Level of acceptable reliability for familiarisation - 2

Please select your second most preferred measure of within-session reliability for voluntary activation assessment during a familiarization session:

☑ Obligatorisk spørsmål

Svaralternativer Rediger som tekst **Forvalgt**

Coefficient of variation (CV). Sl

Intraclass correlation coefficients (ICC). Sl

Standard error of measurement (SEM). Sl

Legg til svaralternativ

- Spørsmål – fritekstsvar Innstillinger Minimer Kopier Slett

16.2.1 Level of acceptable reliability for familiarisation - threshold value 2

For the second most pre ferred within-session reliability selected for Question 16.2, please provide the threshold value (eg, CV <5%, ICC> 0.80 etc) that you use:

☐ Obligatorisk spørsmål

Størrelse på svarfelt Lite

- Nedtrekksliste – kun ett svar per spørsmål Innstillinger Minimer Kopier Slett

16.3 Level of acceptable reliability for familiarisation - 3

Please select your least preferred measure of within-session reliability for voluntary activation assessment during a familiarization session:

☑ Obligatorisk spørsmål

Svaralternativer Rediger som tekst **Forvalgt**

Coefficient of variation (CV). Sl

Intraclass correlation coefficients (ICC). Sl

Standard error of measurement (SEM). Sl

Legg til svaralternativ

Spørsmål – fritekstsvar Innstillinger Minimer Kopier Slett

16.3.1 Level of acceptable reliability for familiarisation - threshold value 3

For the least preferred within-session reliability selected for Question 16.3, please provide the threshold value (eg, CV <5%, ICC> 0.80 etc) that you use:

Obligatorisk spørsmål

Størrelse på svarfelt Lite

Sideskift Kopier Slett

Radioknapper – kun ett svar per spørsmål Innstillinger Minimer Kopier Slett

17. Number of contractions completed

What is the **minimum number** of contractions that should be collected to obtain a *valid* and *reliable* estimate of voluntary activation? Please answer for situations without time constraints during the testing session (eg baseline testing or post-intervention testing); not situations where the effects of an exercise session may dissipate quickly (eg, investigations of fatigue).
Note: multiple contractions are normally recorded but some may be unsuitable for inclusion in analysis (for instance, they may be sub-maximal). Therefore, indicate the total number of contractions that should be collected during experimental sessions, to account for any contractions that may be discarded during analysis.

Obligatorisk spørsmål

Svaralternativer Rediger som tekst **Forvalgt**

At least 1 contraction. Sl

Minimum of 2 contractions. Sl

Minimum of 3 contractions. Sl

Minimum of 4 contractions Sl

Minimum of 5 contractions. Sl

Legg til svaralternativ

Radioknapper – kun ett svar per spørsmål Innstillinger Minimer Kopier Slett

18.1 (rev2). Which of the instructions/feedback described below result in the most consis

Beskrivelse

Obligatorisk spørsmål

Svaralternativer Rediger som tekst **Forvalgt**

1. Verbal encourage (e.g., “when you contract, we want you to contrac _ Sl

2. Using feedback from the torque/force-time trace and instructing par _ Sl

Legg til svaralternativ

- Radioknapper – kun ett svar per spørsmål Innstillinger Minimer Kopier Slett

18.2 (rev2). Do you believe that both options described in Question 18.1 are effective?

Beskrivelse

☐ Obligatorisk spørsmål

Svaralternativer Rediger som tekst **Forvalgt**

1. Yes - if my selected option in Q18.1 was not possible to use, the alt _ Sl

2. No - only the answer I selected in Q18.1 was acceptable. _ Sl

Legg til svaralternativ

- Radioknapper – kun ett svar per spørsmål Innstillinger Minimer Kopier Slett

19.1 Provision of feedback - timing

If you do provide feedback to participants, is the feedback provided during the contraction (real time feedback) or after the contraction (during rest periods)?

☐ Obligatorisk spørsmål

Svaralternativer Rediger som tekst **Forvalgt**

Real time feedback. _ Sl

Feedback during rest periods. _ Sl

Legg til svaralternativ

- Sideskift Kopier Slett

- Radioknapper – kun ett svar per spørsmål Innstillinger Minimer Kopier Slett

21. VA calculation

Please select the formula below that you use to calculate voluntary activation (VA).
Note: D = difference between maximum voluntary force and the force immediately before the electrical stimulus was triggered; superimposed twitch = the difference between maximum voluntary force and force elicited by the electrical stimulus.

☐ Obligatorisk spørsmål

Svaralternativer Rediger som tekst **Forvalgt**

100 – D × (Maximum evoked force ÷ maximum voluntary force) ÷ resti _ Sl

(1 – superimposed twitch ÷ resting twitch amplitude) × 100 _ Sl

Legg til svaralternativ

- Radioknapper – kun ett svar per spørsmål   Innstillinger   Minimer   Kopier   Slett

22. Dynamic vs. isometric contractions

Electrical stimulation is typically applied during isometric contractions. Do you believe the electrical stimulation technique is suitable for use during dynamic contractions (ie, can it provide a valid and reliable estimate of voluntary activation during dynamic contractions on an isokinetic dynamometer)? Please rate your belief using options below:

☐ Obligatorisk spørsmål

Svaralternativer Rediger som tekst   **Forvalgt**

Svaralternativ	Forvalgt	
Cannot be used during dynamic contractions.	○	Sl
Can be used during dynamic contractions.	○	Sl

Legg til svaralternativ

- Radioknapper – kun ett svar per spørsmål   Innstillinger   Minimer   Kopier   Slett

23.1 (rev2). Which of the below two triggering methods do you believe results in the mos

Beskrivelse

☐ Obligatorisk spørsmål

Svaralternativer Rediger som tekst   **Forvalgt**

Svaralternativ	Forvalgt	
1. Manually triggering the electrical stimulus once the force/torque trac	○	Sl
2. Automatically triggering the electrical stimulus using pre-set comma	○	Sl

Legg til svaralternativ

- Radioknapper – kun ett svar per spørsmål   Innstillinger   Minimer   Kopier   Slett

23.2 (rev2). Do you believe that both options described in Question 23.1 are effective?

Beskrivelse

☐ Obligatorisk spørsmål

Svaralternativer Rediger som tekst   **Forvalgt**

Svaralternativ	Forvalgt	
1. Yes - if my selected option in Q23.1 was not possible to use, the alt	○	Sl
2. No - only the answer I selected in Q23.1 was acceptable.	○	Sl

Legg til svaralternativ

- Radioknapper – kun ett svar per spørsmål   Innstillinger   Minimer   Kopier   Slett

25.1 Rest between contractions - fatigue

Please indicate the duration of rest required between maximal voluntary contractions when the aim of the study is to assess the effect of fatigue when there <u>is a time constraint</u> (ie, a partial recovery of neuromuscular function may occur with longer rest periods):

☐ Obligatorisk spørsmål

Svaralternativer Rediger som tekst   **Forvalgt**

No rest (0 seconds) SI
1-30 seconds rest SI
More than 30 seconds rest SI

Legg til svaralternativ

Sideskift Kopier Slett

Radioknapper – kun ett svar per spørsmål Innstillinger Minimer Kopier Slett

28. Inferences from twitch interpolation

Which of the statements below best summarizes the inferences that can be made when a deficit (ie, fatigue) in percentage voluntary activation is observed?

☐ Obligatorisk spørsmål

Svaralternativer Rediger som tekst **Forvalgt**

Lower levels of percentage voluntary activation suggest a participant i SI
Lower levels of percentage voluntary activation suggest a participant i SI

Legg til svaralternativ

Matrise – ett svar per spørsmål Innstillinger Minimer Kopier Slett

29.1. Methodological limitations - stimulation parameters

Rate the effect that each stimulation parameter may have on validity. *Note: please select your rating assuming a twin / doublet stimulation method.*

Rader **Obligatorisk**

Maintenance of constant and optimal contact between the stimulating pe ☐ Slett

Legg til rad

Kolonner Rediger som tekst **Forvalgt**

1. Completely limits the validity of the method. SI
2. Moderate-to-major effect on validity. SI
3. Has no influence, or a minor effect, on validity. SI

Legg til kolonne

Matrise – ett svar per spørsmål Innstillinger Minimer Kopier Slett

29.2. Methodological limitations - participant factors

Rate the effect that each participant related factor may have on validity. *Note: please select your rating assuming a twin / doublet stimulation method.*

Rader **Obligatorisk**

During investigations, it is difficult to ensure participants are contracting | Slett

The measure may be dependent on the muscle group being assessed (| Slett

Legg til rad

Kolonner Rediger som tekst **Forvalgt**

1. Completely limits the validity of the method. | Sl
2. Moderate-to-major effect on validity. | Sl
3. Has no influence, or a minor effect, on validity. | Sl

Legg til kolonne

Matrise – ett svar per spørsmål Innstillinger Minimer Kopier Slett

29.3.1. Methodological limitations - internal validity

Rate the effect that each proposed factor may have on the internal validity of the method. *Note: please select your rating assuming a twin / doublet stimulation method.*

Rader **Obligatorisk**

The size of the superimposed twitch is limited by antidromic collisions at | Slett

Legg til rad

Kolonner Rediger som tekst **Forvalgt**

1. Moderate-to-major effect on validity. | Sl
2. Has no influence, or a minor effect, on validity. | Sl

Legg til kolonne

Matrise – ett svar per spørsmål Innstillinger Minimer Kopier Slett

29.3.2 Methodological limitations - internal validity

Rate the effect that each proposed factor may have on the internal validity of the method. *Note: please select your rating assuming a twin / doublet stimulation method.*

Rader **Obligatorisk**

The method is insensitive to small changes in activation at high levels o | Slett

Legg til rad

Kolonner Rediger som tekst **Forvalgt**

1. Major effect on validity. | Sl
2. Moderate effect on validity. | Sl

Legg til kolonne

Matrise – ett svar per spørsmål Innstillinger Minimer Kopier Slett

29.4. Methodological limitations - ecological validity

Rate the effect that each proposed factor may have on the ecological validity of the method. *Note: please select your rating assuming a twin / doublet stimulation method.*

Rader **Obligatorisk**

Deficits observed in lab-based settings (e.g., often isometric, single joint | Slett

Legg til rad

Kolonner Rediger som tekst

Forvalgt

1. Moderate-to-major effect on validity.	◯	Sl
2. Has no influence, or a minor effect, on validity.	◯	Sl

Legg til kolonne

-
