# Supplementary material for: Neuromuscular electrical stimulation during maximal voluntary contraction: a Delphi survey with expert consensus
Source: Eur J Appl Physiol. 2023 May 29;123(10):2203–12. doi: 10.1007/s00421-023-05232-1 (PMC10492693; doi:10.1007/s00421-023-05232-1)
Supplement: Supplementary file 3 — Supplementary file3 (PDF 216 KB) [file 421_2023_5232_MOESM3_ESM.pdf]

Supplement 3

Closed-ended responses from Delphi Round 1 (n = 30)

Question	Responses	N	%
**1. Description of outcome measure**	1. Voluntary activation.	13	43.3
	2. Voluntary activation level.	13	43.3
	3. Percentage voluntary activation.	2	6.7
	4. Other (see Supplementary 4)	2	6.7
**2. Outcome measure definition**	1. The amount of force voluntarily produced during contraction as a proportion of the maximal possible force.	13	43.3
	2. The level of central/descending drive to the motoneuron pool during voluntary contraction.	4	13.3
	3. The level of inactivation during a maximum voluntary contraction.	5	16.7
	4. The qualitative level of muscle activation during maximum voluntary contraction.	3	10.0
	5. Other (see Supplement 4)	5	16.7
**3. Validity of twitch interpolation for assessment of VA**	1. Yes, the method always provides a valid estimate of voluntary activation, or, inactivation, regardless of the population being assessed or the setting	2	6.7
	2. The method provides a valid estimation of voluntary activation, or suboptimal activation but only in specific instances (e.g., when it is certain the participant is contracting maximally, or in specific clinical populations or settings)	27	90.0
	3. No, the method does not provide a valid estimate of voluntary activation, or incomplete activation	1	3.3
**4. Stimulation location – muscle vs. nerve**	1. Only nerve stimulation provides a valid assessment of voluntary activation.	7	23.3
	2. Both muscle and nerve stimulation can provide a valid assessment of voluntary activation (see 4.1)	22	73.3
**4.1 Muscle and nerve validity.**	1. Muscle and nerve stimulation are equally valid.	9	30.0
	2. Nerve stimulation has a higher validity than muscle stimulation.	13	43.3
**1. Pulse width**	1. 0.1 ms	3	10.0
	2. 0.2 ms	15	50.0
	3. 0.4 ms	2	6.7
	4. 0.5 ms	3	10.0
	5. 1.0 ms	7	23.3
**2. Number of stimuli**	1. 1 stimuli	8	26.7

	2. 2 stimuli (doublets)	20	66.7
	3. More than 2 stimuli	2	6.7
**3. Stimulation source**	1. Stimulating electrodes provide the best electrical stimulus for muscle/nerve	14	46.7
	2. A stimulating pen provides the best electrical stimulus for muscle/nerve	3	10.0
	3. Both stimulating methods are equally effective in eliciting an electrical stimulus	13	43.3
**4. Electrode size & type**	1. Metal plate electrode	3	10.0
	2. Self-adhesive electrode	24	80.0
	3. Stimulating pen	3	10.0
**Cathode size (width x length)**			
**9.1 - Femoral nerve/quadriceps**			
	1. Stimulating pen	4	13.3
	2. 3.2cm (round)	10	33.3
	3. 5cm (round)	3	10.0
	4. 3 x 5cm	2	6.7
	5. 5 x 5cm	1	3.3
	6. 5 x 9cm	2	6.7
	7. 7.5 x 13cm	3	10.0
	8. Other (see Supplement 4)	3	10.0
**9.2 - Tibial nerve/plantar flexors**			
	1.Stimulating pen	2	6.7
	2. 3.2cm (round)	12	40.0
	3. 5cm (round)	1	3.3
	4. 3 x 5cm	1	3.3
	5. 7.5 x 10cm	1	3.3
	6. Other (see Supplement 4)	5	16.7
**9.3 - Common peroneal nerve / dorsiflexors**			
	1. Stimulating pen	2	6.7
	2. 3.2cm (round)	7	23.3
	3. Other (see Supplement 4)	5	16.7
**Anode size (width x length)**			
**9.4 - Femoral nerve/quadriceps**			
	1. Stimulating pen	1	3.3

	2. 3.2cm (round)	4	13.3
	3. 5cm (round)	1	3.3
	4. 3 x 5cm	5	16.7
	5. 5 x 5cm	2	6.7
	6. 5 x 9cm	10	33.3
	7. 7.5 x 10cm	2	6.7
	8. 7.5 x 13cm	4	13.3
**9.5 - Tibial nerve/plantar flexors**			
	1. Stimulating pen		
	2. 3.2cm (round)	4	13.3
	3. 5cm (round)	2	6.7
	4. 3 x 5cm	3	10.0
	5. 5 x 5cm	1	3.3
	6. 5 x 9cm	10	33.3
	7. 7.5 x 10cm	1	3.3
	8. Other (see Supplement 4)	2	6.7
**9.6 - Common peroneal nerve/dorsiflexors**			
	1. Stimulating pen	1	3.3
	2. 3.2cm (round)	3	10.0
	3. 5cm (round)	1	3.3
	4. 3 x 5cm	2	6.7
	5. 5 x 5cm	1	3.3
	6. 5 x 9cm	4	13.3
	7. Other (see Supplement 4)	3	10.0
**15. Familiarizati**on	1. 1 session	23	76.7
	2. 2 sessions	3	10.0
	3. More than 2 sessions	3	10.0
**17. Number of contractions completed**	1. 2 contractions	3	10.0
	2. 3 contractions	14	46.7
	3. 4 contractions	2	6.7
	4. 5 contractions	2	6.7
	5. >5 contractions	1	3.3
	6. Other (see Supplement 4)	8	26.7

**19. Provision of feedback**	1. Essential	21	70.0
	2. Somewhat essential	5	16.7
	3. Neutral	2	6.7
	4. Somewhat unnecessary	2	6.7
	5. Unnecessary		
**19.1 Timing of feedback**	1. Real time feedback	20	66.7
	2. Feedback during rest periods	5	16.7
	3. Other (see Supplement 4)	5	16.7
**20. Number of contractions used for analysis**	1. Average of several contractions	7	23.3
	2. Single contraction (peak value)	17	56.7
	3. Other (see Supplement 4)	6	20.0
**20.1 Number of contractions used for average**	1. 2 contractions	2	6.7
	2. 3 contractions	3	10.0
	3. 5 contractions	1	3.3
**20.2 – Assessment of fatigue**	1. Average of several contractions	3	10.0
	2. Single contraction	26	86.7
**21 VA Calculation**	1. VA = 100 – D × (Maximum evoked force ÷ maximum voluntary force) ÷ resting twitch amplitude × 100	10	33.3
	2. VA = (1 – superimposed twitch ÷ resting twitch amplitude) × 100	17	56.7
**22. Dynamic vs. isometric contractions**	1. Can be used during dynamic contractions	4	13.3
	2. May be suitable in particular instances, during dynamic contractions	18	60.0
	3. Cannot be used during dynamic contractions	8	26.7
**24. Twitch interpolation vs. central activation ratio (CAR)**	1. Twitch interpolation provides a more valid estimate	22	73.3
	2. Both methods provide a valid estimate	7	23.3
	3. CAR provides a more valid estimate	1	3.3
**25.1 Rest between contractions (no**	1. >2 minutes	8	26.7

**time constraint)**	2. 1-2 minutes	12	40.0
	3. 30-60 seconds	8	26.7
	4. 0-30 seconds	2	6.7
**25.2 Rest between contractions (time constraint)**	1. >2 minutes	1	3.3
	2. 30-60 seconds	4	13.3
	3. 0-30 seconds	15	50.0
	4. No rest	10	33.3
**26. Evoked voluntary force relationship**	1. Extrapolating the evoked/voluntary force relationship provides a more valid measure	4	13.3
	2. Neither method offers superior validity in terms of estimating voluntary activation	7	23.3
	3. Comparing superimposed and resting potentiated twitches as a ratio provides a more valid measure	18	60.0
**27. Stimulation of antagonists**	1. Antagonist stimulation is unavoidable	3	10.0
	2. Stimulation will always result in partial stimulation of antagonist muscles	21	70.0
	3. It is possible to selectively stimulate the agonist muscle	6	20.0
**28. Inferences from twitch interpolation**	1. The completeness of voluntary activation cannot be determined by electrical stimulation during maximum voluntary contractions	1	3.3
	2. Lower levels of percentage voluntary activation suggest a participant is not capable of producing their true maximum force	10	33.3
	3. Lower levels of percentage voluntary activation suggest a participant is not capable of producing their true maximum force due to a sub-optimal neural drive to the muscle	16	53.3
	4. Lower levels of percentage voluntary activation suggestion a participant is unable to produce their true maximal force due to lower levels of central input to the motoneuron pool	1	3.3
	5. Other (see Supplement 4)	2	6.7
**Methodological limitations**			
**29.1 Stimulation parameters**	It is difficult to confine stimulation to the agonist muscle (a portion of antagonist motoneurons are recruited)		
	1. Majorly limits the validity	2	6.7
	2. Moderate effect on validity	6	20.0
	3. A minor effect on validity	21	70.0
**29.2 Stimulation parameters**	Maintenance of constant and optimal contact between the stimulating pen/electrodes and skin		
	1. Completely limits the validity of the method	1	3.3
	2. Majorly limits the validity	10	33.3
	3. Moderate effect on validity	8	26.7
	4. A minor effect on validity	8	26.7

	5. Has no influence on validity	1	3.3
**29.3 Participant related factors**	During investigations, it is difficult to ensure participants are contracting maximally		
	1. Completely limits the validity of the method	4	13.3
	2. Majorly limits the validity	11	36.7
	3. Moderate effect on validity	9	30.0
	4. A minor effect on validity	4	13.3
**29.4 Participant related factors**	The measure may be dependent on the muscle group being assessed (e.g., muscle groups with generally high activation capacities will be insensitive to changes in voluntary activation)		
	1. Completely limits the validity of the method	1	3.3
	2. Majorly limits the validity	6	20.0
	3. Moderate effect on validity	12	40.0
	4. A minor effect on validity	8	26.7
	5. Has no influence on validity	2	6.7
**29.5 Internal validity of the method**	The size of the superimposed twitch is limited by antidromic collisions at strong contraction intensities		
	1. Majorly limits the validity	3	10.0
	2. Moderate effect on validity	5	16.7
	3. A minor effect on validity	19	63.3
	4. Has no influence on validity	2	6.7
**29.6 Internal validity of the method**	The method is insensitive to small changes in activation at high levels of voluntary force		
	1. Completely limits the validity of the method	2	6.7
	2. Majorly limits the validity	5	16.7
	3. Moderate effect on validity	17	56.7
	4. A minor effect on validity	4	13.3
	5. Has no influence on validity	1	3.3
**29.7 Ecological validity of the method**	Deficits observed in lab-based settings (e.g., often isometric, single joint contractions) do not transfer to real-word movements (e.g., dynamic, multi-joint contractions)		
	1. Majorly limits the validity	11	36.7
	2. Moderate effect on validity	11	36.7
	3. A minor effect on validity	4	13.3
	4. Has no influence on validity	3	10.0
**30. Populations where the measure provides a meaningful assessment of voluntary activation (can select multiple responses)**	1. Highly resistance trained athletes	25	83.3
	2. Highly aerobically trained athletes	25	83.3
	3. Healthy younger (<60 years) participants	26	86.7
	4. Healthy older (>60 years) participants	25	83.3
	5. Stroke patients	16	53.3

	6. Cerebral Palsy	8	26.7
	7. Other (see Supplement 4)	6	20.0
**31. Settings where twitch interpolation provides a useful assessment of voluntary activation (can select multiple responses)**	1. Post-injury (e.g., muscle strain, tendinopathy)	16	53.3
	2. Following surgery (e.g., hip replacement, ACL reconstruction)	17	56.7
	3. Following a training intervention (e.g., resistance training)	27	90.0
	4. To determine the effects of fatigue induced by exhaustive exercise (e.g., repeated sprinting)	27	90.0
	5. To determine the effects of ergogenic aids (e.g., caffeine)	20	66.7
	6. Other (see Supplement 4)	2	6.7

Note: VA = voluntary activation

Article Details:
Osborne, John O.[1*]; Tallent J, Girard O, Marshall P, Kidgell D, Buhmann R. Neuromuscular electrical stimulation during maximal voluntary contraction: a Delphi survey with expert consensus. *European Journal of Applied Physiology*.

*Corresponding Author
Dr. John O. Osborne
School of Sport Sciences, UiT The Arctic University of Norway, Tromsø, Norway.
Address: Medisin- og helsebygget, UiT, Tromsø, Norway, 9037.
ORCID: 0000-0001-8681-8521
E-mail: john.osborne@uqconnect.edu.au
