# Supplementary material for: Neuromuscular electrical stimulation during maximal voluntary contraction: a Delphi survey with expert consensus
Source: Eur J Appl Physiol. 2023 May 29;123(10):2203–12. doi: 10.1007/s00421-023-05232-1 (PMC10492693; doi:10.1007/s00421-023-05232-1)
Supplement: Supplementary file 4 — Supplementary file4 (PDF 227 KB) [file 421_2023_5232_MOESM4_ESM.pdf]

Supplement 4

Closed-ended responses from Delphi Round 2 (n = 26)

Question	Responses	N	%
**1. Description of outcome measure**	1. Voluntary activation.	12	46.2
	2. Voluntary activation level.	13	50.0
**2. Outcome measure definition**	1. The amount of force voluntarily produced during contraction as a proportion of the maximal possible force.	15	57.7
	2. The level of inactivation during a maximum voluntary contraction.	10	38.5
**4.1 Muscle and nerve validity.**	1. Both muscle and nerve stimulation can provide a valid assessment of voluntary activation.	13	50.0
	2. Nerve stimulation has a higher validity than muscle stimulation.	13	50.0
**6. Pulse width**	1. 0.1-0.2 ms	12	46.2
	2. 0.4-0.5 ms	7	26.9
	3. 1.0 ms	6	23.1
**7. Number of stimuli**	1. 1 stimulus	7	26.9
	2. 2 stimuli (doublets)	19	73.1
**8. Stimulation source**	1. Stimulating electrodes provide the best electrical stimulus for muscle/nerve	16	61.5
	2. Both stimulating methods are equally effective in eliciting an electrical stimulus	9	34.6
**9.1 Cathode size (width x length)**			
**Femoral nerve/quadriceps**			
	1. Stimulating pen	5	19.2
	2. Small (3.2 or 5 cm (round)); 3 x 5 cm (width x length))	13	50.0
	3. Medium (5 x 5 cm; 5 x 9 cm (width x length))	3	11.5
	4. Large (5 x 13 cm; 7.5 x 10 cm; 7.5 x 13 cm (width x length))	5	19.2
**Tibial nerve/plantar flexors**			
	1. Stimulating pen	6	23.1
	2. Small (3.2 or 5 cm (round)); 3 x 5 cm (width x length))	11	42.3

	3. Medium (5 x 5 cm; 5 x 9 cm (width x length))	4	15.4
	4. Large (5 x 13 cm; 7.5 x 10 cm; 7.5 x 13 cm (width x length))	1	3.8
**Common peroneal nerve / dorsiflexors**			
	1. Stimulating pen	5	19.3
	2. Small (3.2 or 5 cm (round)); 3 x 5 cm (width x length))	12	46.2
	3. Medium (5 x 5 cm; 5 x 9 cm (width x length))	1	3.8
	4. Large (5 x 13 cm; 7.5 x 10 cm; 7.5 x 13 cm (width x length))	0	0
**9.2 Anode size (width x length)**			
**Femoral nerve/quadriceps**			
	1. Stimulating pen	0	0
	2. Small (3.2 or 5 cm (round)); 3 x 5 cm (width x length))	5	19.2
	3. Medium (5 x 5 cm; 5 x 9 cm (width x length))	12	46.2
	4. Large (5 x 13 cm; 7.5 x 10 cm; 7.5 x 13 cm (width x length))	9	34.6
**Tibial nerve/plantar flexors**			
	1. Stimulating pen	0	0
	2. Small (3.2 or 5 cm (round)); 3 x 5 cm (width x length))	7	26.9
	3. Medium (5 x 5 cm; 5 x 9 cm (width x length))	14	53.8
	4. Large (5 x 13 cm; 7.5 x 10 cm; 7.5 x 13 cm (width x length))	2	7.7
**Common peroneal nerve/dorsiflexors**			
	1. Stimulating pen	0	0
	2. Small (3.2 or 5 cm (round)); 3 x 5 cm (width x length))	13	50.0
	3. Medium (5 x 5 cm; 5 x 9 cm (width x length))	5	19.2
	4. Large (5 x 13 cm; 7.5 x 10 cm; 7.5 x 13 cm (width x length))	1	3.8
**10.1 Rate strategies from most, to least, useful for reducing pain during stimulation.**			
**Decreasing the pulse width.**			
	1. Most effective.	0	0
	2. Somewhat effective.	1	3.8
	3. Moderately effective.	13	50.0
	4. Not very effective.	4	15.4
	5. Least effective.	4	15.4

**Reducing pulse duration (width) and simulating with a higher current**	1. Most effective.	0	0
	2. Somewhat effective.	1	3.8
	3. Moderately effective.	10	38.5
	4. Not very effective.	5	19.2
	5. Least effective.	5	19.2
**Increasing pulse duration (width) and stimulating with a lower current**	1. Most effective.	5	19.2
	2. Somewhat effective.	4	15.4
	3. Moderately effective.	7	26.9
	4. Not very effective.	3	11.5
	5. Least effective.	2	7.7
**Familiarising participants with stimulation in separate familiarisation session/s**	1. Most effective.	12	46.2
	2. Somewhat effective.	9	34.6
	3. Moderately effective.	3	11.5
	4. Not very effective.	0	0
	5. Least effective.	0	0
**Encouraging maximal contractions**	1. Most effective.	11	42.3
	2. Somewhat effective.	9	34.6
	3. Moderately effective.	3	11.5
	4. Not very effective.	0	0
	5. Least effective.	1	3.8
**10.2 Recommendations to reduce pain experienced by participants during stimulation (can select multiple responses)**	1. Decreasing the pulse width.	1	3.8
	2. Reducing pulse duration (width) and simulating with a higher current.	2	7.7
	3. Increasing pulse duration (width) and stimulating with a lower current.	9	34.6
	4. Familiarising participants with stimulation in separate familiarisation session/s.	23	88.5
	5. Encouraging maximal contractions.	18	69.2

**11.1 Preferred intratester reliability measure.**	1. Coefficient of variation (CV).	15	57.7
	2. Intraclass correlation coefficients (ICCs).	10	38.5
	3. Standard error of measurement (SEM).	1	3.8
**11.2 Second most preferred intratester reliability measure.**	1. Coefficient of variation (CV).	9	34.6
	2. Intraclass correlation coefficients (ICCs).	11	42.3
	3. Standard error of measurement (SEM).	6	23.1
**11.3 Least preferred intratester reliability measure.**	1. Coefficient of variation (CV).	2	7.7
	2. Intraclass correlation coefficients (ICCs).	5	19.2
	3. Standard error of measurement (SEM).	19	73.1
**12.1. Current increase for ramp procedure, when determining stimulation during VA assessment?**	1. 10 mA of less.	7	26.9
	2. 20 mA.	12	46.2
	3. 30 mA	3	11.5
	4. 50 mA or greater.	3	11.5
**12.1. Increase of maximal stimulus during ramp procedure for supramaximal stimulus.**	1. 10%	3	11.5
	2. 20%	18	69.2
	3. 30%	4	15.4
**13. What % voluntary activation capacity is 'maximal'?**	1. ≥85%	0	0
	2. ≥90%	10	38.5
	3. ≥95%	9	34.6
	4. 100%	6	23.1
**16.1 Preferred within-session reliability measure.**	1. Coefficient of variation (CV).	14	53.8
	2. Intraclass correlation coefficients (ICCs).	11	42.3
	3. Standard error of measurement (SEM).	1	3.8
**16.2 Second most preferred within-session reliability measure.**	1. Coefficient of variation (CV).	10	38.5
	2. Intraclass correlation coefficients (ICCs).	9	34.6
	3. Standard error of measurement (SEM).	7	26.9

**16.3 Least preferred within-session reliability measure.**	1. Coefficient of variation (CV).	4	15.4
	2. Intraclass correlation coefficients (ICCs).	5	19.2
	3. Standard error of measurement (SEM).	17	65.4
**17. Number of contractions completed.**	1. At least 1 contraction.	0	0
	2. Minimum of 2 contractions.	10	38.5
	3. Minimum of 3 contractions.	13	50.0
	4. Minimum of 4 contractions.	0	0
	5. Minimum of 5 contractions.	3	11.5
**18.1 Which instruction results in the consistent assessment of VA?**	1. Verbal encourage (e.g., "when you contract, we want you to contract as hard and as fast as possible and continue contracting through the stimulus").	13	50.0
	2. Using feedback from the torque/force-time trace and instructing participants to make their trace go as high as possible.	13	50.0
**18.2 Are both options in 18.1 effective?**	1. Yes - if my selected option in Q18.1 was not possible to use, the alternative would still be acceptable.	20	76.9
	2. No - only the answer I selected in Q18.1 was acceptable.	6	23.1
**19.1 Provision of feedback – timing.**	1. Real-time feedback.	21	80.8
	2. Feedback during rest periods.	5	19.2
**21. VA Calculation**	1. 100 – D × (Maximum evoked force ÷ maximum voluntary force) ÷ resting twitch amplitude × 100	9	34.6
	2. (1 – superimposed twitch ÷ resting twitch amplitude) × 100	16	61.5
**22. Dynamic vs. isometric contractions**	1. Cannot be used during dynamic contractions.	10	38.5
	2. Can be used during dynamic contractions.	13	50.0
**23.1. Which triggering method results in the most consistent and accurate assessment of VA?**	1. Manually triggering the electrical stimulus once the force/torque trace reaches a visible plateau.	20	76.9
	2. Automatically triggering the electrical stimulus using pre-set commands.	6	23.1
**23.2. Are both options described in Question 23.1 are effective?**	1. Yes - if my selected option in Q23.1 was not possible to use, the alternative would still be acceptable.	15	57.7
	2. No - only the answer I selected in Q23.1 was acceptable.	11	42.3
**25.1 Rest between contractions**	1. No rest (0 seconds).	3	11.5

**(fatigue)**	2. 1 – 30 seconds rest.	14	53.8
	3. More than 30 seconds rest.	9	34.6
**28. Inferences from twitch interpolation**	1. Lower levels of percentage voluntary activation suggest a participant is not capable of producing their true maximum force.	15	57.7
	2. Lower levels of percentage voluntary activation suggest a participant is not capable of producing their true maximum force due to a sub-optimal neural drive to the muscle.	11	42.3
**Methodological limitations**			
**29.1 Stimulation parameters**	Maintenance of constant and optimal contact between the stimulating pen/electrodes and skin		
	1. Completely limits the validity of the method.	15	57.7
	2. Moderate to major effect on validity.	9	34.6
	3. Has no influence, or a minor effect, on validity.	0	0
**29.3 Participant related factors**	During investigations, it is difficult to ensure participants are contracting maximally		
	1. Completely limits the validity of the method.	13	50.0
	2. Moderate to major effect on validity.	9	34.6
	3. Has no influence, or a minor effect, on validity.	3	11.5
**29.4 Participant related factors**	The measure may be dependent on the muscle group being assessed (e.g., muscle groups with generally high activation capacities will be insensitive to changes in voluntary activation)		
	1. Completely limits the validity of the method.	3	11.5
	2. Moderate to major effect on validity.	14	53.8
	3. Has no influence, or a minor effect, on validity.	7	26.9
**29.5 Internal validity of the method**	The size of the superimposed twitch is limited by antidromic collisions at strong contraction intensities		
	1. Moderate to major effect on validity.	10	38.5
	2. Has no influence, or a minor effect, on validity.	14	53.8
**29.6 Internal validity of the method**	The method is insensitive to small changes in activation at high levels of voluntary force.		
	1. Major effect on validity.	10	38.5
	2. Moderate effect on validity.	15	57.7
**29.7 Ecological validity of the method**	Deficits observed in lab-based settings (e.g., often isometric, single joint contractions) do not transfer to real-word movements (e.g., dynamic, multi-joint contractions)		
	1. Moderate to major effect on validity.	22	84.6
	2. Has no influence, or a minor effect, on validity.	3	11.5

Note: VA = voluntary activation.

Article Details:

Osborne, John O.[1*]; Tallent J, Girard O, Marshall P, Kidgell D, Buhmann R. Neuromuscular electrical stimulation during maximal voluntary contraction: a Delphi survey with expert consensus. *European Journal of Applied Physiology*.

*Corresponding Author
Dr. John O. Osborne
School of Sport Sciences, UiT The Arctic University of Norway, Tromsø, Norway.
Address: Medisin- og helsebygget, UiT, Tromsø, Norway, 9037.
ORCID: 0000-0001-8681-8521
E-mail: john.osborne@uqconnect.edu.au
