# Supplementary material for: Neuromuscular electrical stimulation during maximal voluntary contraction: a Delphi survey with expert consensus
Source: Eur J Appl Physiol. 2023 May 29;123(10):2203–12. doi: 10.1007/s00421-023-05232-1 (PMC10492693; doi:10.1007/s00421-023-05232-1)
Supplement: Supplementary file 5 — Supplementary file5 (PDF 393 KB) [file 421_2023_5232_MOESM5_ESM.pdf]

Supplement 5

Delphi Round 1: open ended responses and comments.

Question	Open Responses
**1. Description of outcome measure**	Maximal voluntary activation.
**- Other**	To me, number one above voluntary activation and when using peripheral measure and TMS they should be delineated by VAmns and VAtms.
**2. Outcome measure definition**	The maximal degree to which one is capable of voluntarily activating his/her muscle during a maximal voluntary contraction.
**- Other**	[I] believe it depends on the specific technique used.
	Neural drive to the muscle.
	The level of muscle activation during maximum voluntary contraction.
	The percent or proportion activation of the muscle relative to the maximum possible under a specific set of conditions. Note that it's not a measure of central drive to the muscle; if conditions at the muscle change then %VA will also change regardless of the actual level of central drive. That is, under some conditions, more or less descending drive is required to achieve 'full activation' and we can only determine our current capacity as a percent/proportion of that level.
**4.1.1 Stimulation location – muscle vs. nerve**	Triplets might be better than twitches, since triplets produce maximal responses in terms of torque production (de Haan, 1998), thereby limiting the sensitivity to, for instance, length-dependent changes in calcium sensitivity and post-tetanic potentiation and improving the signal-to-noise ratio.
	To me, the nerve stimulation provide a better assessment of VA at least when antagonist muscles are minimally activated during the electrical stimulation (e.g. adductor pollicis).
	Muscle stimulation may stimulate the antagonist which should be avoided (see Awiszus et al. 1997, Muscle Nerve 20:1187).
	Either stimulation over the muscle, which activates the intramuscular motor nerve fibres, or peripheral nerve stimulation can be "valid" or "not valid" for voluntary activation, depending on which nerve and/or muscle belly is stimulated and how the response is measured (e.g. stimulation of the femoral nerve for knee extensor voluntary activation is a good choice because the femoral nerve mostly innervates knee extensors [apart from sartorius], whereas stimulation of a peripheral nerve for elbow flexor activation is difficult because stimulation of multiple nerves would be required and in practical terms it is difficult to place electrodes to provide consistent stimulation).
	Personally, I have only done nerve stimulation, so my answer could be considered biased.
	Of course, such validity is muscle-dependent.

It depends on the muscle. For example, for the elbow flexor muscles, only muscle belly stimulation is valid. for the FDI, nerve stimulation is valid.

It would also depend on how much of the antagonist muscle is activated by the stimulation - could be for either method. I'd say that nerve stimulation 'can' provide a valid assessment. Theoretically, muscle belly stimulation might under some very specific conditions, but it's rare to be able to stimulate (near to) the full volume of a muscle using muscle belly stimulation.

**5.1. Anode and cathode placement – Femoral**	On the inguinal triangle (moving cathode around until find maximal M-Wave)<br><br>The cathode (5x5 cm) at the femoral nerve. The anode (13x8 cm) over the gluteal fold.<br><br>Cathode over the femoral nerve in the femoral triangle. Anode over the gluteus medius/between the greater trochanter and the iliac crest/gluteal fold OR distal to the cathode over the muscle. In our experience, the opposite configuration will also yield similar results.<br><br>Cathode placed over the femoral nerve at the femoral triangle level beneath the inguinal ligament and the anode placed on the lower part of the gluteal fold, opposite to the cathode.<br><br>Anode over femoral nerve (around inguinal crease) and cathode over femoral head.<br><br>Cathode: over the femoral nerve in the femoral triangle. Anode: midway between the greater trochanter and iliac crest. The optimal site of stimulation (greatest response for a given stimulus intensity) is determined by using a pen electrode.<br><br>Both electrodes above the nerve at the hip.<br><br>The anode is rectangular pad (10 x 5 cm) placed over the greater trochanter inclined towards the most lateral part of gluteal fold. The cathode is placed over the femoral nerve in the femoral triangle.<br><br>Near the pulse of femoral artery and gluteal fold<br><br>See Besson MSSE 2021 for instance<br><br>Cathode - over the femoral nerve near the inguinal ligament. Anode - not critical - somewhere between the greater trochanter and superior iliac spine<br><br>Anode: lateral aspect of the hip, inferior to the iliac crest. Cathode: femoral triangle (specific location determined by largest M-wave).<br><br>Cathode on the femoral nerve (groin), anode gluteal fold.<br><br>Anode (large) positioned midway between the superior aspect of the greater trochanter of the leg and the inferior border of the iliac crest (hip flexed 90°), ball-shaped cathode-stimulating electrode (3 cm) positioned in the femoral triangle over the femoral nerve.<br><br>Cathode: over the femoral nerve on the femoral triangle, 3-5 cm below the inguinal ligament. Anode: under the gluteal fold.<br><br>Anode: gluteal fold. Cathode: femoral triangle.<br><br>Cathode: femoral triangle level beneath the inguinal ligament. Anode: gluteal fold opposite the cathode.<br><br>Cathode electrode: at the femoral triangle level beneath the inguinal ligament (individually determined). Anode electrode: midway between the greater trochanter and the lower border of the iliac crest.

	Circular cathode (3 cm in diameter) is positioned upon the femoral nerve and a rectangular anode (8 x?4 cm) in the gluteus fossae.<br><br>Cathode: femoral triangle. Anode: gluteal fold.<br><br>The cathode over the nerve high in the femoral triangle, and the anode over the greater trochanter<br><br>Are we only using monophasic current waveforms that would have a true anode and a true cathode? If a stimulator output is a biphasic waveform, which are you considering a true anode or cathode? At maximal or supramaximal current intensities, I do not believe that if you are using the true anode or true cathode should matter--both will be able to cause axonal depolarization.<br><br>Femoral triangle and head of the greater trochanter. But as there are difficulties in securing the anode to provide even pressure in the femoral triangle, we have placed the anode and cathode side-by-side overlapping the femoral nerve.<br><br>The cathode is positioned on the femoral nerve, where the force is maximal for a given stimulation intensity (using the pen). In general, it is right at the crossroads between the inguinal ligament and the femoral nerve (it feels like a "ball" in lean participants).<br>The anode, is placed midway between the greater trochanter and the iliac crest.<br><br>Anode midway iliac crest and great trochanter AND cathode femoral triangle<br><br>Anode placed high over the femoral triangle with the cathode over the top of the respective gluteus maximus.<br><br>Anode: trochanter major. Cathode: femoral nerve in the femoral triangle.<br><br>Cathode approximately 0.5 cm medial and inferior to the femoral nerve and the anode about 2 cm lateral and superior to that position or over the gluteal fold. The cathode placement is then optimised by searching for the greatest M response to a low current stimulation. Ultrasound imaging may be used to ascertain the location of the femoral nerve.
**5.2. Anode and cathode placement – Tibial**	The cathode over the posterior tibial nerve in the popliteal fossa. The anode over the anterior aspect of the knee.<br><br>Depends. Bipolar pair electrode in popliteal fossa OR one in popliteal fossa and one over patella region<br><br>Cathode - Tibial nerve in the popliteal fossa. Anode - anterior knee over the patella OR distal to the cathode over the muscle. In our experience, the opposite configuration will also yield similar results.<br><br>Cathode placed in the popliteal fossa over the tibial nerve and the anode placed on the anterior surface of the knee.<br><br>Anode over tibial nerve in popliteal space and cathode on tibial plateau.<br><br>Cathode : over the tibial nerve in the popliteal fossa. Anode: above the patella. The optimal site of stimulation (greatest response for a given stimulus intensity) was determined by using a pen electrode.<br><br>Both electrodes above the nerve in the poplitea.<br><br>Bar stimulating electrode in popliteal fossa over the nerve.<br><br>See Besson MSSE 2021 for instance.

	Cathode: over the tibial nerve in the popliteal fossa. Anode: not critical - over the medial condyle of the femur.
	Anode: patella. Cathode: popliteal fossa (specific location determined by largest M-wave).
	Cathode: popliteal fossa. Anode: anterior surface of the knee.
	Anode: just below the patella. Cathode: popliteal fossa.
	Cathode: popliteal fossa. Anode: anterior surface of the knee (above the patella)
	Cathode electrode: over the tibial nerve in the popliteal fossa (individually determined). Anode: 3-4 cm below the inferior part of the right patella.
	Cathode: electrode placed on the popliteal fossa and parallel to the nerve. Anode: Patella.
	Are we only using monophasic current waveforms that would have a true anode and a true cathode? If a stimulator output is a biphasic waveform, which are you considering a true anode or cathode? At maximal or supramaximal current intensities, I do not believe that if you are using the true anode or true cathode should matter--both will be able to cause axonal depolarization.
	Popliteal fossa and patella.
	Anode patellar tendon AND cathode popliteal fossa.
	Bar electrode used over popliteal crease to stimulate the tibial nerve.
	Anode: below or above the patella. Cathode: posterior tibial nerve in the popliteal fossa.
	Anode placed on patella (although it may also be placed adjacent to the cathode) and cathode in the popliteal fossa overlying the nerve; often this electrode sits slightly lateral and inferior to the centre point of the popliteal crease. The cathode placement is then optimised by searching for the greatest M response to a low current stimulation.
**5.3. Anode and cathode placement - Common peroneal**	Common fibular nerve - bipolar bar pair just inferior and posterior to head of fibula.
	Cathode - Common peroneal nerve on the neck of the fibula. Anode - anterior knee over the patella OR distal to the cathode over the muscle. In our experience, the opposite configuration will also yield similar results.
	I do not have appropriate expertise for the common peroneal nerve
	Cathode : over the peroneal nerve close to the fibulat head. Anode: opposite side of the leg. The optimal site of stimulation (greatest response for a given stimulus intensity) was determined by using a pen electrode.
	Both electrodes above the nerve below the poplitea.
	Bar electrode positioned around the fibular head, depending on subjects nerve location, sometimes more deep or superficial
	I have had no success in measuring dorsiflexor voluntary activation, i.e. unable to find an appropriate electrode placement that does not also engage peroneal muscles (plantarflexors).

Anode: lateral aspect of the patella. Cathode: just below the fibula head.

I have not much experience with this technique.

I have never used the peroneal nerve stimulation

Are we only using monophasic current waveforms that would have a true anode and a true cathode? If a stimulator output is a biphasic waveform, which are you considering a true anode or cathode? At maximal or supramaximal current intensities, I do not believe that if you are using the true anode or true cathode should matter--both will be able to cause axonal depolarization.

Anode: below or above the patella. Cathode: common peroneal nerve close to the fibular head.

Anode placed over the central branch of the CPN so that the tibialis anterior M wave was evoked without M wave in peroneus longus. Cathode placed over or above the head of the fibula.

7.1 More than 2 stimuli - comments

Pulse train of three rectangular 200us pulses applied at 300 Hz. These high frequency stimulations(triplets) produce maximal responses in terms of torque production (de Haan, 1998), thereby limiting the sensitivity to, for instance, length-dependent changes in calcium sensitivity and post-tetanic potentiation and improving the signal-to-noise ratio.

In our experience, a triplet (three pulses) is slightly better than a doublet, as it improves the reliability of the resting twitch to which the superimposed twitch is normalized. The frequency we recommend is 50-100 Hz, preferably 100 Hz.

10. Reducing pain and discomfort during stimulation - comments

Use of 2 or more familiarization session (I noted discomfort reduces after 2 familiarization sessions). Use of single pulse.

A proper explanation and familiarization would help. I found that giving the twitch/triplet unexpectedly worked best (rather than cueing).

In our experience, pain/discomfort is related to the amount of torque elicited by the stimulus. This is the reason, the discomfort reduces substantially when the muscles are contracting maximally, as the electrical stimulus (though everything else [e.g., stimulus parameters, electrode size, etc.] remains the same) elicits only a small torque. This is probably the reason female participants take it better than male participants as the absolute torque is lower. Varying the pulse parameters (width, frequency, etc.) only has a minimal impact. The biggest impact can be had by using fewer pulses (e.g., a doublet or a triplet vs. 10 pulses). We recommend providing practice stimulus to orient the participant and also cueing.

Ensure that the electrodes are well positioned. If needed apply a constant pressure on the cathode using a strap or bandage.

Greater pulse width. Extensive familiarization.

Familiarization with stimulation during submaximal contractions before measurement onset

The most important for me is to reassure the participant. I explain to the participants that the pain is not related to damage and that the "current" does not travel in the body but remains "in the muscle".

I use large pulse width, and a lower current. As well, for baseline measures I find single twitches work fine.

Manual pressure on the electrode, familiarisation of the subjects, magnetic stimulation when possible.

Wider pulse widths with lower current can sometimes reduce sharp pain sensations. Information to participants that stimulation during the contraction will be much less painful than at rest and that the harder they contract the less it will hurt is helpful. Stimulation at rest or during weak contractions is more problematic.

Ensuring adequate time is allowed to locate the largest M-wave during preparation. Extensive familiarisation with the participant.

Habituation is essential, instruction: 'the more forceful you will contract, the smaller the response will be and you may hardly notice it.' The participant will of course know that he/she will receive a pulse but it is better if he/she doesn't see or hear any button being pressed by the investigator. When stimulating on a relaxed muscle: keep instructing the participant to relax and manually shake the muscle belly to check/enhance complete relaxation.

We experience no/minimal pain with the nerve stimulation - some with muscle.

1 - Familiarize the participants. 2 - Applying a pressure on the cathode reduce the pain/discomfort because it limits the intensity of current. 3 - Using doublets rather than multiple stimuli (even if this latter will provide a better assessment).

One session of familiarization (on a separate day) and an additional familiarization phase on the testing day (preceding the actual assessments).

1 or 2 familiarization sessions. Muscle belly stimulation instead of nerve stimulation. Use of submaximal stimulation intensity.

In order to reduce pain/discomfort, it is necessary to reduce the pulse width, increase electrode size and limit number of stimuli.

Familiarization is required

Practice is very important. Cueing is very important. Don’t use a large pulse width,

Using a lidocaine skin cream; making sure that the participant has not shaved their skin to which the electrodes will be applied; use of large electrodes appropriate for muscle size and motor point locations; using a phase/pulse duration (width is incorrect term) that is between 0.4 and 0.6 ms; keeping the stimulus as short as possible; and giving the participant experience with the stimulus at rest at varying current intensities.

We usually instruct our subjects to focus on something (e.g., mental task) during passive stimulation, and then instruct them to focus on the up-coming performance using sport psychology techniques (e.g. performance visualization or self-talk). Cueing informs the subject that the stimulation is coming son, but the exact timing is unknown. Further, our pulse width is typically 0.5 or (mostly) 1.0 ms for femoral nerve stimulation. To reduce overall discomfort, since we are not able to get reproducible H-reflex measures in the quads we use large increases in current to reduce the number of stimulations given to obtain maximal responses.

1) Taking the time needed to place the electrode to the best spot! 2) Using substantial pain as an excluding criteria to participate to the study.

Start with very low frequencies and apply a stepwise increase to assess threshold. The participant can then be familiarised with the feeling as it gets stronger, applying a higher current once threshold is established rarely causes any issues.

Considerable familiarisation, strong subject motivation, use of larger electrodes where possible. I've seen no negative effects of pulse width (and sometimes find less pain with wider pulses) as reductions in pulse width necessitate increases in current intensity. Applying constant pressure to cathode (or pushing ball electrode) to reduce electrode-nerve distance reduces need for high currents, but reliability needs to be affirmed. Dermal analgesia is, unfortunately, ineffective in our lab.

11.1 Intratester reliability – threshold values

CV < 2%

< 10%

CV < 5%

5%

<5%

ICC(2,1) should be larger than 0.7; SEM<10%

5%; 0.90

A measure of intratester reliability just says that the same tester gets the same result when performing the same test. It does not indicate whether the measure is a valid or accurate estimate of voluntary activation. e.g., if voluntary activation is always estimated at 100% because of a component of antagonist force in the twitch then the measure may be very reliable but not an accurate estimate.

We have reported twitch torque CV's between 3 and 7%

It certainly can be <5%

CV < 5%

CV < 5%; it is however unclear if you are referring to within-session or between-session reliability.

I do not think one value can be put here as it depends on the population tested, the muscle tested, the technique that has been used, etc...

CV: < 5%; ICC: > 0.75

<5%

<5%

0.7

In this case, I believe that you really are looking at test-retest reliability, as I would not expect the tester to contribute much to variation if the set-up and instructions are performed in a standardized process. I think it is difficult and not correct to have strict threshold values. In some patient populations, you may have widely varying VA depending on the physiological state of the person (muscle fatigue, fear/anxiety, pain, effusion, etc). In a "healthy" population, I would expect a more consistent level of VA; however, I do not necessarily expect physiological maximum performance to occur that frequently and to be repeatable in the same testing session. So I would need some more detail in this question--is it the same testing session or is it comparing between testing days; how long is the test-retest interval?

Probably <5% CV is acceptable, certainly in an acute fatigue study, but ideally it would be < 3% CV if assessing changes from an intervention.

CV < 5%; ICC > 0.9; SEM < 1%

CV =< 5%

3-5% test-re-test reliability.

In general, a CV below 5% is desirable. However, our own work has shown that the CV depends on the contraction mode with the lowest CV observed during isometric contraction (< 5%) and the highest for eccentric muscle actions (7%).

I admit I don't have a specific value for this, but it should be reported in all studies. Larger variability reduces the chance to search for relationships between variables, or changes in those variables, and necessitates larger sample sizes. But I don't have a threshold value that works as a blanket value across studies.

---

12. Determination of maximal stimulation intensity method

The current is increased by 5 mA at each step (starting at 30 mA), maximal stimulus intensity is determined when there is no further increase in twitch force amplitude and/or M-wave for 3 successive increases in current (<5% of increase), the supramaximal stimulus intensity is determined by adding 20% to the maximal stimulus intensity.

First, increase stimulation current until torque measured in response to a triplet levels off. Then increase current by another 20 mA to ensure supramaximal stimulation. It can be assumed that at this point all muscle fibres of the knee extensors are activated.

Depends... ideally max CMAP plus 15-20, or with pads tolerance or until force plateaus and antagonists not activated

We DO NOT use 120% supramaximal intensity. We prefer the maximal intensity because the torque associated with the stimuli actually decreases with supramaximal intensity, which reduces the peripheral torque and affects % activation calculation. For the quadriceps muscle, we start at 100 mA and increase the twitch ramp intensity by 100 mA until torque plateaus or decreases. We then reduce by 50 mA and then use whichever intensity produced the largest torque. For e.g., 100 mA, 200 mA, 300 mA, 400 mA, and 350 mA, if we started seeing the torque plateau or going down from 300 mA to 400 mA. If 350 mA produced the largest torque, we then use that intensity.

For experiments where the ramp needs to be standardized, a 20 mA increment is used until no further increases in the maximal peak twitch (and if recorded M-wave) are seen despite an additional increase of 20 mA. The supramaximal intensity is then set as 20% of the maximal intensity and the force and M-wave responses to this supramaximal stimulation are checked and compared to what observed for the maximal intensity. If any increases are observed, the maximal and supramaximal intensity are re-evaluated by continuing the ramp with 20-mA increments.
For experiments where the ramp does not need to be standardized, we initially increase the intensity by larger increments (40-50 mA), using the maximal intensity determined during the familiarization session as a reference, to minimize the number of stimulations delivered, until observing only small increases in peak twitch and M-waves. We then use 10 or 20-mA increments (depending on the muscle) until no further increases in peak twitch and M-waves are seen despite a further incremental increase to precisely determine the maximal intensity. As mentioned above, we then add 20% and re-evaluate the peak twitch and M-waves.

Increase by 10-20 mA until no further increase for two successive increases in current.

Depending on the muscle, usually the stimulus is increased by steps comprised between 1-2 mA (tibial nerve) and 10 mA (femoral nerve) until M-Wave and twitch force amplitude reached a plateau during ~5 successive increases in current.

I do not use a twitch ramp procedure in order to avoid stimulating the antagonist. I use 100 mA 0.5ms for muscle stimulation of the quadriceps for all subjects. I do not believe that supramaximal stimulation is necessary for valid VA estimation.

The current is increased by 20mA at each step, maximal stimulus intensity is determined when there is no further increase in twitch force amplitude for 3 successive increases in current, the supramaximal stimulus intensity is determined by adding 20% to the maximal stimulus intensity.

Depending on stim method (pad or nerve) I'll usually ramp up 20-50 mv per twitch, I monitor both the M-wave and twitch torque to determine a plateau, then increase current 10-30% and ensure there was not a further increase or decrease in torque.

Depend on the subjects (if familiarized, etc)

Current steps depend on the stimulus site, stimulus width and the individual. Steps continue until the twitch amplitude stops increasing for one to two current steps or if increases become very small. Decreasing current steps are then applied because the stimulation can cause potentiation of the twitch and the twitch amplitude often continues to grow despite lower intensity.

We have used an increase between 10 and 20 mA increments with 20 s rests between stimuli. These continue until we observe plateaus M-wave amplitudes as well as and the size the twitch torque. We have also used added 20 to 30% as our supramaximal stimulus intensity.

It is important that this procedure doesn't take too long, it may only make the participant nervous when too many small steps are taken. 20 mA steps might do it. When approaching the maximal, 10 mA steps may be preferred, plus a 30% increase after leveling off.

First optimal position identified then before each experiment, stimulus optimization was performed at rest by slowly increasing the intensity in steps of 20 mA until no further increase of force response and M-wave peak-to-peak amplitude resulted.

For knee and plantar flexor muscles: the intensity is increased by 10 mA at each step until the twitch force amplitude and the maximal M-wave amplitude reached a plateau (two successive increases). Then we used the first intensity where the plateau is reached and added between 20 and 50% of intensity (rather 20%).

Current intensity of a single pulse is progressively increased by 5-20 mA (depending on the subject and muscle group) until peak twitch torque plateaus for at least 3 consecutive current increases, then +20% to obtain stimulus supramaximality.

The current is increased by 10 to 20 mA at each step (depends on the level of familiarization of the participant with the technique and on the site of stimulation, for muscle stimulation can be more than 20 mA), maximal stimulus intensity is determined when there is no further increase in twitch force amplitude and M-wave amplitude for 2 successive increases in current, the supramaximal stimulus intensity is determined by adding 20% to the maximal stimulus intensity and the values are verified before implementation of the intensity.

The recruitment curves is achieved by progressively increasing the electrical stimulation intensity (minimal intensity: 10 mA; intensity increment: 5 mA, number of trials per intensity: 2; rest: 10 s). The optimal intensity (Iopt) was determined from recruitment curves [the intensity where unpotentiated single twitch (Qtwunpot) and concomitant compound muscle action potential (M-wave) amplitudes reached their maximal values and started to plateau. The supramaximal intensity (Isup) is set at 130 % of Iopt.

Femoral nerve electrical stimulations intensity (0.2 ms, 400 V) was increased until the EMG response of all muscles plateaued (Mmax) and then increased again by 20%.

Start at 50 mAmp, increase by 50s, intensity taken as the plateau of three twitch plus 20%.

I have used several ramp procedures to determine the stimulation intensity - often dependent on if the test was a part of a clinical visit or part of a isolated research study.
1. Lab-based scenario for the quadriceps in healthy young and older adults who were non-obese using large self-adhesive electrodes. We had pilot-

tested on 10 physically active subjects a ramp procedure increasing the stimulus 5 volts 10-15 seconds (using a 0.6 ms pulse and an 11 pulse train at 100 pps). All subjects had ceased increasing force by 120 volts, thus we set the stimulation intensity to 135 V for all subjects subsequent.
2. In some situations, like testing children, I have used a procedure to set the stimulation at the level the maximal force was produced. In this scenario, I used a 0.6ms phase/pulse duration and delivered a 13-pulse, 100 pps stimulus, delivered every 10-15 seconds at 5 V increments until two successive increments failed to produce an increase in force (with assent).
3. Testing the infraspinatus. We used a 3-pulse train of 0.6ms pulses delivered at 50 pps and increased the current in 5-10 V increments until 2 successive increases in voltage failed to improve force output.
4. In cases of VA testing during clinical visits for ACL & TKA, we used a set voltage maximum of 130-150 V (depending on device limits) for all testing. Again, in healthy young and older adults, this intensity appeared to be supramaximal in pilot testing.

For femoral nerve stimulation, increase of approximately 100 mA are used in the beginning and then increases 50 mA, then 20 mA when approaching force-response plateau. This is to reduce the number of stimulations needed. Typically, we obtain force plateau in our healthy young subjects around 500 mA. Thus, we aim to use <10 stimulations during our ramp procedures. Mostly I have used 20% additional current, but in some papers the increase was 25% and for tibial nerve stimulation our lab has also used 50%. Maximality is confirmed by three consecutive stimulations with no further increase in force response.

The current intensity is increased by 50 mA for each stimulus application. Maximal stimulation intensity is determined as that beyond which a further increase in current by 50 mA failed to increase the twitch force further.

The current is increased by 20 mA at each step, maximal intensity stimulus is determined when there is no further increase in both twitch force and M-wave for two successive increases in current, the supra maximal stimulus intensity is determined by adding 20% to the maximal stimulus intensity.

10 mA increase. Maximal intensity when no significant twitch force (and M-wave) increase after two successive increments. Then add 20% more.

Beginning from 20 mA, the current is increased by 20 mA and a maximal stimulus intensity is determined when there is no further increase in twitch force amplitude and the M-wave for two successive increases in current. The supramaximal stimulus intensity is determined by adding 20% to the maximal stimulus intensity.

The increments in stimulation intensity depends on the subject. For obese participants, the increments can be quite high (e.g., 20 mA) and for lean subjects much smaller (e.g., 5 mA). We have used different supramaximal stimulation intensities depending on the peripheral nerve that was stimulated. I think at least 120% should be used. However, stimulating the posterior tibial nerve with high stimulation intensities can activate the peroneal nerve too and will result in a coactivation of the peroneus and tibialis muscle, which might affect the VA results. The same applies when stimulation the peroneal nerve with to high intensities. Therefore, care should be taken when the stimulation is applied at the calf.

The ramp rate will depend on the familiarity of the subject (slower increase for novice), expertise of operator (slower increase for less expertise), and conductance of subject tissues (slower increase when small increments induce large force changes). The step increase can be reduced as a given increment in current yields less increment in M wave or force. We also continue to increase current to check that a true maximum M was obtained, but we vary between 3 - 5 additional stimulations. In studies without an intervention that might substantially alter peripheral (muscle) function, we only use +20% intensity, but when significant alterations in function are likely (e.g. muscle fatigue) we may increase by 30 or 40% to ensure that axons are sufficiently excited (as per Gandevia guidelines).

**12.1 Determination of maximal**	5 mA.

**stimulation intensity – current increase**	20 mA per step.<br>100 mA for quadriceps.<br>Usually, +20 mA per step for large muscle groups, could do 10 mA for smaller muscles.<br>10-20 mA.<br>No current increase is used.<br>20mA per step.<br>50 mA.<br>Depend on the subjects (if familiarized, etc).<br>Depends on site and width of stimulus and participant.<br>Between 10 and 20.<br>20mA -10mA.<br>20 is what we use.<br>10 mA.<br>5-20 mA per step.<br>10-30 mA.<br>5mA.<br>10 mA per step.<br>50 mA.<br>5-10 V.<br>100 mA then +50 mA then finally +20 mA.<br>50 mA.<br>20 mA per step.<br>10 mA.<br>20 mA.<br>Depends on the factors mentioned above.<br>This varies between subjects and experimenters.

**12.2 Determination of maximal stimulation intensity – stopping rule**	No further increase in twitch force amplitude and/or M-wave for 3 successive increases in current (< 5% of increase).
	No increase in triplet amplitude with further increasing current.
	We use the twitch torque for maximal stimulation intensity. For the quadriceps muscle, we start at 100 mA and increase the twitch ramp intensity by 100 mA until torque plateaus or decreases. We then reduce by 50 mA and then use whichever intensity produced the largest torque. For e.g., 100 mA, 200 mA, 300 mA, 400 mA, and 350 mA, if we started seeing the torque plateau or going down from 300 mA to 400 mA. If 350 mA produced the largest torque, we then use that intensity.
	No further increases in M-wave peak-to-peak amplitude and twitch amplitude despite a further increase of 20 mA.
	Plateau in twitch amplitude
	No increase in twitch force amplitude for 3 successive increases in current
	Depending on stim method (pad or nerve) I'll usually ramp up 20-50mv per twitch, I monitor both the M-wave and twitch torque to determine a plateau, then increase current 10-30% and ensure there was not a further increase or decrease in torque.
	No, just asking the subjects if not too uncomfortable
	Depends on site of stimulation e.g., stimulation over biceps/ brachialis can plateau but then start to increase again if higher intensities are used. This later increase can be due to inadvertent stimulation of median nerve bringing in the forearm flexors (which cross the elbow and can provide extra unwanted elbow flexion force).
	Plateau in M-wave amplitudes as well as the size the twitch torque.
	No increase in twitch force for three consecutive steps of 10 mA. Also decrease the current a few steps to confirm the minimum current intensity eliciting a maximum response (accommodate for any potentiation of the doublet response).
	No increase in twitch force amplitude and the maximal M-Wave amplitude
	Peak twitch torque for at least 3 consecutive current increases.
	No further increase M-wave P-P amplitude and twitch amplitude for 2 consecutive increases in current intensity.
	The intensity where unpotentiated single twitch (Qtwunpot) and concomitant compound muscle action potential (M-wave) amplitudes reached their maximal values and started to plateau
	Femoral nerve electrical stimulations intensity is increased until the EMG response of all muscles plateaued (Mmax)
	M wave...when all EMGs from all muscles plateau three successive increases in current and twitch plateaus as well...they may be different.
	No force amplitude increase during 2 successive voltage increments.
	Three consecutive stimulations with no increase in force response. M-wave plateau (usually in VL and VM) is also checked during these stimulations.

	No increase in twitch force. However, also achieving at least 1/3 of MVC force/torque when stimulated with the muscle at rest (https://pubmed.ncbi.nlm.nih.gov/22633).<br>When there is no further increase in both twitch force and M-wave for 2 successive increases in current.<br>Maximal intensity when no significant twitch force and M-wave (vastus lateralis or soleus) increase after 2 successive increments.<br>Both [M-wave peak-to-peak amplitude and twitch amplitude].<br>Both, the M-wave and the twitch should be stable.<br>When M wave and twitch force does not increase for 3-5 additional stimulations (at least one stimulation must be quantitatively lower, indicating a true plateau rather than only a slowing of the increase). Prefer plateau in both twitch force and M wave.
**12.3 Determination of maximal stimulation intensity – supramaximal intensity**	20%.<br>20 mA.<br>0% (we use maximal and not supramaximal for reasons explained above).<br>20% on stimulation intensity eliciting a maximum M-wave peak twitch.<br>20%.<br>10-20% when experiment does not involve fatigue assessment. ~30% when the protocol involves fatigue assessment.<br>20% on stimulation intensity eliciting a maximum peak twitch.<br>10-30% baseline testing 10 seems fine, fatigue or damage we go with 30%.<br>30%.<br>10% to +20% for studies without fatigue.<br>Between 20 and 30%.<br>30% above the minimal intensity for the maximal response.<br>Set at 20% higher to ensure supra-maximal stimulation throughout the test.<br>20%.<br>20%.<br>20%.<br>30%.<br>20% on stimulation intensity eliciting a maximum M wave.

	At least +30% of the stimulation intensity eliciting maximum M-Wave.
	20% above plateau intensity.
	For children, we did not use supramaximal intensities (just maximal). For other studies we were typically > 10% above maximum twitch force intensity.
	20% on stimulation intensity eliciting both max M-wave and peak force.
	50 mA over the stimulation intensity not increasing twitch force further.
	20%.
	20%.
	20%.
	At least 120% (depends on the stimulation location).
	20% M-max is acceptable, but when significant peripheral changes are expected (e.g., fatigue) then +30-40% of M-max are used.
**13. Benchmark of VA values**	> 90% for quadriceps. No experience with other muscles.
	90% of MVC torque.
	Greater than 90%; depends on muscle group.
	~95%.
	I would consider activation to be maximal over 95% for all muscle groups.
	100%.
	100%.
	>90% for all muscle groups.
	~90% for knee extensors.
	I think this is where my approach may differ from others. I don't think VA is anymore useful then gaining some insight into whether subjects are putting in a near maximal effort. I do not progress with the experiment if subjects cannot get above 90% for [knee extensors], [plantar flexors], and 95% for [dorsiflexors]. If below these values, we run a familiarization session and reassess. I use the term "near maximal" for any value above 95%
	>95%
	'Maximal' voluntary activation for an individual is the voluntary activation that is measured during maximal efforts with appropriate encouragement, feedback and that the participant thinks is a good effort. Typical levels of voluntary activation in maximal isometric efforts vary across muscle groups, e.g., voluntary activation above 95% is common for biceps/brachialis, adductor pollicis is more like 90% and for the knee extensors 90% is a high level.

>90 (we consistently observe between 90 and 100% at rest).

In general, voluntary activation may be overestimated using this method, the closer to 100% the better. I would say >95% is pretty maximal.

Depend on settings - but we have mainly used the highest possible for the given condition (and used for "fatigue studies" not training).

It depends essentially of the muscle length. At intermediate muscle length, >85-90% is consider to be maximal.

Knee extensors: ~90%; Plantar flexors: ~90%; Dorsiflexors: ~95%

Activation level is maximal for values very close to 100%, I would say above 95%. No difference between muscle groups is considered to determine if the activation is maximal or not.

Knee extensors: ~85%; Plantar flexors: ~90%; Dorsiflexors: ~90%.

> 85% on knee extensors.

>90% overall.

98%.

Depends on the population, the muscle, and the technique. Quadriceps young-middle age adult (~95% with burst superimposition); Triceps surae young-middle age adult (~95% with burst superimposition); Infraspinatus (>90% with interpolation). Child of typical development--quadriceps or triceps surae (>90% with interpolation).

I have mostly used femoral nerve or quadriceps muscle belly stimulations. If the voluntary force level immediately prior to stimulation is above 90% of MVC, then I consider that an acceptable trial. Thus, even as low as 85% voluntary activation level could be acceptable if other criteria are met. But yes, typically trials above 90% VA are taken forward to further analyses. Largely, the group means are approx. 94-95% VA.

>95%

>=90 % in quadriceps

No benchmark.

For the quadriceps, greater than or equal to 95%.

The activation level strongly depends on the investigated subject. I have investigated sprinters and throwers who had VA's of nearly 100%, while a "normal" sport science student achieves mean activation levels of around 90%. When examining clinical populations, such as knee endoprosthesis patients, we have observed VA values of 60%. Therefore, no general statement can be made.

I have never used a definition of 'maximal' activation, which I would normally consider as >99%. I also note that the maximum activation that might be obtained in an unimpaired, non-fatigued individual may sometimes depend on peripheral factors (e.g., in quadriceps, we've observed lower 'maximal' values of ~92% with knee extended then higher values of ~100% with knee flexed (long muscle length), which we attribute to differences in calcium sensitivity influencing the amount of central drive required to attain 'maximum activation' (see my definition previously). The maximum attained also varies considerably by muscle group (usually between 85-97%), so any definition may need to be muscle specific.

**14. Description of a familiarisation session**	I run a full familiarization, which means to perform exactly what will be done in the experimental trials. Typically 6 pre- and post-exercise MVC + electrical stimulation.<br><br>Submaximal and maximal voluntary contractions without stimulations during contraction (2x 50%, 2x 75%, and 1x 100%). Familiarization for electrical pulses is only provided at rest and not during contraction.<br><br>Our familiarization sessions include both submaximal and maximal voluntary contractions. Participants usually do between 5 and 10 maximal contractions, with at least two of them being without stimulations. Once a participant can produce consistent maximal voluntary contraction (with less than 5% difference in maximal force), we introduce the superimposed stimulations as well as the post contraction stimulations. Maximal and supramaximal stimulation intensities are determined as described above before having the participants realize any contractions.<br><br>At least 5 submaximal contractions with stimulation<br><br>Familiarisation sessions include both submaximal and maximal voluntary contractions with and without stimulation. The number of contractions depends of the ability of the subjects to reproduce consistent MVCs. Usually, a minimum of 3-5 MVCs are needed.<br><br>I start with several voluntary contraction levels (e.g. 30% MVC, 50% MVC, 70% MVC with visual feedback) and give a single stimulus at the plateau of each contraction.<br><br>First, I would include at least 5 sub-maximal contractions at increasing intensities without stimulations. Then I would include 2 maximal voluntary contractions. Then I typically add at least 10 stimulations at rest at increasing intensities, similarly to what is done during a ramp. In the end, I add few submaximal contractions with stimulations.<br><br>Order of MVC attempts and what we do: 1) MVC with torque feedback/encouragement; 2) MVC with torque feedback/encouragement & a marker of previous attempt to beat + ITT; 3) Repeat 2.<br>If not above 90% we repeat, add some dynamic contractions to learn the action. If not above 90%, then we ask them to come back another day.<br><br>Variable.<br><br>A few progressively increasing submaximal efforts. Typically, three to five maximal efforts. Some participants require further practice and instruction so that contractions are performed in the desired fashion. Set-up of the stimulus to ensure that the participant finds it tolerable and application in two to three maximal efforts<br><br>After establishing stimulation intensity, our standard familiarization is: Six submaximal, 2 to 3 s isometric contractions (2 x 25%, 2 x 50% and 2 x 75% of perceived maximal effort). Note: 2-3 MVC's without stimulation and 2-3 MVC's with stimulation (randomized)<br><br>It is important that you respect your participant, but it is also important to not make it too exciting. Keep the description of what is going to happen short, but clearly explain what will happen. I always say: 'In general only a few participants like the stimulation, normal people find it unpleasant, but most of us also get used to it pretty quickly'. In general, the participants have to get used to the same measurements that they will be exposed to in the real experiments.<br><br>Yes, always use a familiarisation trial.<br><br>Ramp of contraction with: 1) Submaximal contractions at different intensities (25, 50, 75, 90% of their estimated maximal force) (between 3 and 5 contractions at each intensity) and maximal voluntary contractions (between 3 and 5); 2) Ramp of electrical stimulation with very low intensity at the

beginning to accustom the participant; 3) MVC with stimulation during contraction and at rest (between 3 and 5 depending on the quality of contractions).

At least 50 submaximal voluntary contractions and 5-10 maximal voluntary contractions with and without (half with, half without) superimposed stimulation and resting stimulation following the contraction.

At least 5 MVC after appropriate warm-up, including at least 2 MVC with stimulations.

Progressive warm-up consisting of: 4 contractions up to ~ 50% MVIC, 4 contractions up to ~ 80% MVIC, and 2 contractions up to ~ 100% MVIC with a rest of 30 s between each contraction. Familiarization with electrical stimulation on the resting muscle and familiarization to perform reproducible MVCs with and without superimposed stimulations.

Familiarization with low-intensity stimulations at rest and during submaximal contractions (n=10).

Stimulations on relaxed muscles, and then at least one stimulation during maximal contraction for familiarisation.

Twitches at rest to a plateau. Submaximal contractions with interpolated twitch at 20% MVC then 50% then 100% effort

Typically two submaximal efforts and one maximal effort without the stimulation. There was typically exposure to submaximal stimulation, if there was not step-intensity procedure. Otherwise, the step-intensity procedure familiarized the participant to the stimulation.

Passive, submaximal electrical stimulations. Submaximal and near-maximal voluntary contractions without stimulation. (<10 trials at incremental force). Near-maximal voluntary contractions with submaximal electrical stimulations (1-2 trials).

Both submaximal and maximal contractions (increasing level), including submaximal and maximal stimulation. Usually assess with MVC torque reaching CV<5% in repeated trials.

Series of MVCs without stimulations until CV < 5%. Then, we add stimulations and should get similar MVCs (CV <5%) in order to validate that they are comfortable with the stimulations and still contract maximally. Therefore, there is no definite number of contractions.

Fast description of the stimulation. Trials with submaximal contraction and very low stimulation to explain the procedure. Then, fast determination of a maximal stimulation intensity with several repetitions in maximal conditions. The volunteer should understand the procedure and timing

Complete ramp protocol as described above so the participants are completely familiarised, and thresholds are known to the research team ahead of the next session.

The familiarization session consists of submaximal and maximal stimulations at rest. Of course, the stimulation will also be applied during MVCs. We have no general rule how many contractions with stimulation will be performed. Instead, we look at the CV of the MVC values, which should be below 5%. Furthermore, we look at the torque-time curve and evaluate if a stable plateau was produced. If both is not the case, additional trials will be performed. Therefore, the number of contractions and stimulation strongly depends on the performance stability of the subjects.

Submaximal 3 - 5 contractions at increasing force levels) and maximal (at least 3 practice efforts) voluntary efforts, then a series of stimulations with increasing intensity to a moderate intensity in relaxed muscle, and maximal efforts with intensities reaching supramaximal. We use the low-moderate intensity stimulations to give an idea of the likely maximal intensity so that we can choose appropriate current steps for use in experimental sessions (some who reach high intensities before evidence of torque-M plateau in familiarisation will be given larger step increases in experimental sessions).

**15.1 Familiarisation session – comments**	It must occur on different days.
	Familiarization session and formal testing should be on different days.
	Depends on group, naivete, etc.
	Familiarization and experimental testing can be conducted on the same day.
	Ideally, I believe it is best to conduct the familiarization session on a different day than the testing day. Yet, due to participant time constraints, I believe that it can be acceptable to conduct the familiarization on the testing day, provided that at least 30 min of rest is provided between the end of the familiarization and the beginning of the testing session. However, in this case, investigators should not pursue with the testing session if signs of too much pain or fear of the stimulation is observed during the familiarization part.
	Familiarization and testing should occur on different days. However, that is not always feasible in a clinical setting.
	I believe that the familiarization can be performed on the same day.
	I use the ITT value to determine whether to continue the study or practice more.
	With appropriate experimental design, familiarisation can be carried out on the same day.
	Familiarisation on a separate day is to be preferred I would think. It also depends on the importance of the outcome. If you merely want to check/ get an indication of voluntary activation, you can first do some MVCs without superimposed stimulation, and just a few additional attempts with stimulation. In this case, a separate familiarisation session would not be necessary.
	Depends - if simple static (e.g., MVC) then 3 trials on same day is fine - but for sustained or our "dynamic model" we use more - https://www.ncbi.nlm.nih.gov/pmc/articles/PMC8283014/
	Preferable to do on a separate day.
	Familiarization should ideally take place on a separate day, 24 hours or more before actual testing.
	Familiarization and formal testing must occur on different days. Frail participants may need more than one familiarization session.
	Familiarisation and testing should occur on different days.
	Necessary.
	Familiarization can be done on the same day.
	Familiarization and the test session should be on different (non-consecutive) days. Whether 1 familiarization session is sufficient has not been explicitly determined to my knowledge, but constraints in timetabling and the wish to reduce the total number of stimulations prevent more familiarization sessions.
	Ideally, different days. If not possible, due to e.g., schedules, then considerable rest (minimum 30 minutes) between familiarisation and actual testing must be provided.
	The familiarization session should be conducted on a sperate day.

	We explain why some discomfort is felt, but that it should not be very 'painful'. Usually, an understanding of the physiology of the response helps to reduce stress and improve reliability of outcomes.
**16.1 Level of acceptable reliability for familiarisation – comments**	< 2%<br><br>CV < 5%<br><br>ICC: >80%<br><br><5%<br><br>I do not think that reliability of familiarisation is a useful concept at all!<br><br>< 10%<br><br><5%<br><br>That subjects can complete the contraction without "disturbance from the stim"<br><br>CV < 5% at the end.<br><br>CV of 3-5%<br><br>Same answer as previously, difficult to put numbers here I think.<br><br><5%<br><br>CV<5%<br><br>CV <5%<br><br>I have not assessed this. We have looked for at least one acceptable trial fulfilling all criteria. Variation between trials can be very large inherent to the complexities of the method.<br><br>CV < 5%.<br><br>CV =< 5%.<br><br><5%.<br><br>< 5% for MVCs.<br><br>We aim for CV <5%, but <3% is a better target.
**17.1 Number of contractions completed – other**	At least 3, but 6 is desirable.

	Depends on 'learning', and group.<br><br>two to three with a CV<5%.<br><br>I use 5 submaximal and 1 maximal contraction and use linear interpolation to obtain the VA.<br><br>It is not so much about the number; the timing of the superimposed pulses and the level of voluntary force are much more important: voluntary force has to be stable and as high as possible. Activation calculated from a good (stable force) attempt with the highest voluntary force, is more reliable than activation calculated from a contraction with lower voluntary force (and/or fluctuation voluntary force levels).<br><br>3-5 maximal attempts. It will depend greatly on the population and physiological/injury/surgical state.<br><br>CV<5%.<br><br>The number of contractions depends on the subject (described above). We consider a CV of <5% for MVCs as appropriate.
**18. Instructions provided to participants**	To contract as hard and fast as possible, and sustain as hard as possible for 5 s.<br><br>"I would like you to push as hard as possible". Every time plot the subject's highest MVC torque on the screen and push the subject to go over that line while loudly encouraging the subject during each attempt.<br><br>Strong verbal encouragement and visual feedback and appropriate rest between attempts.<br><br>We provide real-time feedback of their torque curves and provide target lines on the monitor, and we instruct the participants to contract as hard as possible and to attempt to exceed the target lines on the monitor placed in front of them.<br><br>Contract as hard as you can in order to achieve your maximal force with 1-2s and then maintain this contraction for 2-3s.<br><br>As hard and as fast as possible<br><br>The instruction is to contract the muscle group as hard as possible and to try to keep the same level of contraction even during the electrical stimulus.<br><br>Contract as hard as possible. Additionally, I give loud verbal encouragement during the contraction.<br><br>The line on the screen reports the force that you are exerting. I would like you to push (or pull) as hard as possible progressively reaching the maximum and then keep pushing as hard as possible for 5 s.<br><br>Contract as hard and fast as possible, try to beat the line.<br><br>As hard as possible, fast but not explosive contraction (i.e., not a kick)<br><br>General instructions: 'I want you to pull absolutely as hard as you can, to get to the top of your force as fast as you can and to keep pulling until I say relax.'<br>Each individual effort: 'Get ready to make a maximal effort. Remember - pull up hard and fast and get as many lights as you can [try to make the line go off the top of the screen]. 3, 2, 1 pull, pull, pull, pull, pull, pull, pull.'<br><br>When we are looking only at maximal force, our instructions are: "contract as hard as fast as possible and maintain your effort for 3 to 4 seconds"

The force has to be stable. The instruction is push as hard as possible and within a few seconds to maximal force levels (too gradual an increase doesn't work). First, do some MVCs. We always do that with a line on the screen showing the highest attempt thus far. 'Now, try to reach that line again, higher or a bit lower is fine, but keep on pushing and on the plateau phase an electrical pulse will be given. Just keep pushing maximally until we instruct you to relax.'

Different from MVC to dynamic...

Contract as hard as possible. You contract and relax when the experimenter tells you.

Contract as hard as possible with a progressive force build-up during the first 1-2 s. Visual feedback provided as a torque-time trace. Systematic and strong verbal encouragement provided.

Progressive increase in force within 1-2 sec to reach MVC and then maintain maximal force for 3 sec. Ignoe the stimulation and continue to contract maximally until I tell you to stop.

I would like you to “contract as strongly as possible” during each MVIC. I strongly encouraged them during each maximal effort.

Contract as hard as possible in less than 3 s.

Reach maximal force level within 1 s and maintain maximal effort for at least 2 s

Contract as hard as you can for 2-3 seconds. Then I ask the subject after they have performed if that was a maximal effort.

"When I say "go" I want you to (kick, press, or rotate) as hard as possible and try to go higher than the force target that appears on the screen."

We perform MVCs without stimulation first (2 trials) to establish target force levels. The participant is instructed to contract hard to achieve the target line or even exceed this. the target line is 95% of MVC from the previous non-stimulation trials.

'Contract as fast as possible and keep pushing hard until you hear me asking you to stop'. Assessment of maximum force reached through plateau on torque.

Participants are instructed to contract as hard as possible and to maintain their effort throughout the contraction.

"Contract as hard as you as can when I say go. And relax when I say stop." As fast as you can is not useful for this evaluation and can bias the results (force fluctuations before the stimulation)

Contract as fast and hard as possible whilst pushing through the stimulus.

The instructions depend on the aim of the study. If rate of torque development should be analyzed too, the instruction “as hard and fast as possible” seems appropriate. In other cases, “as hard as possible” seems good.

We tend to use 'as hard and fast as possible' to indicate the 'maximality' of attempt (i.e., we include 'fast'); note that 'hard' always precedes 'fast' in the instruction as it's the primary objective. We then give loud verbal encouragement to produce greater force during the trial, show the subject their result, and ask that they beat it on the next attempt. If the 3rd attempt is >5% greater than others, a 4th attempt and 5th attempt may be given. We don't allow more than 5 attempts (in fact, I don't remember having to give 5 attempts).

**19.1.1 Provision of feedback - timing - other comments**	Both [real time and during rest], encouraging them during the contraction, but also explain and motivate during the rest periods<br><br>Both real time and during rest periods.<br><br>Verbal feedback to clearly indicate the stimulation timing and contraction timing. Feedback to encourage volunteers. Visual feedback is not mandatory.<br><br>Both, real time and during the rest periods.<br><br>They can see their effort in real time, but usually don't focus on it because their capacity to comprehend this output is reduced during an absolute maximal effort. We regard rest-time feedback as the key.
**20. Number of contractions used for** a**nalysis - comments**	Depends.<br><br>Linear interpolation of 5 sub and 1 maximal contraction.<br><br>We generally take the best initial contraction during baseline testing, however as our post-fatigue measures have been immediate we have only been able to take one (which is a limitation).<br><br>I have typically used peak performance, but you could make good arguments for average performance.<br><br>Depends on the procedure and study rationale. If time, could be the average of several. If exploration of a transient phenomenon a single contraction can be used.<br><br>We use the 'maximum', but the research question might also ask for the variability in response, or average response (e.g. in ageing, a maximal outcome is possible, but it may occur less often, so the mean may provide a better description of 'normal' activation).
**23 Application of stimulus.**	The stimulus is triggered manually when voluntary force reaches an obvious plateau.<br><br>Manual trigger.<br><br>Several manual or programmed stimulii during the 3-5s MVC.<br><br>We have published an automated torque-based triggering approach (PMID: 19533648) and use this approach. If manually triggered because of the conditions not met, the trigger will be provided if the participant couldn't reach the target until 2-3 seconds. However, we rarely do this and usually, these trials are discarded as they don't end up being the peak trial.<br><br>Manually delivering the stimulation based on the real-time force recording trace to ensure the stimulation is delivered on the plateau.<br><br>Peak force reaches a plateau. Manual stimulation.<br><br>I only used manual trigger.<br><br>I will manually trigger the stimulus if the participant has reached an obvious plateau. If they have not reached an obvious plateau, contraction is repeated.

As I ask to progressively reach the maximum force and to keep it maximum, I manually apply the stimulus as soon as I see the force reaching an obvious plateau (thus short after the force transient). I'm not sure is optimal but it works well to me.

By eye, manually.

Manual.

Manual trigger

We have used both in the past, but as our technique has evolved, we've gone to an automated approach to eliminate subjectivity.

Manually trigger the stimulation when the participant reaches a plateau.

We have used both manual and triggered (essential in the dynamic model).

Manually triggered when the participant is on the force plateau.

Manually, as close as possible to the MVC plateau. If this criterion is not met, the trial is excluded or in case of a small decline the formula with the correction factor proposed by Strojnik and Komi (J Appl Physiol 1998).

Manual delivery of the stimulation over the plateau of the MVC.

Not used.

Stimulation is triggered manually at the torque plateau of MVC.

Manually trigger when force reaches a plateau and level close to a previous MVC without stimulation.

The trigger is done by the investigator who elicits the stimulus when 1-2 seconds after the contractions has started.

Optimally you would use a real-time moving window average at an appropriate time interval, which may be muscle dependent.

We use manual triggering only during isometric trials.

Optimal condition would be automatic triggering of the stimulus when reaching at least MVC torque (identified prior to the stimulation trial) and maintaining for at least 300ms

The stimulus is triggered manually, in general during the 1st second of contract, but sometimes later, depending on the individual pattern of contraction.

Constant timing

The stimuli should be applied at a specific joint angle, where the maximal force/torque is produced. This should be done automatically and NOT manually.

Always use manual stimulus generation after 'plateau' reached for at least 1 second, although automated methods are possible (but complex to use accurately). Contractions are repeated if that plateau is <95% of the true maximum or if post-hoc observation indicates that the stimulus was presented with visually rising or falling force (force must be constant at point of stimulation).

**27.1. Stimulation of antagonists – checking**	EMG signal from antagonist.<br>With EMG of the antagonists.<br>This is precisely the reason we do not use supramaximal stimulation, as the evoked torque at rest is contaminated to a greater extent by the antagonistic muscle stimulation than when using maximal stimulation. We also ensure that the stimulation electrodes are not closer to the antagonistic muscles.<br>Measurements of M-wave peak-to-peak amplitude of agonist and antagonist. A reduction of peak twitch combined with an increase in antagonist M-waves would be considered a sign of antagonist activation.<br>EMG activity.<br>EMG activity and sometime distortion in the twitch time course (tibialis anterior).<br>Nonlinearity of the twitch size/ contraction level relationship provides an indication of antagonist stimulation.<br>I qualitatively check the raw EMG signal recorder from the antagonists.<br>I watch the torque trace closely when adjusting current to minimize antagonist activation.<br>In some circumstances, record M-waves from antagonist muscles. Check the shape of the resting twitch. Be aware that if during stimulus set-up the twitch amplitude reaches a peak and then starts to decrease with increasing intensity then some antagonist stimulation is occurring.<br>Honestly, we have not - perhaps we should!<br>We tried to do this with surface EMG on the antagonist muscles but is I doubt whether that is very reliable.<br>Typically not checked - assume similar when similar procedure/placement etc. is done and minimal influence (as maximal activation supresses antagonists)<br>Not checked.<br>Stable baseline torque signal when the stimulation is applied at rest.<br>Peak twitch should not decrease when applying supramaximal intensity. M-wave amplitude on the antagonist muscle can be recorded.<br>With EMG.<br>EMG of antagonists.<br>Use of EMG on the antagonist where possible.<br>I have used EMG for some populations, but clinical it is not time feasible.<br>EMG response (M-wave) of antagonist muscle.<br>Using EMG.<br>Using EMG (M-waves).

	Studying antagonist EMG activity.
	I monitor the M-wave of the antagonist and try to keep it small.
	EMG amplitude is monitored. In some cases, we've searched for NIRS signal.
**27.2. Stimulation of antagonists – recommendations**	EMG signal increases less than 5% of baseline.
	Using maximal stimulation instead of supramaximal stimulation. Careful positioning of stimulation electrodes. Instructing the participant to relax and deep breath while providing resting stimulation to avoid voluntary co-contraction of the antagonistic muscles due to discomfort.
	Smaller stimulating electrodes.
	Do not use very large stimulus intensities. Antagonist stimulation impairs the validity of the VA estimate much more than submaximal agonist stimulation!
	Beyond preferring peripheral nerve stimulation I don't know what to suggest.
	Carefully ramping up current.
	Use lower rather than higher "supramaximal" intensities to minimise current spread. Select nerve or muscle stimulation as appropriate for different muscle groups. Choose muscle groups or set ups where the agonist is strong and the antagonist weak. Be suspicious if participant repeatedly achieves 100% voluntary activation. If antagonist stimulation is suspected, assess superimposed twitches in a deliberate 90% maximal contraction - a lack of superimposed twitch in a strong submaximal effort indicates a problem with stimulation.
	Use a reasonable supramaximal intensity of stimulation.
	Careful localization of the position over the nerve trunk or the muscle belly (motor point) where the stimulation is more effective. Avoid excessively high current intensities.
	Using muscle stimulation over nerve stimulation in some instances. Using electrodes of small size.
	Sometimes I have observed that supramaximal stimulation during the intensity-ramp procedure actually lowers the force. I had presumed that it was likely antagonist activation.
	Estimate antagonistic activation and adjust the agonist outcome accordingly.
	Not really an issue when stimulating the femoral nerve for quadriceps VA.
	Monitoring of the antagonist M-wave, which should be kept as small as possible.
	Observe antagonist EMG as current intensity is increased in pre-testing protocols. Move anode if crosstalk observed. In rare cases, a subject may not be suitable for study.
	Central fatigue.

**28.1 Inferences from twitch interpolation - other**	A lack of voluntary activation reduction following a fatiguing task must be interpreted with caution as peripheral fatigue may lead to an apparent increase in voluntary activation level. However, a reduction in voluntary activation level following a fatiguing task can confidently be interpreted as a reduced ability to fully activate a muscle.<br>It's a peripheral measure to try and assess central processes. I have shown an over- and under-estimate of VA simply based on MTU length. Therefore, I do not put a lot of weight on VA other than using it to determine whether subjects can contract near maximally.<br>Reduced voluntary activation measured with peripheral stimulation implies that some motor units (i.e., motoneurons) are not recruited or are not firing fast enough to produce tetanic contractions of their muscle fibres. Because the measure is about how well the muscle in its current state is driven by neural drive, changes in muscle fibre properties can theoretically alter voluntary activation without a change in neural drive.<br>I would be hesitant to over-interpret VA using the ITT.<br>The participant is unable to make use of the maximal force-generating capacity of the muscle. Decreases in voluntary activation (e.g., with fatigue) indicate that some central aspects in activation are compromised.<br>We typically compare pre-post (or during) and compare to e.g. rest, not as "absolute measure" or comparison across participants.<br>I don't see the link between a deficit of voluntary activation - that is common after joint injury/surgery - and is usually defined as (voluntary) "activation failure" and a reduction in voluntary activation induced by fatiguing exercise - which is commonly associated to the occurrence of "central fatigue".<br>There can be peripheral contamination for central fatigue quantification. Also, I like the idea of a 'semi-quantitative' method as a value of voluntary activation per se does not mean much but the variation after an intervention / comparison between 2 populations is more meaningful.<br>Presence of central fatigue.
**29.5. Methodological limitations – other**	As long as the effect size between groups (or after the intervention) is very large, the validity of this method is preserved.<br>If people use VA as a "ballpark" measure (greater than 90%), and not get caught up in minor changes 93 vs 96%, I think it's a useful tool to help ensure near maximal activation.<br>There may be a rather large learning effect. To get the best indication of voluntary activation more sessions may be necessary.<br>IMO, the main limitation of this technique is the discomfort associated to the post-MVC stimulation (evoking the resting doublet), which preclude its application to all populations (and more particularly so for patients).<br>Role of peripheral contamination in the measurement: major limitation.<br>Whilst doublet stimulations are common, the MTU is a low-pass filter and sometimes a longer duration stimulation may be optimum (e.g., triplet). The method can only tell us whether we are able to fully recruit the muscle under the current conditions; in some conditions a small loss of actual central drive may have no detectible effect on %VA (e.g., in some muscles at long length when calcium sensitivity is high and few cross-bridge attachments can be made).

**30. Populations where the measure provides a meaningful assessment of voluntary activation – other comments**	The validity is based on the questions posed. For example, if the question is to compare the % voluntary activation after intervention in highly resistance-trained athletes who are already at near 100% activation prior to the intervention, then the method may not be sensitive. However, if the question is to compare an untrained with a trained population, then it is valid.<br><br>Orthopaedic patients (e.g., knee OA; ACL rupture before and after interventions).<br><br>People with other neurological or psychiatric disorders (e.g., spinal cord injury, multiple sclerosis, Parkinson's disease, depression). Any condition where it is desirable to know whether weakness or fatigue are from impairment of the muscle or impairment of neural drive to the muscle.<br><br>Children and adolescents.<br><br>Patients with chronic cardiorespiratory and metabolic diseases.<br><br>I haven't used the technique in some of these populations, so I can't comment. Certainly, it might be most useful in people who are very used to producing maximal contractions and are used to painful stimuli during contraction (e.g. weight trainers and athletes who produce maximal efforts to point of pain). I see no reason that age should be a considerable factor, although reduced limb mass (including fat mass) may increase risk of antagonist activation by reducing distance between adjacent nerves. I haven't used the technique in some clinical populations and haven't had time to consider the possible implications of impairments on the use of %VA.
**31. Settings where twitch interpolation provides a useful assessment of voluntary activation – other comments**	It is not clear that measurement of voluntary activation in an individual patient (e.g., post-injury or post-surgery) is helpful for the clinical treatment of that patient, particularly where contraction causes pain. However, if weakness persists chronically then it may be helpful to know whether rehabilitation should target the muscle or the nervous system.<br><br>We only use for athletic and active/healthy.<br><br>In the context of various chronic diseases.<br><br>To studying neuromuscular changes in environmentally challenging settings.<br><br>Whenever the VA is relevant to the research question.<br><br>However, if the additional pain affects 'effort', the result may be in some ways misleading, although it will still give an impression of the capacity to activate 'under those conditions'.
**32. When should the application of electrical nerve/muscle stimulations for voluntary activation calculation be discouraged?**	In patients who are not able to perform maximal force.<br><br>The ability to maximally drive muscles is usually overestimated and the level of overestimation increases with lower activation capacity. Accordingly, in the case of certain patient populations with lower activation capacity (compared with controls) the difference in ability to assess the muscles' potential between patients and controls will be even greater than calculated<br><br>Individuals who experience a large discomfort as it can affect the validity due to the inability to contract maximally because of pain/discomfort. Individuals with recent fractures or surgery.

	In participants who badly tolerate the discomfort associated with stimulation.<br><br>To determine the contribution VA in neuromuscular fatigue when VA is not tested immediately after the end of the fatiguing task.<br><br>Unmotivated subjects<br><br>Discomfort<br><br>If strong contractions are likely to result in musculoskeletal injury then it is not appropriate to measure voluntary activation.<br><br>Whenever any participant is uncomfortable with the method. Sometimes participants do not want to quit, but when they do not sufficiently habituate it is unethical to pursue and the outcome will be very unreliable anyway. I would be very reluctant in using this technique in cases of muscle/tendon injury recoveries/post-surgery<br><br>When people have a great proportion of fat mass (e.g., obese patient).<br><br>Joint/muscle pain greater than 3 on a 0-10 VAS.<br><br>More than 2 min after the end of an exercise to detect central fatigue<br><br>Patients<br><br>When the muscle is inappropriate<br><br>Pacemaker. Metal in the limb of interest. Fear of stimulation / not obtaining >90% of non-stimulation MVC. Unable to maintain stable (maximum) force plateau.<br><br>When people are scared by the stimulation, then these participants should be excluded of the study.<br><br>When a participant is incapable of producing a maximal isometric contraction or is too disturbed by the stimulus.<br><br>In subjects with a high pain sensitivity.<br><br>Broken or inflamed skin, metal plates, some neurological disorders. Any individual without medical consent for other reasons.
**33.1. Analysis of voluntary force – software used**	Excel.<br><br>Self-written.<br><br>Custom-written software.<br><br>AcqKnowledge, BIOPAC Systems.<br><br>BioPac AcqKnowledge system.<br><br>AcqKnowledge data analysis for Biopac System Inc. Custom script for CED.<br><br>Custom-made software.

	MATLAB.
	Labchart.
	Labchart.
	Spike2 or Signal software from Cambridge Electronic Designs.
	Powerlab; Labchart.
	MATLAB
	Recordings were sampled at 1 kHz (Model 615, Tedea-Huntleigh Electronics, United Kingdom) and processed offline using customized programs written with LabChart software (version 8.1.9, National Instruments, Austin, Texas, United States).
	Powerlab or Biopac.
	AcqKnowledge (Biopac).
	Acknowledge (Biopac).
	Labchart.
	Biopac system.
	CED Spike2.
	LabView.
	Signal 4.14 or Spike software (Cambridge electronic design).
	MS Excel.
	Recording: Labchart. Analysis: MATLAB.
	Acqknowledge (Biopac). Tida (heka).
	Spike2, CED.
	Custom-built LABVIEW based program (Imago, Pfitec, Germany).
	LabChart or Spike/Signal - useful to get real-time capture of M wave to ensure stimulus remains sufficient and of pre-stimulation maximum force to ensure voluntary attempt is valid.
**33.2. Analysis of voluntary force – software used**	Manual.<br><br>Manual.<br><br>Automatic methods using custom-written software.

Manual.

Manual.

Manual.

Automatic force analysis

automatic

Manual.

Both manual and automatic.

Semi-automated - software locates peak within a designated region of a signal - researcher checks that software detected peak is appropriate - average force over a period about that peak is calculated (period of average, e.g. over 100-500 ms, can vary depending on the research question) - baseline force is subtracted from calculated peak force

Manual.

Automatic (objective) methods, but first manually indicate the region of interest (e.g., 1 s before pulse application to 1 second after)

Manual.

Automatic methods

Manual.

Manual.

Manual.

Manual.

automatic

Manually moved cursors in LabView to identify the time period for force to be analyzed. Peak voluntary force was calculated between cursor data points. We used a trigger stimulus to identify the timing of the superimposed stimulus event and calculated the stimulus augmented force from the trigger-event marker until about 500 ms post event marker to allow for electromechanical delay.

Manually determined without prior filtering.

Manual.

Automatic method in Matlab.

Manual.

Automatic detection of the maximum.

	We have used both. Automatic methods are usually manually checked.
**33.3. Analysis of voluntary force – peak (average or single)**	Average over 250-ms window immediately before stimulus.<br>Average over 1000 ms of the plateau of the force signal.<br>Singe point.<br>Maximal value.<br>Single.<br>Usually, I use an average of force over a 0.5-1s period.<br>Single discrete point.<br>0.25 s average.<br>Usually, 500ms avg about the peak.<br>Single (but filtering before).<br>Period of average, e.g., over 100-500 ms, can vary depending on the research question.<br>Calculated as the highest torque value throughout the entire MVC.<br>500 ms average value.<br>The first derivative of the force signal (Figure 2D) was computed to obtain the peak rate of evoked-twitch force development (highest value; RFD) and relaxation (lowest value; RFR). To compare EMD, RFD, and RFR between subjects during the dynamic target exercise intensities and recovery, the data were normalized to a mean value during unloaded exercise.<br>Single discrete<br>I use both options [single and average], depending on the study question. For average I use a time period ranging from 50 to 500 ms.<br>Peak to peak value (single point)<br>Single discrete point.<br>Single discrete point.<br>Average over 0.5 second.<br>Peak performance is peak performance - used a discrete point for the peak.<br>A single discrete point.<br>Single discrete.

	Single discrete point.
	Voluntary force before the stimulation OR estimation of force at the same time point (dynamic setting).
	Peak.
	Average around the maximum (e.g., 100 ms).
	If the signal is filtered/smoothed, then a discrete point can be used.
**33.4. Analysis of voluntary force – difference between automatic and manual determination of peak force**	I prefer to spend more time doing it manually to avoid erroneous identification of peak force.
	Automatic methods save time substantially and remove subjectivity.
	The automatic methods should be checked a posteriori.
	I always want to see the data.
	To get the best of both a combination may be better, of course, an automatized method resulting in errors isn't a good method. However, automizing part of the procedure will make it more objective.
	Manual method - Advantage: checking the torque trace; Disadvantage: time consuming.<br>Automatic method - Advantage: rapid; Disadvantage: can lead to error if there is an issue in the torque trace.
	I always prefer to verify the torque signal, together with the notes I wrote during the acquisition.
	I only use manual determination of voluntary force to keep control.
	Automatic methods save time, but may result in an erroneous identification of peak force.
	Automatic methods may result in errors.
	Visual data inspection is a crucial step during analyses. Therefore, analysing trials manually adds little additional work.
	Automatic methods (assuming both criteria of at least MVC torque and maintained for more than >300ms are met) are more repeatable and based on robust criteria, applied every time and also save participants undergoing stimulus delivery for trials that won't be used. The issue is availability of software available to do that e.g., MATLAB and coding skills.
	Automatic methods are nice because they are objective. However, systematic visual inspection is required to make sure that the detection was OK... We therefore use a semi-automatic methodology!
	In our software, the automatic detection of the maximum occurs together with the visibility of the torque-time curve. So, both aspects are checked.
	Automatic can save time but need to ensure peak force is correctly identified - software allows 'captured' traces to be quickly scrolled to check the point appears to be visually correct.

Article Details:
Osborne, John O.[1*]; Tallent J, Girard O, Marshall P, Kidgell D, Buhmann R. Neuromuscular electrical stimulation during maximal voluntary contraction: a Delphi survey with expert consensus. *European Journal of Applied Physiology*.

*Corresponding Author

Dr. John O. Osborne
School of Sport Sciences, UiT The Arctic University of Norway, Tromsø, Norway.
Address: Medisin- og helsebygget, UiT, Tromsø, Norway, 9037.
ORCID: 0000-0001-8681-8521
E-mail: john.osborne@uqconnect.edu.au
