# Supplementary material for: Neuromuscular electrical stimulation during maximal voluntary contraction: a Delphi survey with expert consensus
Source: Eur J Appl Physiol. 2023 May 29;123(10):2203–12. doi: 10.1007/s00421-023-05232-1 (PMC10492693; doi:10.1007/s00421-023-05232-1)
Supplement: Supplementary file 6 — Supplementary file6 (PDF 311 KB) [file 421_2023_5232_MOESM6_ESM.pdf]

Supplement 6- Free text analysis results

Question 10 Round 1

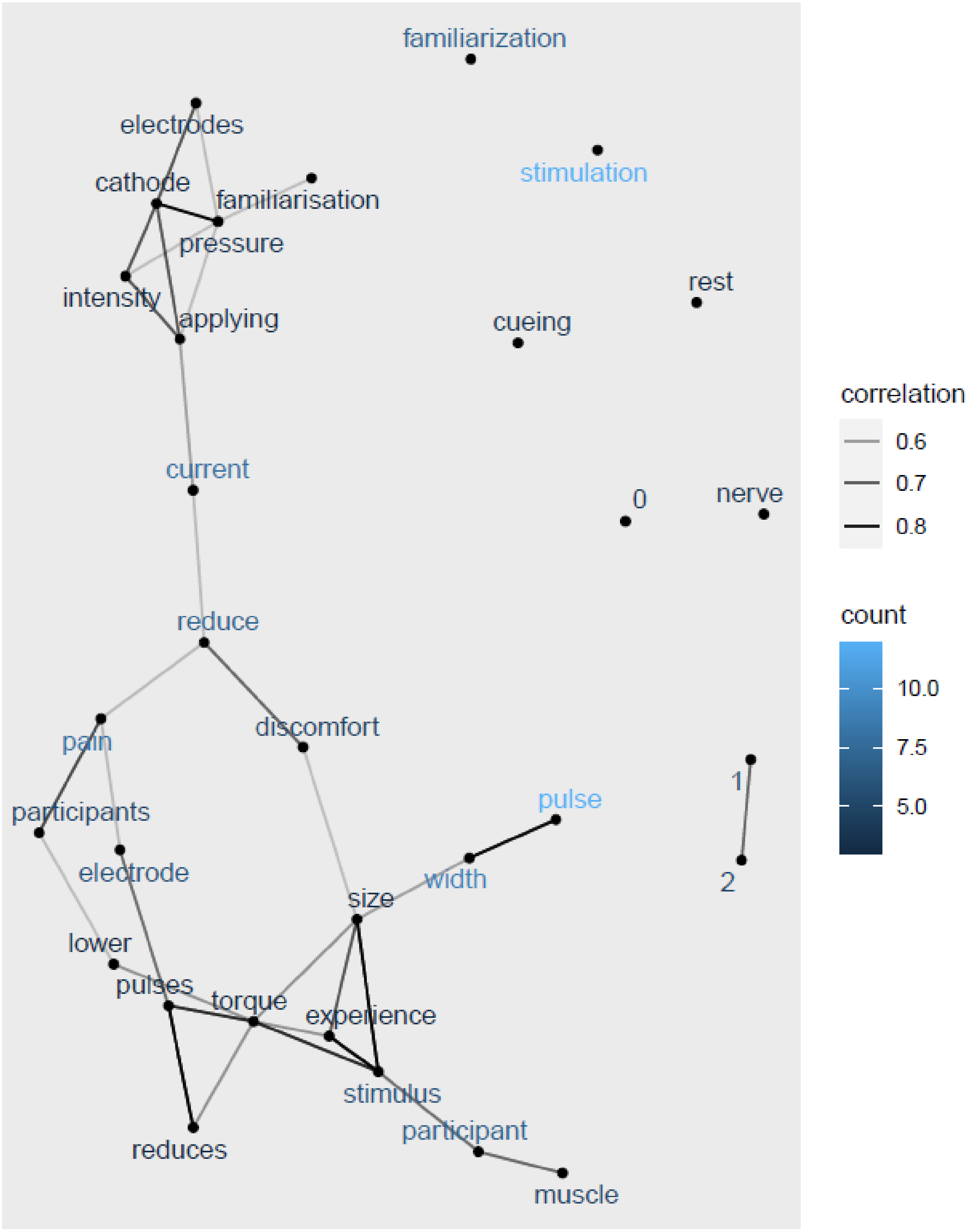

Question 14 Round 1

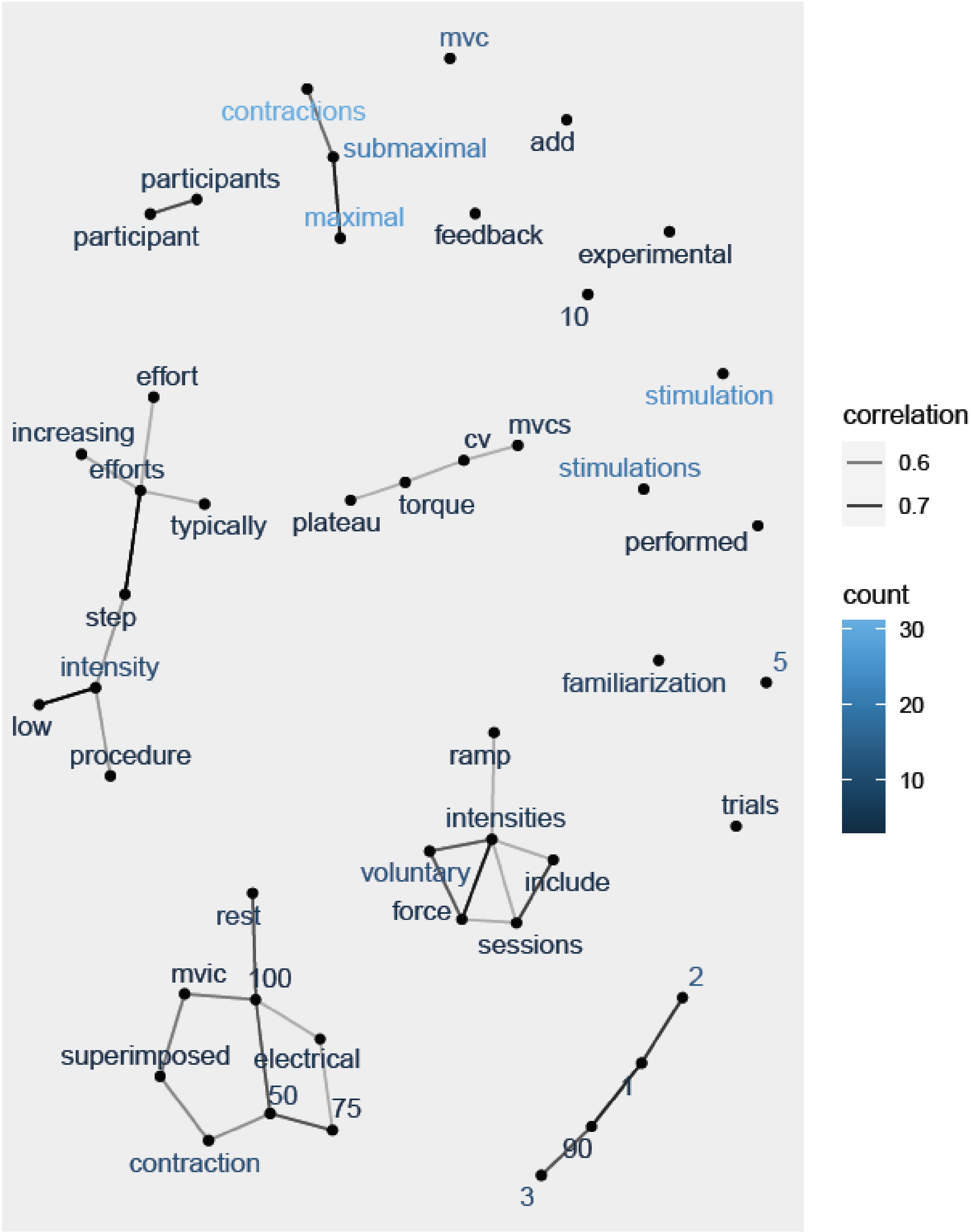

Question 18 Round 1

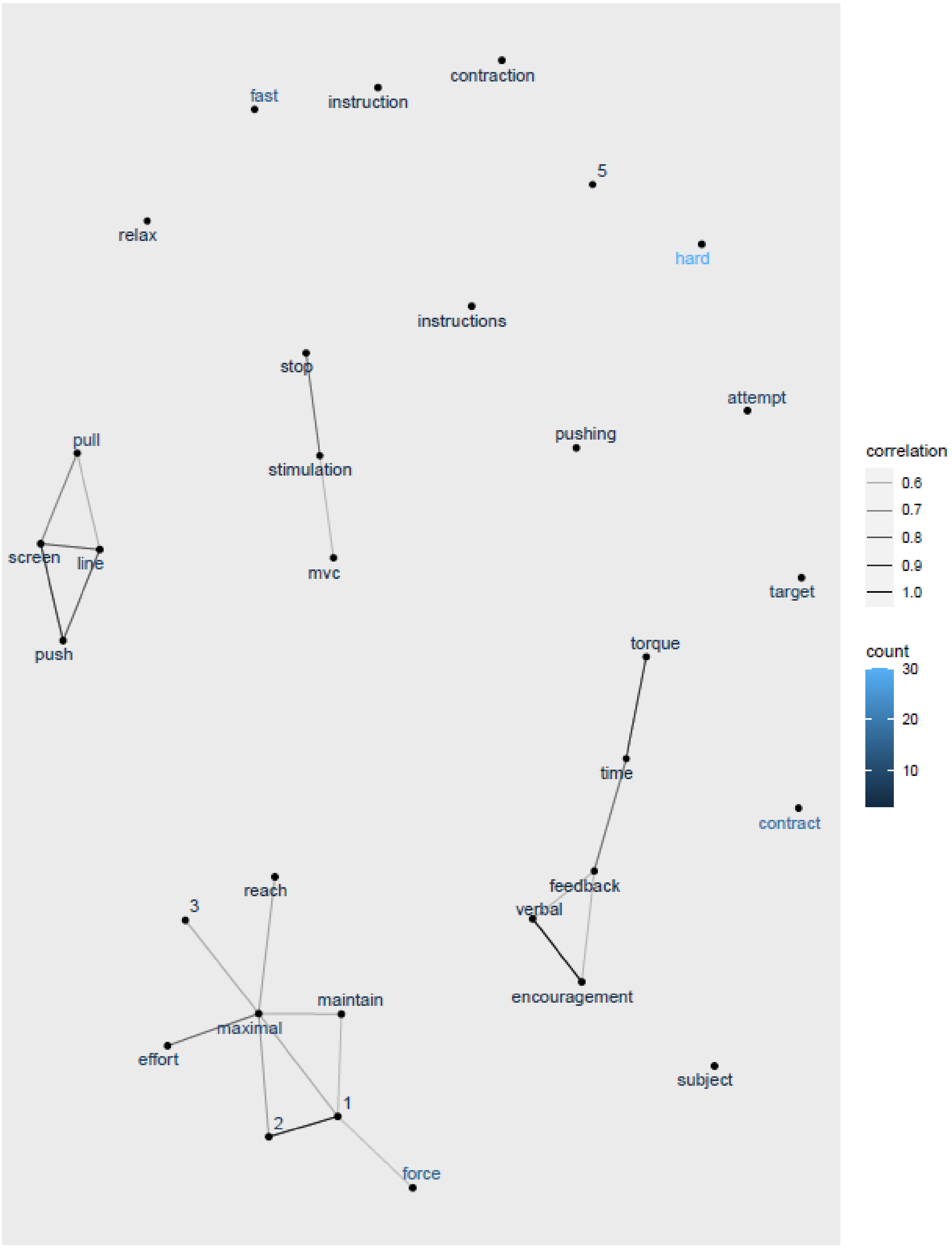

Question 23 Round 1

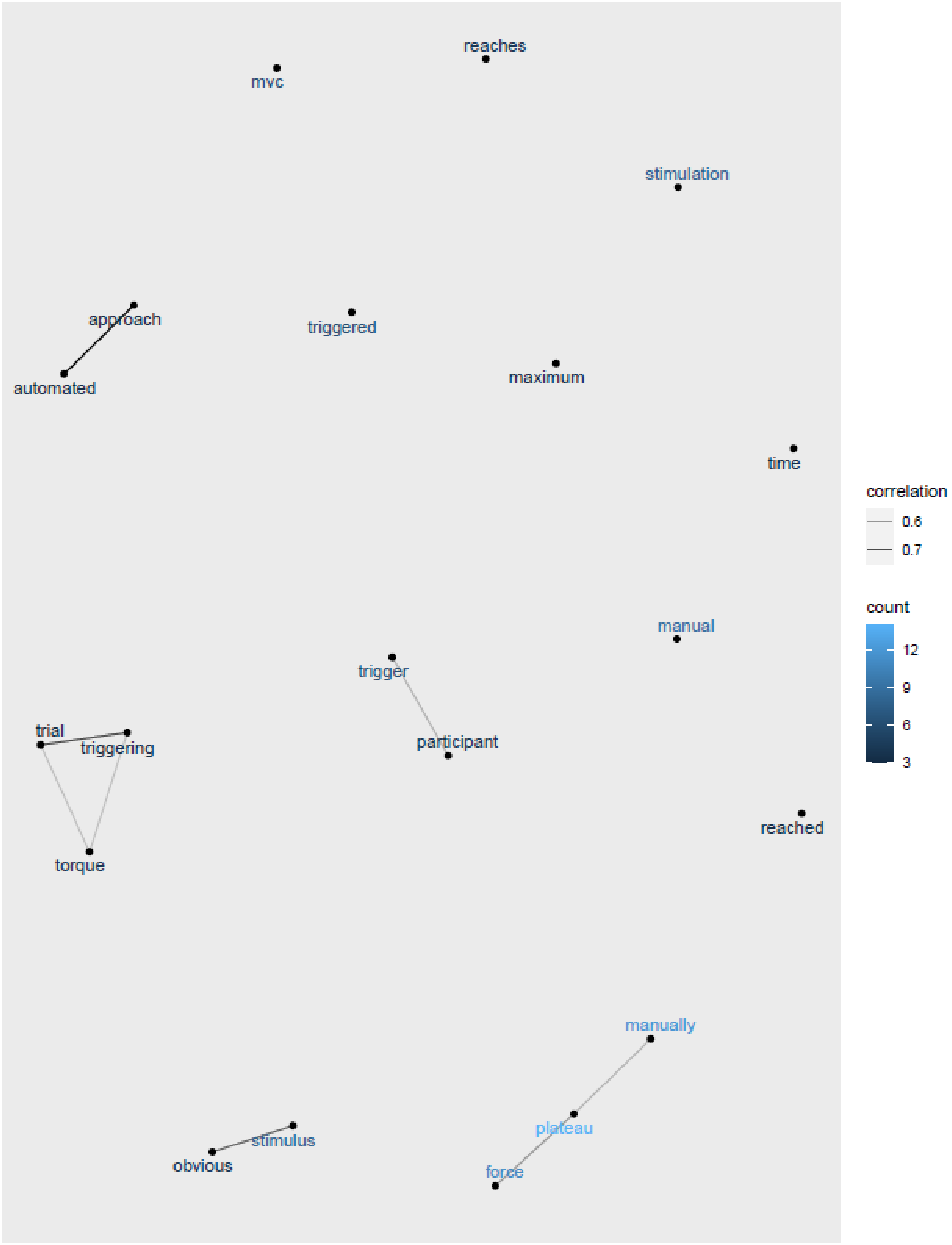

Question 27.1 Round 1

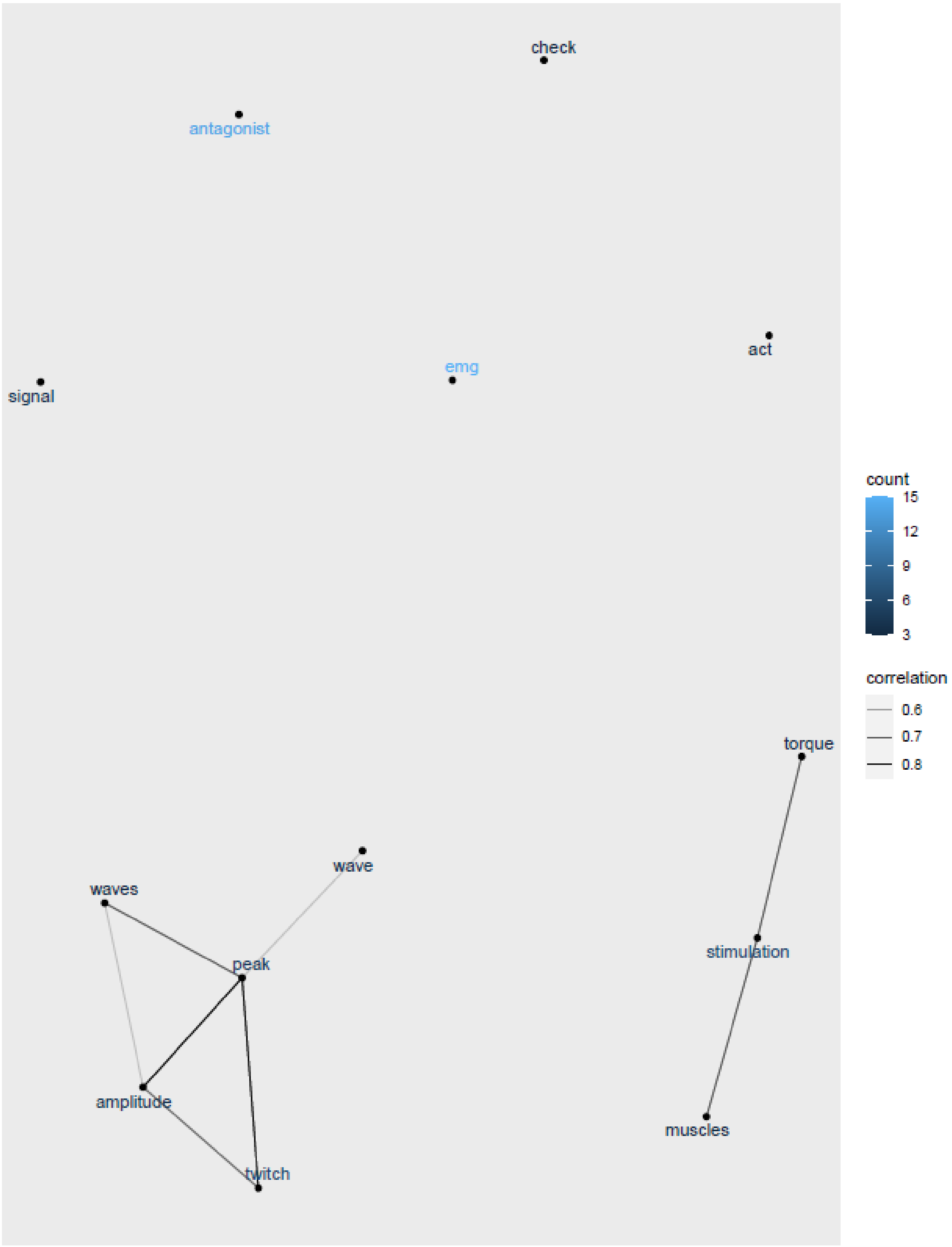

Question 27.2 Round 1

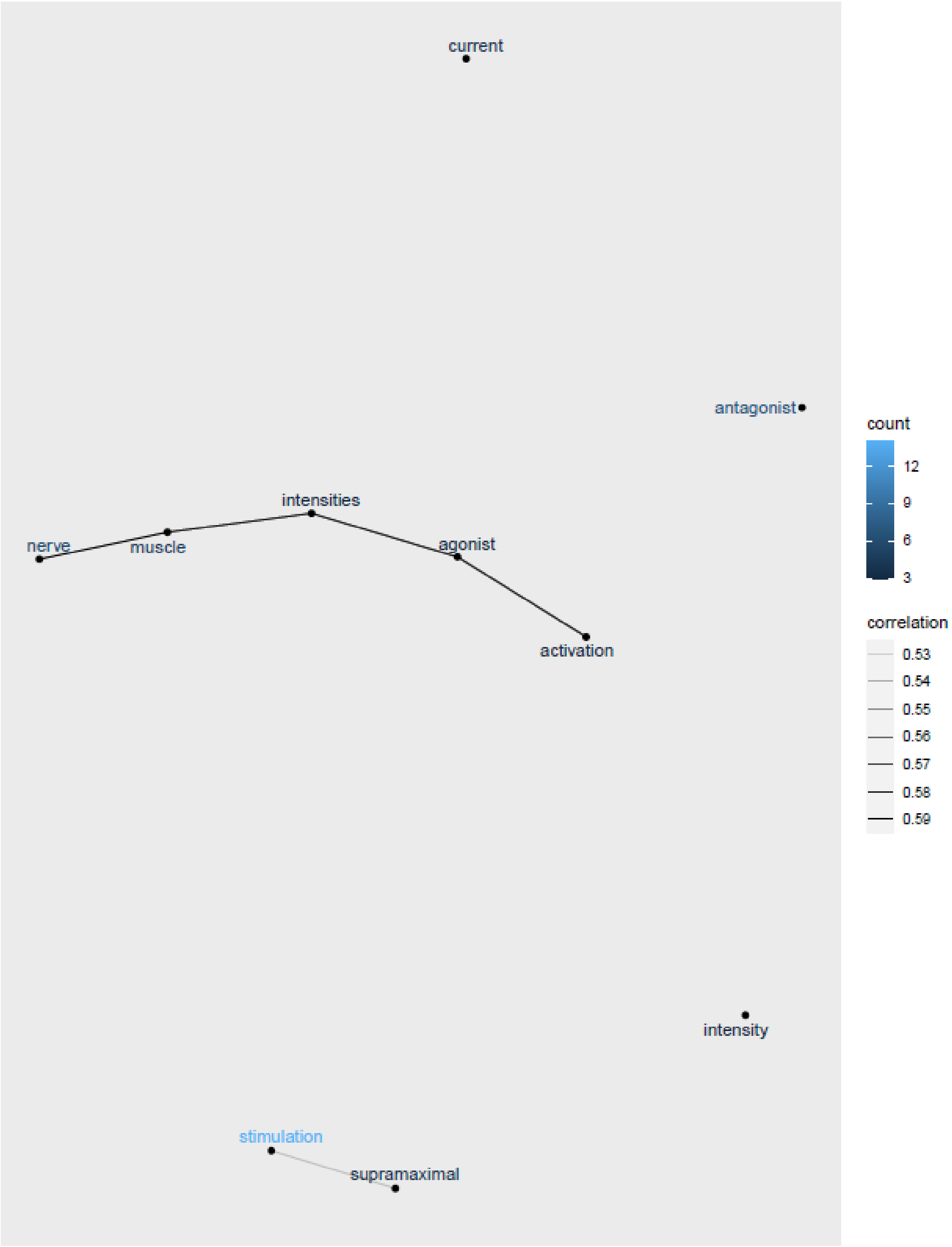

Article Details:

Osborne, John O.[1*]; Tallent J, Girard O, Marshall P, Kidgell D, Buhmann R. Neuromuscular electrical stimulation during maximal voluntary contraction: a Delphi survey with expert consensus. *European Journal of Applied Physiology*.

*Corresponding Author
Dr. John O. Osborne
School of Sport Sciences, UiT The Arctic University of Norway, Tromsø, Norway.
Address: Medisin- og helsebygget, UiT, Tromsø, Norway, 9037.
ORCID: 0000-0001-8681-8521
E-mail: john.osborne@uqconnect.edu.au
